# Logistic regression for estimating functional effects with spatial transcriptomics

**DOI:** 10.1093/nar/gkag466

**Published:** 2026-05-15

**Authors:** Michael Barkasi, Cody Nhan Pham, Demetrios Neophytou, Hysell V Oviedo

**Affiliations:** Department of Neuroscience, Washington University School of Medicine in St. Louis, 660 S. Euclid Ave., 63110 Missouri, United States; Department of Neuroscience, Washington University School of Medicine in St. Louis, 660 S. Euclid Ave., 63110 Missouri, United States; Department of Neuroscience, Washington University School of Medicine in St. Louis, 660 S. Euclid Ave., 63110 Missouri, United States; Department of Biology, The City University of New York Graduate Center, 365 Fifth Ave., 10016 New York, United States; Department of Neuroscience, Washington University School of Medicine in St. Louis, 660 S. Euclid Ave., 63110 Missouri, United States

## Abstract

Spatial transcriptomics (ST) unlocks potential for studying gene functions in processes that depend on orchestration of transcription across space. However, analysis tools for ST remain aimed at data exploration, with few resources for hypothesis testing. What’s missing is a way to test whether a factor of interest affects functionally relevant parameters of a gene’s spatial distribution. We present a tool to fill this gap, which we call a warped sigmoidal Poisson-process mixed-effects (WSP, pronounced “wisp”) model. WSP models are the first ST tool allowing researchers to test critical questions without bespoke preprocessing pipelines for identifying key spatial parameters. By aligning coordinates to an axis of interest and letting a likelihood-based regression find between-group effects on expression rates and boundaries, WSP models replace error-prone manual preprocessing with minimally biased hypothesis testing. After introducing WSP models, we demonstrate their statistical validity using semi-synthetic simulated data and their ability to test for effects by applying them to MERFISH data from mouse somatosensory cortex and bulk sequencing data from mouse liver lobules with extrapolated spatial coordinates. Together, these validations and applications demonstrate that WSP models offer a practical and statistically rigorous approach to quantifying and testing for effects on spatial variation in transcriptomic data.

## Graphical Abstract

**Graphical Abstract:**
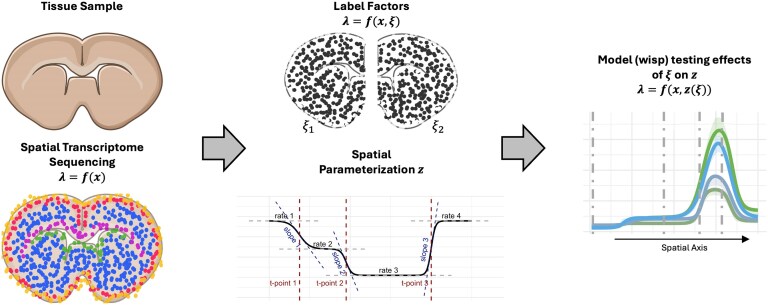

## Introduction

Successful biological and cognitive functioning depends on the spatial distribution of gene expression [1–5]. Identifying factors affecting functionally relevant spatial distributions is thus important for understanding the mechanisms behind such success. However, there are few statistical methods suitable for this purpose. Here, we address this need by presenting a novel computational tool for estimating effects on the spatial distribution of gene expression. We demonstrate its statistical validity by benchmarking on semi-synthetic simulated data and demonstrate utility by applying it to detect effects in two biological cases: the mouse whisker barrel system and radial zonation in liver lobules. Both tissues have known spatial distributions of expression for select genes, and thus also provide additional validation.

### Example biological cases

The mystacial vibrissae are the mouse’s largest and most intricately organized tactile organs, providing rich sensory coding central to how the animal explores and engages with its environment [6–8]. A cardinal feature of the whisker-processing cortex is the barrel field in primary somatosensory cortex (S1), where each vibrissa is represented by a discrete, topographically ordered “barrel.” This map forms early after birth and remains highly plastic, reshaping itself in response to sensory experience [9].

**Figure 1.**
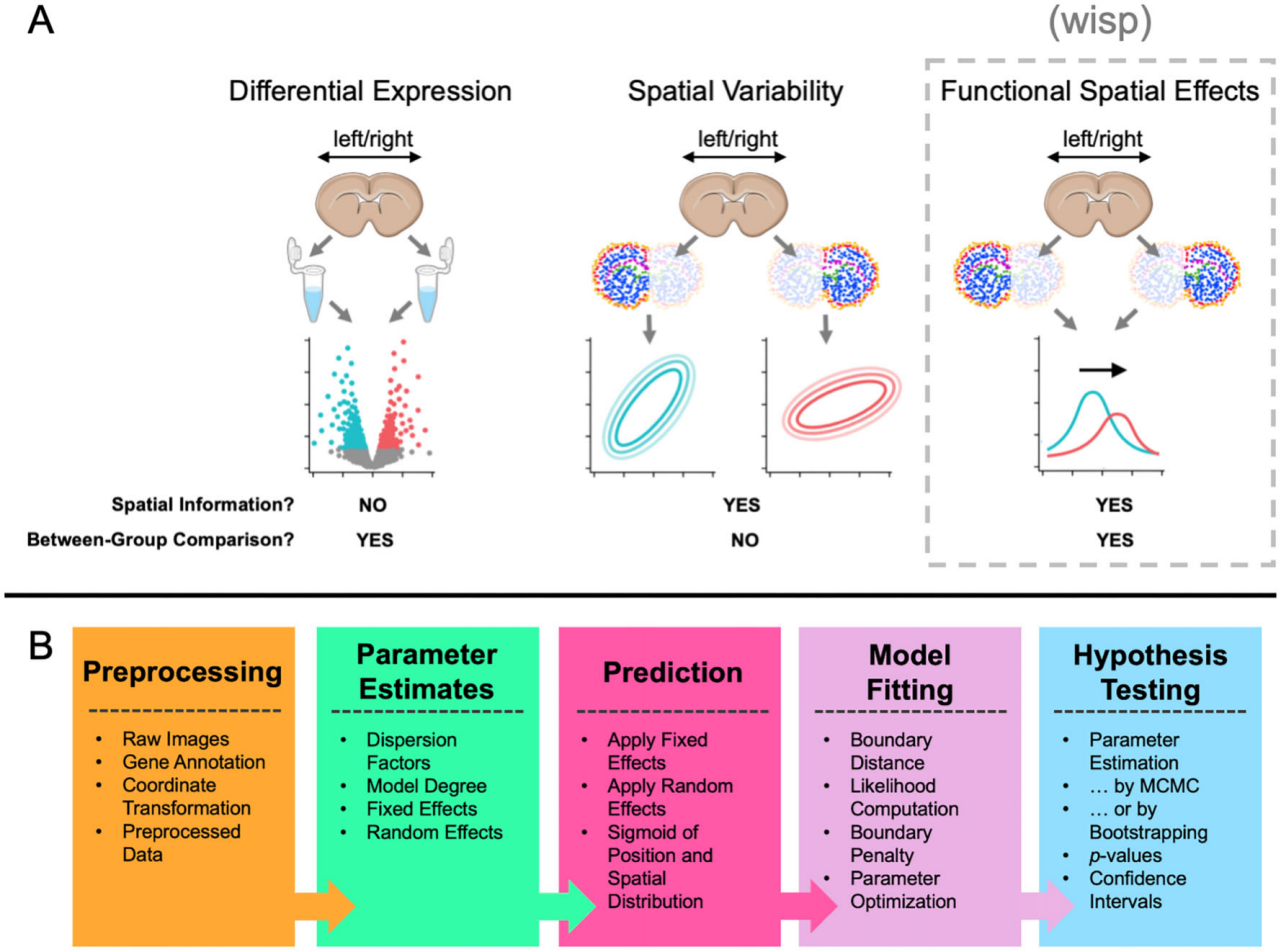
**A**) Schematic difference between analyses of differential expression, spatial variability, and functional spatial effects. A depiction of laterality in the mouse brain is used as an example to represent a between-group factor. Shown from left-to-right are schematics of a volcano plot, spatial covariance plots, and a spatial density plot. (**B**) Pipeline for a WSP model, from data preprocessing to hypothesis testing. Credit: Components of this figure appear in the online documentation (michaelbarkasi.github.io/wispack/). Mouse brain graphic from bioart.niaid.nih.gov/bioart/370, volcano plot graphic from bioart.niaid.nih.gov/bioart/637, both Public Domain.

The development and maintenance of this topographic organization is orchestrated by a localization of the transcription factor ROR$\beta$ in cortical layer 4 (L4) of S1 [10, 11]. Given the importance of this localization, it’s important to know which factors $\xi$—such as age, sex, environmental stimuli, or the expression of other genes—have an effect $\beta _\xi$ on not only the level of ROR$\beta$ expression, but also on the spatial distribution of that expression across the cortex. We call such effects *functional spatial effects* (FSEs, Fig. 1A).

Another potential FSE is the effect of circadian rhythm on radial zonation in liver lobules. Liver cells, called hepatocytes, are organized into more than a dozen concentric rings around central veins [12], with a portal-central radial axis running inward. Blood flows toward the central vein while bile flows outward. These opposed gradients lead to different spatial distributions of genes, which in turn seem related to zonated functions. For example, glutamine synthesis takes place near the central vein, while ureagenesis takes place in the portal zones [13]. As Droin *et al*. [14] point out, in addition to being spatially zonated, lobule function varies with circadian rhythm. Hence, methods for analyzing how temporal rhythm interacts with spatial zonation are needed.

### Modeling FSEs

The data necessary for testing hypotheses related to FSEs can be collected directly through spatial transcriptomics (ST) [15, 16], or indirectly through a combination of bulk sequencing and spatial marker genes (e.g. [14]). ST methods measure gene expression while preserving spatial information, either by detecting transcripts directly *in situ* via hybridization (e.g. seqFISH [17] and MERFISH [18]) or sequencing (e.g. FISSEQ [19] and STARmap [20]), or via arrays or microdissection while using *ex situ* next-generation sequencing (e.g. Visium [21] and Slide-seq [22]). Indirect methods sequence the transcriptome of many cells in bulk, then estimate cell spatial locations by their expression of spatial marker genes. In addition to observing the spatial distribution of genes, new tools for making targeted interventions on the spatial distribution of transcripts are in development as well [23].

However, current computational methods available for analyzing transcript data were not designed to test for FSEs and are limited in their ability to do so. Many methods for transcript analysis (e.g. DESeq2 [24] and limma [25]) were developed for use with bulk and single-cell RNA sequencing (RNA-Seq) data. These methods model expected transcript count $\lambda$, or some other measure of gene expression, as a function $f$ of categorical factors $\xi$:


(1) \begin{eqnarray*}
\lambda =f(\xi )
\end{eqnarray*}

Effects are represented as the difference (i.e. differential expression, DE, Fig. 1 A) between a factor’s reference level and treatment level, which for a linear model is the treatment coefficient $\beta _\xi$. These methods test for effects on $\lambda$ by estimating $\beta _\xi$. If $\lambda$ is “log-linked,” $\beta _\xi$ is a log fold change value as used in standard tools such as the gene ontology (GO) database [26] and Kyoto Encyclopedia of Genes and Genomes (KEGG) [27].

New methods for ST data analysis [28, 29], designed to test for spatially variable genes (SVGs, Fig. 1A), extend the models used in tests for differential expression by including a variable $x$ for spatial position:


(2) \begin{eqnarray*}
\lambda =f(x,\xi )
\end{eqnarray*}

However, testing for FSEs also requires modeling the dependence of the spatial distribution of gene expression on factors $\xi$. If $z$ is a variable parameterizing the spatial distribution, such a model would take the schematic form:


(3) \begin{eqnarray*}
\lambda =f(x, z(\xi ))
\end{eqnarray*}

If a coefficient $\beta _{z\xi }$ is the effect of a factor $\xi$ on the parameterization $z$ of a spatial distribution of $\lambda$ enabling successful biological or cognitive functioning, then $\beta _{z\xi }$ would be a FSE. While coefficients $\beta _{z\xi }$ are not necessarily interpretable as log fold changes, they can be, given the right form for $f$ and a log-link.

When the grain of spatial variation is coarse, this modeling can be done by treating the spatial parameterization as just another categorical factor, i.e. $z= \xi$, without explicit representation of position $x$. For example, effects on the spatial distribution of ROR$\beta$ could be estimated by treating L4 as the treatment level of a position factor in a standard model of differential expression with the schematic form of equation 1. However, this approach requires manual preprocessing of the data with a risk of introducing bias; to define the factor $\xi$, the boundaries of the relevant regions of interest (ROIs) must be specified by hand. Further, it’s plausible that many FSEs will involve spatial properties, such as spatial gradients $\partial \lambda /\partial x$ of expected gene expression, which depend on explicit representation of position and so cannot be represented as categorical factors [30]. In these cases, testing whether the treatment level of a factor $\xi$ has a FSE will require a more complex model with the form of equation 3.

Here we present a model of this form, what we call a *warped sigmoidal Poisson-process mixed-effects model* (WSP model, pronounced “wisp,” Fig. 1B). By explicitly parameterizing the spatial distribution of gene expression within cell types, WSP models not only allow for testing for FSEs on spatial properties requiring a continuous variable, such as gradients, but also eliminate the need for manual parsing of ROIs. Additionally, WSP models provide estimates of effects on expression rate $\lambda$ in the form of log fold changes. We have made available a R package, *wispack* (pronounced “wisp package” or “wisp pack”), which implements WSP models in Rcpp. A minimal working example showing how to use wispack is given below (Appendix 3) and extensive documentation is available online: michaelbarkasi.github.io/wispack/.

### Evaluating WSP models

To evaluate the statistical validity of WSP models, we benchmarked wispack on semi-synthetic simulation data. Specifically, we compared the false-positive rate (FPR), false-discovery rate (FDR), and power for wispack against DESeq2 [24] and ELLA [31]. Although DESeq2 is intended for DE testing and ELLA for SVG testing, they still provide an insightful comparison.

To evaluate utility, we applied wispack to two biological examples. First, using our own MERFISH data, we tested for effects of age and laterality on ROR$\beta$ expression in S1 of male wild-type (WT) mice, in glutamatergic and GABAergic cells. Five other genes known to have layer-specific expression (Table 1) were tested along with ROR$\beta$. Second, we used wispack to replicate some of the time-series analysis (six genes, Table 2) done by Droin *et al*. [14] on interactions between temporal rhythm and spatial zonation in liver lobules.

**Table 1.** Modeled layer-specific genes and their known layers of expression in the neocortex

Abbreviation	Name	Layer	Reference
SATB2	SATB Homeobox 2	L2/3	[32]
CUX2	Cut Like Homeobox 2	L2/3/4	[32–34]
ROR$\beta$	Retinoic acid-related orphan receptor beta	L4	[10, 11, 34]
FEZF2	FEZ Family Zinc Finger 2	L5	[35, 36]
BCL11B, CTIP2	B-cell CLL/lymphoma 11b	L5/6	[32, 35, 36]
NXPH3	Neurexophilin 3	L6	[37]

**Table 2.** Modeled zonated and rhythmic genes and their known expression profiles in the liver, reported by [14]

Abbreviation	Name	Zonation	Rhythm
GLUL	Glutamine synthethase	Central	
ASS1	Urea cycle gene argininosuccinate synthetase	Portal	
ARNTL, BMAL1	Basic helix-loop-helix ARNT like 1		Circadian
DBP	PAR bZip transcription factor		Circadian
ELOVL3	PAR bZip transcription factor	Central	Circadian
PCK1	Phosphoenolpyruvate carboxykinase 1	Portal	Circadian

To demonstrate how WSP models can be used to test hypotheses, we formulated and tested a hypothesis concerning ROR$\beta$. Specifically, we hypothesized that age and laterality would have significant effects on ROR$\beta$ expression based on (i) our previous observations of interacting effects from these factors on electrophysiological recordings of the developing auditory cortex (ACx) of mice [38–40] and (ii) histological observations by other labs suggesting the possibility of lateralized development in S1 as well [10, 11, 41]. Our concluding discussion outlines interesting ways the FSEs estimated by the WSP model potentially inform current work on ROR$\beta$ and rodent whisker-barrel development.

## Materials and methods

### Modeling

#### Transcript count distribution

Transcript production in a single cell is a stochastic process varying between periods of quiescence and active bursting [42–44]. This variation leads to observed transcript counts $y$ with an over-dispersed Poisson distribution. Consistent with normal practice [29, 45, 46], WSP models handle this stochastic behavior by assuming $y$ is from a Poisson distribution:


(4) \begin{eqnarray*}
y\sim \mathrm{Pois}(\Lambda )
\end{eqnarray*}

with a stochastic rate $\Lambda$ from a gamma distribution, i.e. a gamma kernel:


(5) \begin{eqnarray*}
\Lambda \sim \mathrm{Gam}(\lambda , {\sigma _{\gamma }^2})
\end{eqnarray*}

The gamma kernel is parameterized in terms of its expected value $\lambda$ and variance ${\sigma _{\gamma }^2}$, as these are interpretable in terms of expected transcript count, $\Lambda$. While $\Lambda$ is not itself directly observable, it is some unknown random gamma-distributed deviation with variance ${\sigma _{\gamma }^2}$ from $\lambda$. In the standard parameterization of the gamma distribution in terms of “rate” ($\gamma _{\mathrm{rt}}$) and “shape” ($\gamma _{\mathrm{sp}}$), $\gamma _{\mathrm{rt}}= \lambda /{\sigma _{\gamma }^2}$ and $\gamma _{\mathrm{sp}}= \lambda ^2/{\sigma _{\gamma }^2}$. Note that modeling transcript count distribution as a gamma-convolved Poisson distribution is mathematically equivalent to using a negative binomial distribution. For clarity, we refer to $\lambda$ as the *kernel rate* and $\Lambda$ as the *transcription rate*. Table 3 provides a glossary of the mathematical notation used here.

**Table 3.** Glossary of notation

Symbol	Definition130	Eq.	Symbol	Definition	Eq.
**Functions**					
$f$ , $X$, $Y$	Generic function, generic inputs		$\mathrm{log}_e$ , $e^{X}$	Natural log, exponentiation of its base	
$\mu$ , $\sigma$, $\sigma ^2$	Mean, standard deviation, variance		$\Psi$ , $\psi$	WSP sigmoid and logistic functions	10, 9
$\omega$ , $\varphi$	Warping and warping ratio functions	20, 19	$w$	Fixed-effect treatment interaction	11
$\left|{\cdot }\right|_{\#}$ , $|{\cdot }|_{\mathrm{abs}}$	Set cardinality, absolute value		$\Gamma$	Gamma function	
**Scalar Values**					
$x$	Position		$y$	Observed transcript count	4
$\lambda$ , $\Lambda$	Kernel, transcription rate	8, 5	$b$	Warp bounds	
$\zeta$	Dispersion factor	7	$\mathbf {x}$ , $\mathbf {b}$, etc	Geometric coordinate transform values	
**Probability Distributions**					
$\mathrm{Pois}$ , $\mathrm{Gam}$	Poisson, gamma distributions		${\sigma _{\gamma }^2}$	Gamma distribution variance	A.17
$\mathcal {N}$ , $\mathrm{Binom}$	Gaussian, binomial distributions		$\gamma _{\mathrm{rt}}$ , $\gamma _{\mathrm{sp}}$	Gamma distribution rate, shape	
$\ell$ , $L$, $\mathrm{P}$	Likelihood, log likelihood, probability				
**Vectors and Matrices**					
$z$	Model spatial parameter, i.e., $r$, $s$, or $p$	22	$r$ , $s$, $p$	Rate, t-point slope scalar and location	
$\Phi$	Model effect, e.g., $\beta$, $\rho$		$\beta$ , $\rho$	Fixed, Random effect	
**Indices**					
$i$ , $j$	Coordinate blocks, treatment levels		$h$ , $k$	Fixed-effect, random-effect factors	
$d$	WSP model degree		$q$	Spatial parameters	

To a first approximation, the aim of fitting a WSP model is to estimate a kernel rate $\lambda$ and variance ${\sigma _{\gamma }^2}$ which maximize the likelihood $\ell (y\, |\, \lambda ,{\sigma _{\gamma }^2})$ of the observed counts. WSP models allow for both $\lambda$ and ${\sigma _{\gamma }^2}$ to depend on RNA species $g$, i.e. gene, and cell type ${c}$. For all combinations of gene $g$ and cell type ${c}$, the kernel rate $\lambda$ is estimated via a prediction from a function $f$ of spatial position. Following equation 3, $f$ is a function of both spatial position $x$ and some parameterization $z$ of the distribution of transcripts across $x$ which is potentially affected by factors $\xi$. The heart of the WSP modeling approach consists in this spatially parameterized function and will be explained below (equation 10).

Following other standard approaches [24], we assume the variance ${\sigma _{\gamma }^2}$ is given in terms of a *dispersion factor*, $\zeta$, specifying the extent to which $y$ is over-dispersed relative to a Poisson distribution:


(6) \begin{eqnarray*}
{\sigma _{\gamma }^2}= \lambda + \zeta \lambda ^2
\end{eqnarray*}

This dispersion factor will potentially vary between genes $g$ and cell types ${c}$. As our focus is on the prediction of $\lambda$, we estimate the dispersion factor $\zeta (g,{c})$ with a simple empirical approximation. Specifically, we apply equation 6 to the mean $\mu _{y}(g,{c})$ (representing $\lambda$) and variance $\sigma ^2_{y}(g,{c})$ (representing ${\sigma _{\gamma }^2}$) of the observed counts $y$ of gene $g$ in cell type ${c}$:


(7) \begin{eqnarray*}
\zeta (g,{c}) = \frac{ \sigma ^2_{y}(g,{c}) }{ \mu _{y}(g,{c})^2 } - \frac{ 1 }{ \mu _{y}(g,{c}) }
\end{eqnarray*}

#### Spatial parameterization

The fundamental biological assumption behind WSP models is that gene expression within a given cell type across a spatial region of tissue has a constant kernel rate $\lambda$, except for transition points where that rate changes. The transcription rate itself will, of course, vary stochastically from point-to-point. A similar assumption of discontinuous rate regions is made by GASTON [47], which models gene expression across a single axis piecewise with linear models. WSP models capture this assumption by predicting log-linked kernel rate for gene expression as a function $\Psi$ of spatial position $x$ and three spatial-distribution parameters $z=\langle z_1,z_2,z_3\rangle$, i.e., rates $r=z_1$, transition-point slope scalars $s=z_2$, and transition-point positions $p=z_3$:


(8) \begin{eqnarray*}
\mathrm{log}_e{}(\lambda + 1) = \Psi _{d}(x,r,s,p)
\end{eqnarray*}

**Figure 2.**
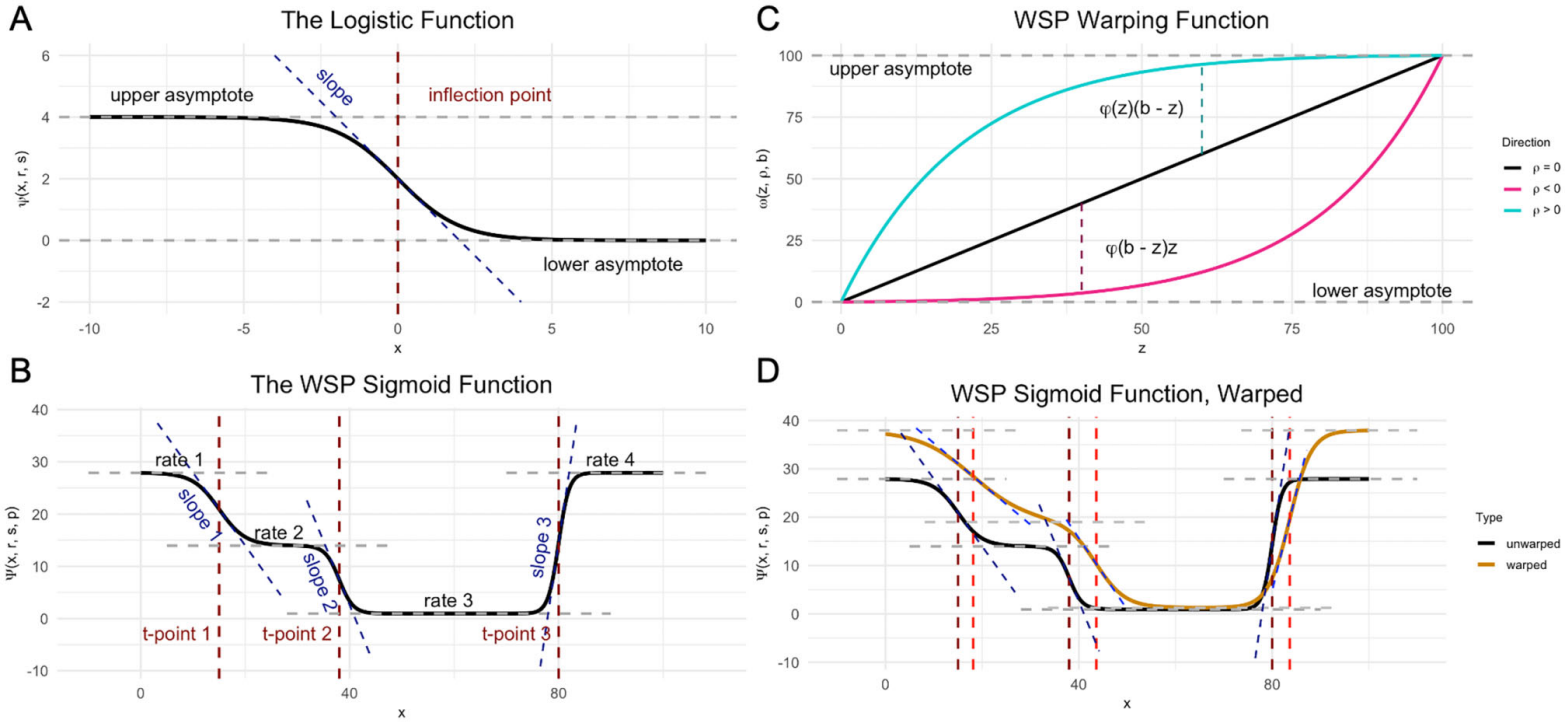
Examples of WSP model and WSP model-related functions, with parameters shown as colored dashed lines. (**A**) The logistic function. (**B**) A degree-three WSP sigmoid. (**C**) The warping function $\omega$ with positive, negative, and zero warp. (**D**) A WSP sigmoid warped by the warping function $\omega$. Light-colored dashed lines show the displacement of the spatial parameter values under the warp. Credit: Components of this figure appear in the online documentation (michaelbarkasi.github.io/wispack/).


A specific variety of sigmoid, the logistic function (Fig. 2A), provides the building blocks for the case when $x$ is a point in one-dimensional space:

(9) \begin{eqnarray*}
\psi (x,r,s) = \frac{r}{1 + e^{sx}}
\end{eqnarray*}

In this equation, $\psi$ is a sigmoid with inflection point of slope $s(\psi r- \psi ^2)/r$ at zero which toward $-\infty$ asymptotes at $r$ and toward $\infty$ asymptotes at zero [48].

While the restriction to one dimension is a limitation, many potential FSEs play out over a single axis, e.g. the laminar axis across the cortex or the radial axis out from a cell nucleus or other concentric structure (e.g. liver lobules). When using ST data from a tissue slice of negligible thickness, the spatial coordinates can be transformed into a pair $\langle x,x^\bot \rangle$ of orthonormal axes, or pair $\langle x,\theta \rangle$ of polar coordinates, the first of which is defined by the functionally relevant physical axis of interest. If $x$ is discretized into bins, the count $y_x$ observed at some position $x$ would be the sum of all transcripts within bin $x$ with any coordinate $x^\bot$ or $\theta$. The additional variance from this aggregation becomes just another source of over-dispersion modeled by the gamma kernel.

Logistic functions are useful for modeling a variable with the features of $\lambda$ because of their behavior when summed, a behavior which, to the best of our knowledge, has not been exploited in regression modeling. Assume $s$ and $p$ are real-valued vectors of length $d$ and assume $r$ is a real-valued vector of length $d+ 1$ and define, for $d> 0$:


(10) \begin{eqnarray*}
\Psi _{d}(x,r,s,p) &= r_1 + \sum _{i=1}^{d}\psi (x - p_i, r_{i+1} - r_i, -s_i) \\&= r_1 + \sum _{i=1}^{d} \frac{ r_{i+1} - r_i}{ 1 + e^{-s_i(x- p_i)} }
\end{eqnarray*}

Call $d$ the *degree* of $\Psi _{d}$ and define $\Psi _0=r_1$. Each gene $g$ within each cell type ${c}$ is modeled with its own function $\Psi _{d(g,{c})}$, with $d(g,{c})$ being the number of kernel-rate transition points for $g$ within cell type ${c}$—loosely speaking, the number of expression-rate transition points.

The function $\Psi _{d}$ maps inputs $x$ to constant outputs $r$, except for $d$ points $p$ at which $r$ transitions with slope scaled by $s$ (Fig. 2B). Specifically, using $i$ to index the elements of $r$, $s$, and $p$, if $s_i\gg 0$ for all $i$ and $i^\prime$ is the largest $i$ such that $x\gg p_i$, then $\Psi _{d}(x,r,s,p) \approx r_{i^\prime +1}$ (Appendix 4.1). Hence, the value $r_i$ can be thought of as the predicted rate for the block of points $x$ such that $p_{i-1}< x< p_{i}$, with $p_{0}=0$ and $p_{d+1}=\mathrm{max}(x)$. In addition, it can be shown that the slope $\partial \Psi _{d}/\partial x$ at $x=p_i$ is approximately $(r_{i+1}-r_i)s_i/4$ (Appendix 4.2). Note that a transition point with a lower slope scalar does not necessarily have a more shallow slope, as the magnitude of the slope can go up even as the scalar goes down, provided the magnitude of the rise $r_{i+1}-r_i$ increases proportionately more.

All three spatial parameters have a lower bound of zero. Log-linked rate $r$ cannot go below zero because of the added one (equation 8), as that would entail negative transcript counts. The slope scalar $s$ does not control the sign of transition point slopes (which is controlled by the rise) and, as implied by the sigmoid rate theorem (Appendix 4.1), cannot go below zero without disrupting the relationship between $\Psi$ and $r$. While the spatial coordinate $x$ and the transition points $p$ can be any real value, we assume for simplicity that the modeled axis $x$ is finite and bound by zero and some upper limit $b_{p}=\mathrm{max}(x)$.

#### Fixed effects

The aim of WSP models is to test for *fixed effects* on gene expression, i.e. effects from systematic, reproducible factors $\xi = \langle \xi _1,\ldots ,\xi _n\rangle$ which often divide observations into two classes, a reference class and a “treatment” class. Reference and treatment classes could be defined by a deliberate intervention, e.g. comparing wild-type animals to genetic knock-outs, but could also be any comparison of interest, e.g. comparing old and young animals. These factors are handled just as in a traditional linear model. Each variable $\xi _h$ can take one of two values, zero or one. Hence, $\xi \in \lbrace 0,1\rbrace ^n$. The model considers all possible unique treatment interactions $w_j= \xi _{j_1}\times \ldots \times \xi _{j_m}$, for $m$ such that $0\le {}m\le {}n$. Each treatment defines a weight function $w_j:\lbrace 0,1\rbrace ^n\rightarrow \lbrace 0,1\rbrace$ by multiplication:


(11) \begin{eqnarray*}
w_j(\xi ) = \xi _{j_1}\cdots \xi _{j_m}
\end{eqnarray*}

By convention, the first treatment interaction $j=1$ is the case when $m=0$, i.e., no treatment. In this case, the absolute reference level, $w_j(\xi ) = 1$ for all $\xi$. For each $z_q$ (i.e. for each of $r$, $s$, and $p$), each such interaction $w_j$ will have, for each gene $g$ and cell type ${c}$, a corresponding effect value $\beta _{qij}(g,{c})$ for each element $z_{qi}(g,{c},\xi )$ of $z_q(g,{c},\xi )$ such that:


(12) \begin{eqnarray*}
z_{qi}(g,{c},\xi ) = \sum _{j} w_j(\xi ) \beta _{qij}(g,{c})
\end{eqnarray*}

While fully specifying a fixed effect requires specifying $g$, ${c}$, $\xi$, $q$, and $i$, we will only write what is necessary for clarity and will also use obvious substitutions, e.g. writing “$\beta _{r}$” for $\beta _{q}$ when $q=1$. We adopt this shorthand for not only fixed effects, but all variables.

Given that $r$ is, when sufficiently far from the transition points $p$, the log of the predicted kernel rate $\lambda$ plus one (Appendix 4.1), the rate effects $\beta _{r}$ are approximately multiplicative and interpretable as log fold changes. That is, if $\beta _{r{i{1}}},\ldots ,\beta _{r{i{l}}}$ are the rate effects for block $i$ for each of the $l$ possible treatment conditions $w_j$, then the predicted kernel rate (plus one) of a sample in block $i$ is approximately the product:


(13) \begin{eqnarray*}
\lambda _i+ 1 = e^{\Psi _{d}}\approx e^{w_1\beta _{r{i{1}}}} \cdots e^{w_l\beta _{r{i{l}}}}
\end{eqnarray*}

In this equation, $w_1=1$ and the other $w_j$ are zero or one, depending on whether the sample falls under treatment condition $j$. Equation 13 implies that:


(14) \begin{eqnarray*}
w_2\beta _{r{i{2}}} + ... + w_l\beta _{r{i{l}}} \approx \mathrm{log}_e\left( \frac{ \lambda _i+ 1 }{ e^{w_1\beta _{r{i{1}}}} }\right)
\end{eqnarray*}

That is, the sum of the rate effects $\beta _{r}$, excluding the baseline case of $j=1$, is the natural-log fold change of the predicted kernel rate $\lambda$ (plus one), the “fold” being with respect to the no-treatment reference condition of $j=1$. To get the more conventional log-2 fold change value, simply divide this sum by $\mathrm{log}_e(2)$. Hence, the log-2 fold change associated with the treatment conditions on kernel rate (plus one) in block $i$ is given as:


(15) \begin{eqnarray*}
\mathrm{log}_2\left( \frac{ \lambda _i+ 1 }{ e^{w_1\beta _{r{i{1}}}} }\right) \approx \frac{ w_2\beta _{r{i{2}}} + ... + w_l\beta _{r{i{l}}} }{ \mathrm{log}_e(2) }
\end{eqnarray*}

This ability to extract log-2 fold changes makes it easy to incorporate WSP models into a pipeline using standard tools such as Go [26] and KEGG [27].

Transition points $p$ and slope scalars $s$ are not transformed by the log-link (equation 8), and so the effects $\beta _{p}$ and $\beta _{s}$ on them are additive. Thus, they cannot be interpreted as log fold changes, but can be read straight off equation 12. Regardless of the log-link, for $j\ne 1$, effects for rate $r$, transition point $p$, and slope scalar $s$ are all interpreted in the usual way: zero is no effect, negative values represent a decrease in value, and positive values represent an increase. Thus, when $j\ne 1$, fixed effects $\beta _{qij}$ can be modeled as coming from a normal distribution centered on zero. When $j=1$, while treated the same as other effects in equation 12, $\beta _{qij}$ gives the value of $z_q$ in the reference level and so must follow the distribution of the spatial parameters, i.e. $\beta _{qi1}> 0$ and $\beta _{3ij}=p\le b_{p}$.

#### Time series

WSP models assume that all fixed-effects are binary, i.e., take values from $\lbrace 0,1\rbrace$, except for times series. Within a WSP model, time series are handled as an ordered series of additive binary fixed-effects. Specifically, given time points:


(16) \begin{eqnarray*}
T_1 < T_2 < ... < T_n
\end{eqnarray*}

we define a time-factor series $\xi _T$ with $n$ ordered binary factors:


(17) \begin{eqnarray*}
\xi _{T_1} < \xi _{T_2} < \ldots < \xi _{T_n}
\end{eqnarray*}

Assuming other fixed-effect factors $\xi _F$ each with effects $\beta _{F}$, the effect of time $\xi _{T_m}$ from $\xi _T$ on spatial parameter $z$ will include the effect $\beta _{T_m}$ from $\xi _{T_m}$ itself, plus an interaction term $\beta _{T_m\times \xi _F}$ for all $\xi _F$, as well as the effects $\beta _{T_l}$ from times $\xi _{T_l}$ for all $l< m$ and their respective interaction terms $\beta _{T_l\times \xi _F}$:


(18) \begin{eqnarray*}
z \mapsto z + \sum _{l=1}^{n} \left(\beta _{T_l}\xi _{T_l} + \sum _{F}\beta _{T_l\times \xi _F}\xi _{T_l}\xi _F\right)
\end{eqnarray*}

This formula works to capture the additive nature of time-series effects, so long as we stipulate that, if $\xi _{T_m}=1$ for some sampled data point, then for that data point $\xi _{T_l}=1$ for all $l< m$.

#### Random effects

Hypothesis testing requires distinguishing variation between observations due to *fixed effects*$\beta$ from variation due to random noise, i.e. *random effects*$\rho$. Normalization is sometimes used to filter noise, but requires knowing ahead of time which factors $\nu$ represent true noise and knowing that these factors do not correlate by chance with an effect of interest. For example, analysis pipelines for RNA-Seq data sometimes normalize total transcript count, but this is not recommended when testing for SVGs, as total transcript count can correlate with spatial variance [49, 50]. An alternative approach, *mixed-effects modeling* [51], used in some models of differential gene expression [24] and other areas of genomics [52] and neuroscience [53], is to include the noise variables $\nu$ (*random-effect factors*) in the model.

WSP models include a single random-effect factor $\nu$ the levels of which might represent, for example, different biological specimens or different runs of transcript measurement. For this single random-effect factor, WSP models assign a random effect for each of the three spatial parameters, i.e. a random effect $\rho _{r}$ on rate, $\rho _{s}$ on transition-point slope scale, and $\rho _{p}$ on transition-point position. These random effects are relative to a pseudo reference level (“none,” i.e. no random effect) extrapolated from the data by taking the mean of the log-linked count $\mathrm{log}_e(y+1)$, observed in each $x\times g\times \xi$ combination, across all random-effect levels. Note that this means that while fixed effects are assumed to vary independently across cell types ${c}$, random effects are assumed to be constant across cell types and specific to individual genes $g$. Random effects are applied to spatial parameters after computing those parameters from the fixed effects (equation 12) but before computing the predicted log-linked count via the WSP model function $\Psi$ (equation 10, Fig. 1B).

Including random-effect factors in the model allows for estimating the individual contributions of fixed and random effects by modeling the interaction of fixed-effect and random-effect factors. For each level $k$ (taking values zero or one) of a random-effect factor $\nu$, a WSP model assigns each gene $g$ a random effect $\rho (g,k)$. The process of fitting the model implicitly estimates the individual contributions of fixed and random effects by, for each $g$ and ${c}$, finding the values $\beta _{q}(g,{c})$ and $\rho _{q}(g,k)$ which yield the best fit to the observed data.

Traditional linear mixed-effects models treat random effects as just another value to be added in the sum giving the prediction (equation 12). However, for WSP models each $z_q$ has a lower bound of zero and should be smoothly skewed asymptotically toward that bound by negative $\rho _{q}$. In addition, the upper limit $b_{p}$ of position and transition points imposes a bound on positive $\rho _{p}$, such that those effects should also smoothly skew transition points toward this bound asymptotically.

This asymptotic “warping” behavior can be achieved for positive $\rho$ by adding to $z_q$ an asymptotically increasing ratio $\varphi$ of the distance $b - z_q$ to the upper boundary, so long as $\varphi$ scales with $z_q$. Conversely, subtracting from $z_q$ that same ratio $\varphi$ of $z_q$ itself will achieve the desired warping behavior for negative $\rho$, so long as $\varphi$ scales with the distance $b - z_q$ to the upper boundary (Fig. 2C). There are many such functions $\varphi$, but the following behaves well:


(19) \begin{eqnarray*}
\varphi (X,\rho ,b) = 1 - ({e^{\rho ^2}})^{-X/b}
\end{eqnarray*}

With this equation, the random effects are defined:


(20) \begin{eqnarray*}
\omega (z_q,\rho _{q},b_{q}) = \left\lbrace \begin{array}{@{}l@{\quad }l@{}}z_q+ \varphi (z_q, \rho _{q},b_{q})(b_{q} - z_q) & \text{if } {\rho _{q}} \ge 0 \\z_q- \varphi (b_{q} - z_q,\rho _{q},b_{q})z_q& \text{if } {\rho _{q}} < 0 \end{array}\right.
\end{eqnarray*}

It is easy to see from equation 19 that, when $\rho =0$, $\varphi =0$ and so $\omega (z_q,\rho _{q},b_{q})=z_q$. Thus, as expected, a random effect of zero leads to no warp. Less obviously (see Appendix 4.3), when $b\rightarrow \infty$, the warping function reduces to a simple linear function of $z_q$:


(21) \begin{eqnarray*}
\lim _{b_{q}\rightarrow \infty }\omega (z_q,\rho _{q},b_{q}) = \left\lbrace \begin{array}{@{}l@{\quad }l@{}}z_q(1 + (\rho _{q})^2) & \text{if } {\rho _{q}} \ge 0 \\z_qe^{-(\rho _{q})^2} & \text{if } {\rho _{q}} < 0 \end{array}\right.
\end{eqnarray*}

Thus, as expected, when there is no upper bound there is no need to “warp,” i.e. curve, the input. In this case, which applies for rate $r$ and slope scalar $s$, positive random effects $\rho$ become linear scaling factors.

Each possible combination $g\times {c}\times \xi \times k$ of gene $g$, cell type ${c}$, fixed-effect factors $\xi =\langle \xi _1,\ldots ,\xi _n\rangle$, and random-effect level $k$ determines, for each spatial parameter $z_q$, a vector:


(22) \begin{eqnarray*}
z_q(g,{c},\xi ,k) = \langle \omega (z_{qi}(g,{c},\xi ), \rho _{q}(g,k), b_{q}) \rangle _{i=1}^{d(g,{c})} \\
\end{eqnarray*}

A full WSP model uses this equation to compute the parameterizing inputs $z_1=r$, $z_2=s$, and $z_3=p$ for equation 10 (Fig. 1B).

#### Model fitting and statistical methods

Fitting a WSP model to observed counts $y$ requires three steps: estimate the degree $d$ for each gene $g$ within each cell type ${c}$, compute initial values for all parameters, and optimize these initial values based on model fit. Degree estimation is done via a bespoke likelihood-ratio outlier (LRO) change-point detection algorithm [54] (Appendix 1.1). The change-points detected when estimating degree are extended into initial estimates of all parameters (Appendix 1.2). The initial values are optimized by using gradient descent (L-BFGS) to find the effects parameters $\beta$ and $\rho$ which, given the estimated dispersion factors $\zeta$ (equation 7), maximize the joint likelihood of the observations within the log-link (Appendix 1.3).

Confidence intervals (CIs) and $p$-values are estimated empirically via resampling, either by a Metropolis–Hastings random walk for Markov chain Monte Carlo (MCMC) estimation or by bootstrapping across cells (Appendix 2). CIs can be computed in this way for all parameters $\beta$ and $\rho$, while $p$-values can only be computed for fixed effects $\beta _{j}$ for $j\ne 1$ and random effects $\rho$, as these parameters center on zero. In practice, wispack does not compute $p$-values for random effects. Adjustment for multiple comparisons is handled by default with a Holm–Bonferroni correction, although wispack offers a setting for the more conservative Bonferroni correction as well.

### Benchmarking on simulations

To demonstrate statistical validity, we used semi-synthetic simulations to compute FPR, FDR, power, and correlations between estimated and true effects. Run time was tracked as well. For comparison, we benchmarked DESeq2 [24] and ELLA [31] along with wispack.

#### Attractor simulations

**Figure 3.**
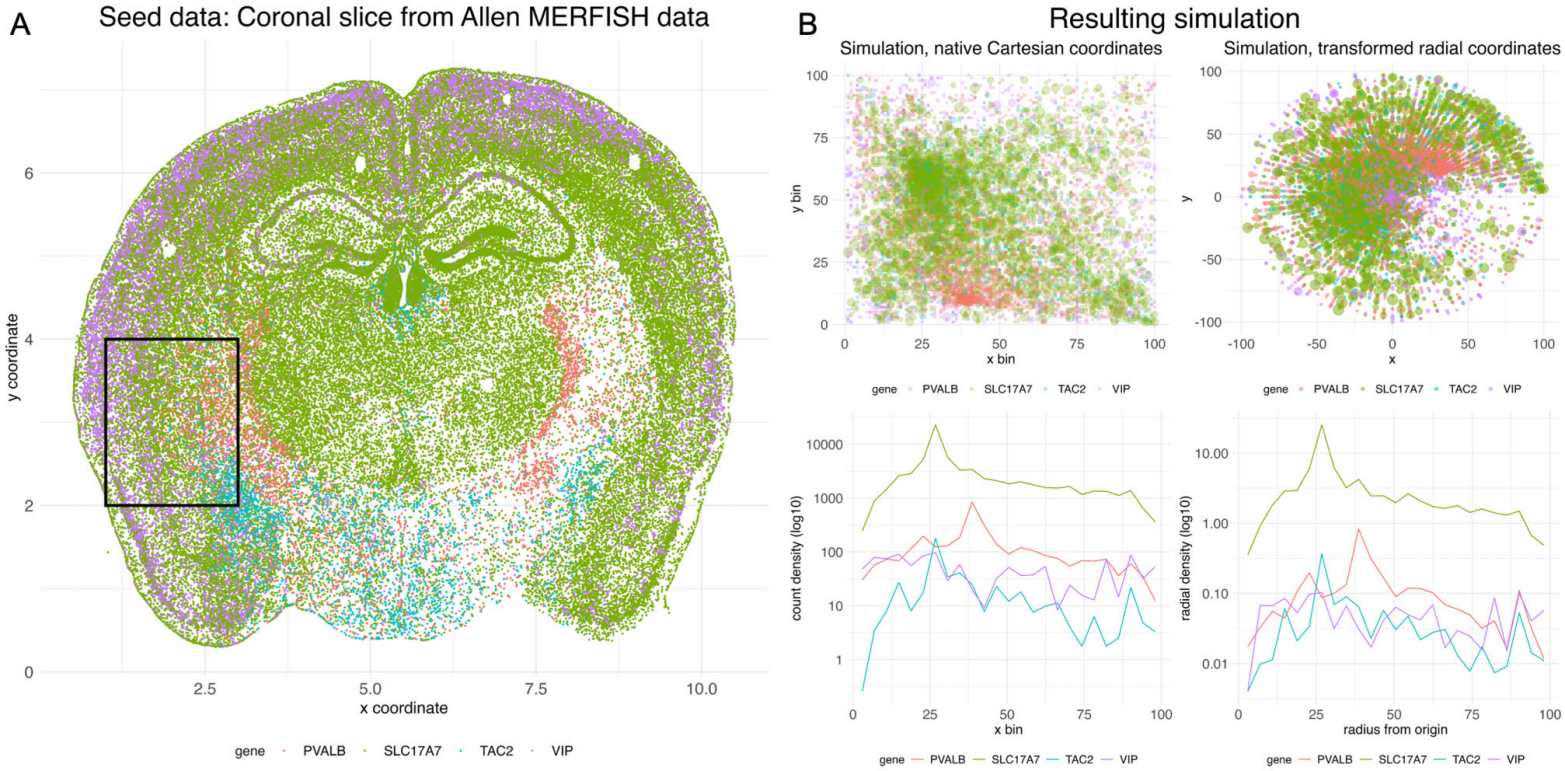
Attractor simulations and seed data. (**A**) Slice 33 of the raw MERFISH data from Yao *et al*. [55]. The $2\times 2$mm patch used to seed all simulations is within the black box. (**B**) Simulated data based on the seed patch. Top left: Simulated transcript counts for each gene in their native Cartesian coordinates. Top right: The same data, but with coordinates transformed so that density along the radial axis out from the origin matches density along the *x*-axis in the native coordinates. Bottom: Plots showing the density of the simulated transcript counts for the native coordinates (left) and radially transformed coordinates (right) showing that the radial transformation preserves the count distribution. Credit: Components of this figure appear in the online documentation (michaelbarkasi.github.io/wispack/).

The function attractor_simulation from wispack, described fully in the documentation (michaelbarkasi.github.io/wispack/articles/tutorial_benchmarks.html), was used to make the simulations. This function takes seed data and uses it to produce sample replicates from reference and treatment conditions. For this benchmarking, we used the raw MERFISH data from Yao *et al*. [55] as the seed (Fig. 3A). These data include counts-by-cell for five hundred genes from coronal slices of an adult WT mouse. We used a single $2\times 2$mm patch from slice 33 with four genes: PVALB, SLC17A7, TAC2, and VIP. No attempt to save or simulate cell types was made for these simulations or benchmarking.

The aim of starting with real data was to have a realistic distribution of transcript counts per cell. In the seed data, each cell has a single 3D Cartesian spatial coordinate (2D after dropping the coordinate specifying the slice position) and a count for each gene. To make a simulation, the attractor_simulation function first randomly shuffles these coordinates without regard for cell identity, yielding a statistically smooth distribution of gene counts across the patch. Next, at least one, and potentially all four, of the genes are randomly chosen to be spatially variable. Spatial variability is induced on the selected genes by pulling spatial coordinates toward a randomly chosen attractor point (Fig. 3B, left). From among the SVGs, some (potentially none) are randomly selected to have a randomly chosen non-null fixed effect (up or downregulation of rate) of at least $5\%$ in the treatment condition. Finally, replicates are produced for both reference and treatment conditions through a combination of affine coordinate transformations (to induce spatial variation) and rate scaling (to induce rate variation). For this benchmarking, we used four replicates total, simulating observations from both the reference and treatment condition for each replicate.

The end result is that, for each simulation run from the seed, we knew which of the four genes were SVGs, the treatment effect $\beta$ (if any) for those SVGs, and the random effect $\rho$ on the rate of each replicate. Note that these simulations generate spatial variability, fixed effects, and random effects completely independent of any of the mathematical functions used in a WSP model. Thus, there is no inherent bias toward WSP models being good at recovering simulation parameters.

#### Benchmarking wispack

Wispack includes a function for fitting a WSP model to an attractor simulation. This function, named model_attractor_simulation_wisp, always performs hypothesis testing via bootstraps, with the number of bootstrap resamples being adjustable. For benchmarking, we used 1000 bootstraps per simulation. For each simulation and gene, model_attractor_simulation_wisp returns estimated and true (i.e. stipulated) values for the rate effect $\beta$ and random effect $\rho$, as well as, for each gene, whether it exhibited a FSE in the treatment condition and the $p$-value wispack gave for its estimated FSE.

In wispack, rate effects $\beta _{r}$ apply not to a gene equally across space, but instead to constant blocks of expression rate separated by transition points $p$. However, in attractor simulations, rate effects are constant across space. To accommodate this mismatch, the function model_attractor_simulation_wisp uses the mean of all rate effect estimates $\beta _{r}$ as the WSP model estimate of the simulated rate effect, and uses the mean of the $p$-values divided by the number of rate blocks as the WSP model $p$-value for FSE tests. We divide by the number of rate blocks because wispack treats block rate effects as independent, while attractor simulations use a single rate effect.

Thus, for wispack, we compared estimated vs true fixed and random rate effects, as well as quantified FPR, FDR, and power for FSE testing. For each gene $g$ in each simulation and $\alpha = 0.05$, the model_attractor_simulation_wisp function treats a FSE test result as a false positive ($\mathrm{FP}$) if the $p$-value is $< \alpha$ and $g$ is a true null-effect, as a true negative ($\mathrm{TN}$) if the $p$-value is $\ge \alpha$ and $g$ is true null-effect, as a true positive ($\mathrm{TP}$) if the $p$-value is $< \alpha$ and $g$ is a true non-null effect, and as a false negative ($\mathrm{FN}$) if the $p$-value is $\ge \alpha$ and $g$ is a true non-null effect. The FPR is thus computed as $\mathrm{FP}/(\mathrm{FP}+\mathrm{TN})$, the FDR as $\mathrm{FP}/(\mathrm{FP}+\mathrm{TP})$, and power as $\mathrm{TP}/(\mathrm{TP}+\mathrm{FN})$.

#### Benchmarking DESeq2

DESeq2 models gene expression across treatment conditions using a generalized linear model with shrinkage estimation (generalized ridge regression) to handle random variation [24]. It has no way to handle spatial position, aside from treating it as a discrete treatment factor. Hence, we applied DESeq2 to the attractor simulations by summing gene counts across spatial positions and within replicates and treatment conditions, yielding eight data points per gene.

The DESeq function itself was run with all defaults, except fitType was set to mean and minReplicatesForReplace was set to 3. We found that these were the only workable settings for the low number of genes and low number of replicates in the attractor simulations. The lfcShrink function was applied to the results using all defaults, including using the adaptive Student’s *t* prior shrinkage estimator from the apeglm package [56]. The lfcShrink function allows for passing optional arguments to the apeglm function, including a scalar for the adaptive shrinkage and values to use fixed priors instead of adaptive shrinkage. These settings allow for adjusting the strength of the regularization of the shrinkage. However, after trying various *ad hoc* combinations of adjustments and finding no improvement to FPR, FDR, and power, we settled on leaving all shrinkage settings at default.

Both estimates of log-2 fold change (for reference vs treatment condition) and adjusted $p$-values were taken from the lfcShrink output. We compared estimated vs true fixed rate effects and quantified FPR, FDR, and power for FSE testing, using the same definitions used for wispack.

#### Benchmarking ELLA

While DESeq2 performs DE testing, ELLA performs SVG testing. In contrast to DESeq2, which makes comparisons across treatment conditions without explicit modeling of spatial position, ELLA explicitly models spatial position without making comparisons across treatment conditions. Specifically, ELLA models gene counts as a linear combination of beta distributions, each beta distribution in the basis describing a kernel count distribution. Like DESeq2, ELLA handles random variation via regression; specifically, it uses L1 regression with a single penalty factor. By default this factor is set to zero, but through *ad hoc* testing we determined that a value of 0.2 provides good control for FPR and FDR.

The main challenge in applying ELLA to attractor simulations is that it’s intended to model gene expression within single cells along the radial axis out from the nucleus. ELLA requires input data to include not only transcript coordinates, but coordinates for cell boundaries and cell center. By construction, attractor simulations control spatial distribution and effects on it with respect to the orthogonal axes of Cartesian coordinates. Thus, there is no point $\langle x, x^\bot \rangle$ within the simulated patch such that the count distribution along the radial axis out from that point is controlled by the simulation settings. To work around this problem, we transformed the coordinates $\langle x, x^\bot \rangle$ of attractor simulations before giving them to ELLA in such a way that the count distribution along the original axis $x$ was preserved along the radial axis out from the patch center (Fig. 3B, right):


(23) \begin{align*}
x^\prime &= x\cos ((x^\bot /\mathrm{max}(x^\bot ))2\pi ) \\{x^\bot }^\prime &= x\sin ((x^\bot /\mathrm{max}(x^\bot ))2\pi )
\end{align*}

To compensate for radial dilution, we also multiplied the count at each point by its radial distance and normalized.

ELLA was run on the transformed attractor simulations with all default settings, except for the modified L1 factor and slight tweaks to the minimum learning rate and maximum iterations to speed up fits. Adjusted $p$-values were taken from ELLA’s output and used to quantify FPR, FDR, and power for SVG testing, using the same definitions used for wispack and DESeq2.

### Cortex data

To demonstrate utility, we used a WSP model to test for affects of age and laterality (hemisphere) on the expression of six laminar-specific genes in mouse somatosensory cortex (Table 1). For this test we collected and preprocessed our own ST data.

#### Tissue collection and MERFISH sample preparation

Male CBA/CaJ mice aged postnatal (P) day 12 and P18 were used in accordance with the National Institute of Health guidelines, as approved by the Washington University in Saint Louis Institutional Animal Care and Use Committee. We had $n=2$ for each age, for four mice total. Mice were anesthetized with CO$_2$ and rapidly decapitated. The brains were harvested, placed in prechilled Optimal Cutting Temperature (Fisher) embedding medium, flash-frozen in 2-methylbutane on dry ice, and stored at $-80$°C until cryosectioning. Samples were prepared according to Vizgen’s modified formalin fixed-paraffin-embedded (FFPE) protocol for fresh frozen tissue. On the day of cryosectioning, frozen tissue blocks were allowed to equilibrate at $-20$°C in a cryostat (Leica CM1860 UV) for at least one hour prior to slicing horizontal, 14 µm thick sections. Slices were collected on MERSCOPE slides, and sections were allowed to adhere to the slides at $-20$°C for at least 30 min before being fixed with warmed 4% paraformaldehyde (PFA) in nuclease-free 1× PBS for 60 min at 37°C. The samples were then washed in nuclease-free 1× PBS $3\times$ (5 min each wash) and air dried in a covered slice rack to ensure complete tissue adhesion. The slices were then incubated in 70% ethanol at 4°C overnight for permeabilization.

#### Anchoring, gel embedding, and tissue clearing

The following day, sections were washed with conditioning buffer $3\times$[PN 20300116, $2\times$ at room temperature (RT) for 1 min, $1\times$ at 37°C for 30 min]. Sections were then treated with 100 µl of Pre-Anchoring Conditioning Buffer [100 µl of Conditioning Buffer (PN 20300116), 5 µl of Pre-Anchoring Activator (PN 20300113), and 5 µl of RNase Inhibitor (New England BioLabs)] for 2 h at RT followed by washes in Sample Prep Wash Buffer (PN 20300001, $1\times$ at RT for 5 min) and Formamide Buffer (PN 20300002, $1\times$ at 37°C for 30 min). After the formamide wash, the RNA was anchored to the tissue by incubating samples in Anchoring Buffer (PN20300117) for $\approx 16$ h at 37°C. Samples were then washed with Formamide Buffer ($1\times$ at 47°C for 15 min) and Sample Prep Wash Buffer ($1\times$ at RT for 2 min), gel embedded (50 µl), and cleared (incubated at 47°C for $< 24$ h). The gel embedding solution contained 5 ml of Gel Embedding Premix (PN 20300118), 25 µl of 10% ammonium persulfate, and 2.5 µl of N,N,N$^\prime$,N$^\prime$-tetramethylethylenediamine. Gel coverslips (PN 30200004) were cleaned with RNAseZap and 70% ethanol before 100 µl of Gel Slick solution was added. We allowed 15 min for the coverslips to air dry thereafter. Clearing solution contained 50 µl of Proteinase K (New England BioLabs) and 5 ml of Clearing Premix (PN 20300114).

#### Probe hybridization and MERSCOPE imaging

Tissue was then washed with Sample Prep Wash Buffer ($3\times$ at RT for 5 min) and Formamide Buffer ($1\times$ at 37°C for 30 min) before hybridizing with our custom 721-gene MERSCOPE gene panel (100 µl) and incubated at 37°C for 36–48 h after clearing. After probe hybridization finished, samples were washed with formamide buffer ($2\times$ at 47°C for 30 min), Sample Prep Wash Buffer ($2\times$ at RT for 5 min), and stained for 4$^\prime$,6-diamidino-2-phenylindole (DAPI) and poly-thymidine (PolyT) (at RT for 15 min on a rocker). The samples were washed with formamide buffer (at RT for 15 min on a rocker), Sample Prep Wash Buffer ($1\times$ at RT for 5 min), and loaded into the MERSCOPE for imaging.

#### Data preprocessing

Raw MERFISH spots were decoded using the integrated MERSCOPE software (v233). Cell segmentation on the MERFISH output data was done using the Cellpose 2.0 algorithm [57] on the DAPI and PolyT signal of the median plane of imaging (4 out of 7), utilizing the Vizgen Post-processing Tool (github.com/Vizgen/vizgen-postprocessing). The segmented cell boundaries of the median imaging plane were then propagated to all other layers. A set of filtering parameters were applied to the detected cells to remove false positives and badly segmented cells. First, all cells with volume inside the population’s first and last 2% quantile were removed. For each cell that passed the volume filter, the maximum count of the blank probes (98 total blanks) was selected as the false detection threshold, and genes that have their detected count under this threshold were removed from the cell. Cell transcripts were then normalized to the maximum cell volume of the population, assuming a linear scale, before removing cells with the normalized total transcript count in the first or last 3% quantile or with <30 unique genes (excluding blanks). Lastly, cells that passed the filters had their transcript data reset to the original non-normalized counts for downstream analysis.

**Figure 4.**
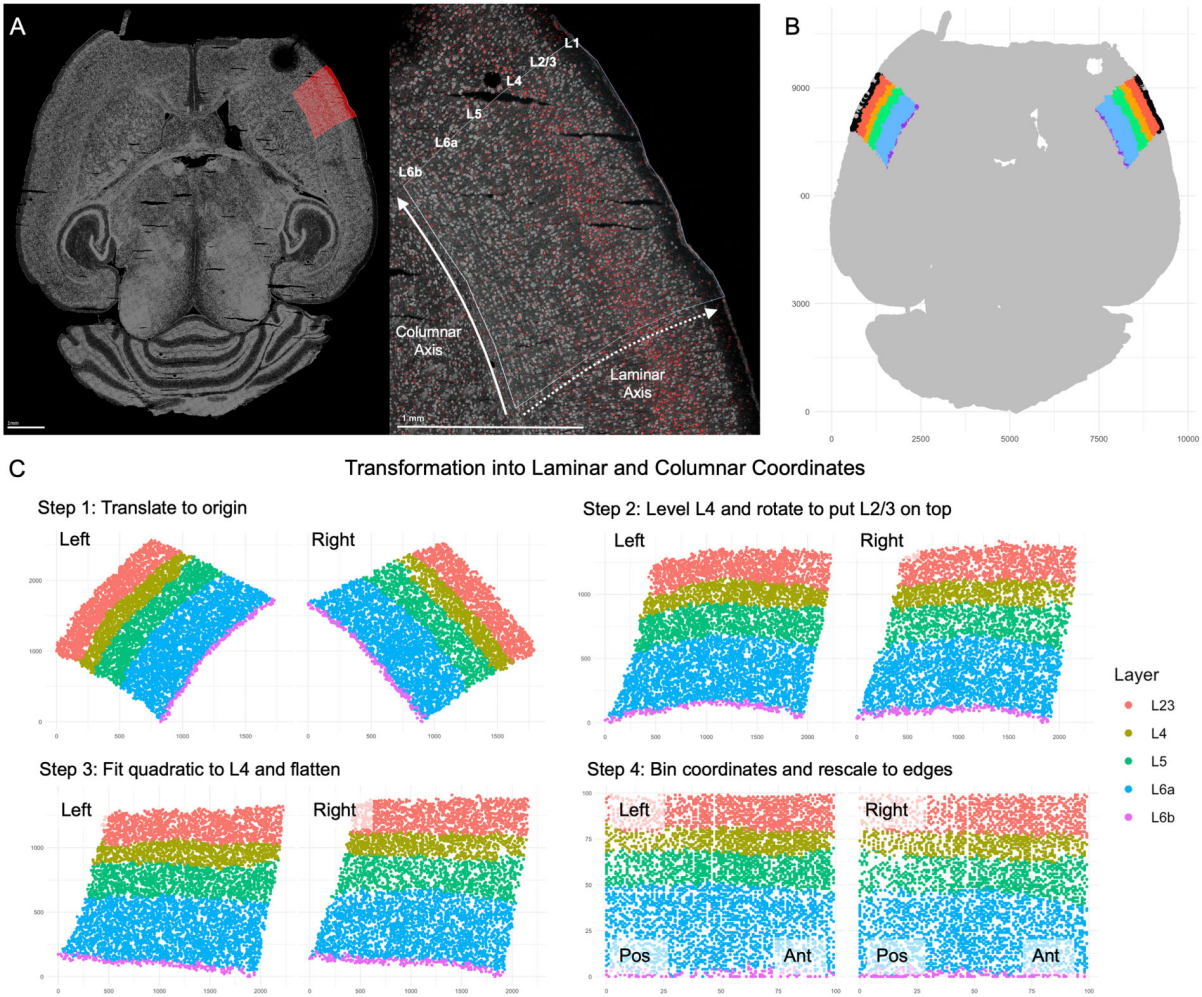
(**A**) Horizontal slice from a P18 male WT mouse used in the analysis (mouse 4). Both visualizations (with black background) produced via rendering of the transcripts observed on the MERFISH run. Left: Entire slice, right primary somatosensory cortex highlighted in red. The hole above highlighted area is a registration mark. Right: The same ROI, annotated with arrows showing the laminar and columnar axes with layers labeled. Red dots are ROR$\beta$ transcripts observed on the MERFISH run. Note the higher transcript density in L4. (**B**) The same slice and mouse, shown in post-registration cellpose coordinates with ROI and cortical layers colored. (**C**) Transformation from post-registration cellpose coordinates (same slice and mouse) into binned laminar and columnar coordinates. Note that the displayed coordinate axes may not represent the true origin for the step, as each step often involved several trivial linear transformations to put the coordinates into a more convenient form. All steps shown with the left and right tissue patches equivalently positioned in the positive coordinate quadrant. Credit: Components of this figure appear in the online documentation (michaelbarkasi.github.io/wispack/).

Tissues were manually aligned to the Common Coordinates Framework v3 (CCFv3) [58] by matching the DAPI and PolyT histology stains to the reference atlas (25-µm resolution) in QuickNII [59]. Non-linear refinements to the registration were done using VisuAlign (nitrc.org/projects/visualign) to get the final indexed mask of CCFv3 labels for the registered tissue. The label mask was then fitted to filtered cells’ coordinates to transfer the CCFv3 labels to corresponding cell identities. Only cells labeled as Primary Somatosensory (S1, regardless of subregion) were used in downstream analysis (Fig. 4A and B).

#### Transformation into laminar coordinates

After registration, we had a list of cells annotated with (i) coordinates $\langle \mathbf {x},\mathbf {y}\rangle$ from cellpose giving the position of the cell’s center, (ii) layer identity and ROI from the CCFv3, and (iii) for each gene in our panel, a raw (i.e. non-normalized) count of transcripts in that cell. So, each transcript was not assigned its own actual observed coordinate, but the coordinate of its containing cell. The final preprocessing step was to transform the cellpose coordinates into a coordinate system defined by functionally meaningful axes. As we were interested in the laminar axis, we performed a series of geometric transforms which resulted in one axis perpendicular to the laminar boundaries identified by the CCFv3 registration and a second orthogonal axis presumably perpendicular to the cortical columns of S1 (Fig. 4). These transformations were performed individually on each hemisphere of each mouse. Note that we excluded L1, as it contains very few cell bodies (Fig. 4). Each transformation was designed (and manually checked) to respect orientations, so that the origin was the deepest point perpendicular to the cortical surface (i.e. the deepest point in L6b) and also the most posterior point. Hence, the columnar axis ran from posterior to anterior and the laminar axis ran from L6b to L2/3.

As shown in Fig. 4C, there were four steps to the coordinate transformation. (Step 1) The tissue patch was translated to the origin by subtracting the mean point of L5 along both axes. (Step 2) The tissue patch was leveled by fitting a linear model $\mathbf {y}=\mathbf {m}\mathbf {x}+\mathbf {b}$ to the points of L4, then applying a matrix transform performing a rotation of $\arctan (\mathbf {m})$ radians (or its negative for negative slope). Coordinates were reflected across the horizontal axis, if necessary, to ensure L2/3 was on top. (Step 3) The curvature of the layers was flattened by fitting a quadratic model $\mathbf {y}=-\mathbf {c}(\mathbf {x}-\mathbf {a})^2+\mathbf {b}$ to L4 using nonlinear least squares, then each point was transformed via $\langle {}\mathbf {x},\mathbf {y}\rangle \mapsto \langle {}\mathbf {x},\mathbf {c}(\mathbf {x}-\mathbf {a})^2+\mathbf {b}\rangle$. (Step 4) The coordinates of both axes were binned so that the deepest, posterior most point was in bin $\langle 0,0\rangle$ and the most superficial, anterior point was in $\langle b_{p},b_{p}\rangle$, then coordinates were stretched to fill the bins by, for each bin $\mathbf {x}$, finding the max bin $\mathbf {y}_{\mathrm{max}}$ holding a cell and rescaling the $\mathbf {y}$ component of all points in $\mathbf {x}$ with $\mathbf {y}\ge b_{p}/2$ so that $b_{p}/2\mapsto b_{p}/2$ and $\mathbf {y}_{\mathrm{max}}\mapsto b_{p}-|{\epsilon }|_{\mathrm{abs}}$, for some small jitter $\epsilon$. This rescaling was weighted by each cell’s distance from the midpoint $b_{p}/2$, so that points near the midpoint only received a small fraction of the rescaling while those at the max receive all of the rescaling. An analogous rescaling was performed for points in $\mathbf {x}$ such that $\mathbf {y}< b_{p}/2$ to pull $\mathbf {y}_{\mathrm{min}}$ down to zero (plus some positive jitter) and two analogous rescalings were done for the $\mathbf {y}$ bins so that $\mathbf {x}_{\mathrm{max}}$ was pulled over to $b_{p}$ and $\mathbf {x}_{\mathrm{min}}$ was pulled back to zero. The exact algorithm for these steps can be found in the wispack code. After performing this transformation, the final binned coordinates $x$ used to fit the WSP model were obtained by simply dropping the $\mathbf {x}$ coordinate (i.e., the coordinate orthogonal to the laminar axis) so that $x=\mathbf {y}$.

#### Cell typing

We used MapMyCells (portal.brain-map.org/atlases-and-data/bkp/mapmycells, RRID:SCR_024672) from the Allen Institute to type cells based on the counts from the entire 721 gene panel. From the Allen cell-type labels, we identified and relabeled all cells with a label including “Glut” or “GABA,” for glutamatergic and GABAergic cells, respectively. We excluded all other cells from the data set. The simplified and reduced cell types were used when fitting a WSP model to the data.

### Liver data

As a second demonstration of utility, and to demonstrate the applicability of WSP models in multiple biological contexts, we used a WSP model to reproduce, for six genes $g$ (Table 2), the analysis done by Droin *et al*. [14]. They analyzed the effects of circadian rhythm on radial zonation in liver lobules with a bespoke linear mixed-effects model. Their model represented spatial variation with second-degree Legendre polynomials and represented temporal rhythm with harmonic (sine and cosine) terms.

We used the data provided by Droin *et al*. [14], consisting of bulk-sequenced transcript counts-by-cell, for a single cell type (hepatocytes), from ten mice collected at four zeitgeber time (ZT) points: ZT0 h, ZT6 h, ZT12 h, and ZT18 h. Droin *et al*. [14] divided lobules into eight zones (1 being most central, 8 most portal), assigned a probability of zone membership to each cell based on marker genes, multiplied that probability by normalized count values for each gene, and log-transformed this value. The result was extrapolated, normalized, and log-transformed gene counts attached to extrapolated zonal coordinates.

As WSP models require integer counts, we did not follow this reconstruction. Instead, for each cell with normalized count $y$ and probabilities $\mathrm{P}_x$ for being in zone $x=1,\ldots ,8$, we drew counts from a Poisson distribution:


(25) \begin{eqnarray*}
y_x\sim \mathrm{Pois}(10^3y)
\end{eqnarray*}

The multiplication by a thousand is to denormalize $y$ to a realistic rate. This left us with eight integer counts $y_x$ and probabilities $\mathrm{P}_x$ for each cell in the original data set. Using binary values $\mathrm{b}_x\in \lbrace 0,1\rbrace$ drawn from binomial distributions:


(26) \begin{eqnarray*}
\mathrm{b}_x\sim \mathrm{Binom}(1,\mathrm{P}_x)
\end{eqnarray*}

these counts and probabilities were turned into pseudo-cells, one for each zone, with counts $y_x\mathrm{b}_x$.

## Results

### Benchmarking

Bespoke simulations based on real seed data (MERFISH data from mouse brain slices, Yao *et al*. [55]) were used to assess FPR, FDR, and statistical power for wispack and two other transcript analysis tools, DESeq2 [24] and ELLA [31]. These simulations, dubbed *attractor simulations*, allowed for precise control of (i) the spatial distribution within a condition, (ii) an effect on that distribution between conditions, and (iii) random variation between replicates. A full description of the simulations and how wispack, DESeq2, and ELLA were applied to them is provided above.

Run on 250 attractor simulations of four genes each (for one thousand total test points), both wispack and ELLA hit a FDR under the intended $\alpha =0.05$ for their respective hypothesis testing domains (FSE testing for wispack, SVG testing for ELLA). Wispack and ELLA also had comparable power, around 0.4. Wispack’s estimates for the rate effects were highly correlated with the stipulated value (Pearson correlation coefficient of about 0.85 for $\beta _{r}$ and about 0.8 for $\rho _{r}$). DESeq2 did not hit the FDR target of $\alpha =0.05$, with the final value being around 0.4, although power in FSE testing was higher, at around 0.8. Full results given in Table 4.

**Table 4.** Benchmarking results, including “corr” (Pearson correlation coefficient) for estimated versus stipulated fixed ($\beta _{r}$) and random ($\rho _{r}$) effect on expression rate, false-positive rate (FPR), false-discovery rate (FDR), and power, as well as mean time to run each model on a simulation

	DESeq2	ELLA	wispack
corr, $\beta _{r}$	0.234		0.855
corr, $\rho _{r}$			0.794
hypothesis test	FSE	SVG	FSE
FPR	0.243	0.023	0.003
FDR	0.414	0.036	0.016
power	0.818	0.401	0.426
mean fit time (s)	1.912	27.504	425.564

Also of note is the mean time to fit each model to each simulation. There is an order-of-magnitude increase in compute time going from DESeq2 to ELLA, and another order-of-magnitude increase going from ELLA to wispack. The jump from DESeq2 to ELLA is due to the modeling itself: DESeq2 fits a generalized linear model (GLM) to group means, ELLA fits a model to individual data points. The jump from ELLA to wispack is due to the method of hypothesis testing. Wispack also performs individual model fits in 20–30 s. However, to estimate CIs and $p$-values wispack has to fit models to many bootstrap resamples. In this benchmark test, we used a hundred cores in parallel to fit a thousand resamples per simulation, leading to the ten-fold compute time increase.

### Cortex data

Coordinates along the laminar axis of each tissue sample were divided into $b_{p}=100$ bins. Mouse number was used as the levels $k$ of the random-effect factor $\nu$. There were five random-effect levels all together, the four mice plus the pseudo reference level. Hemisphere and age were the two fixed-effect factors analyzed, i.e., $\xi =\langle \xi _1=\mathrm{hemisphere},\xi _2=\mathrm{age}\rangle$, with the left hemisphere and P12 being used as reference levels and the right hemisphere and P18 treatment levels. Thus, there were four treatment interaction levels $w$: (i) baseline (left, P12), (ii) hemisphere (right, P12), (iii) age (left, P18), and (iv) the age-hemisphere interaction (right, P18).

Six genes ($g$), two cell types (${c}$), five random-effect levels (mice plus extrapolated reference, $\nu$), four treatment interaction levels (hemisphere $\times$ age, $w$), and 100 position bins ($x$) gave 24,000 potential observations (rows of data) to fit. The count $y$ for each row was gotten by summing the transcript count of that row’s gene $g$ from all cells annotated by the values ${c}$, $x$, $\nu$, and $w$ of that row. A similar summing of counts is performed by ELLA [31]; summing does not affect model fit because the sum of two Poisson-distributed variables is itself Poisson-distributed. As each mouse could only be observed at one age, 9,600 of these rows were empty (half of all rows not including the pseudo random-effect reference level), with an addition 432 empty rows due the low number of GABAergic cells (not all bins $x$ for all combinations $g\times \nu \times w$ had at least one GABAergic cell), although the WSP model still made predictions for these empty rows.

**Figure 5.**
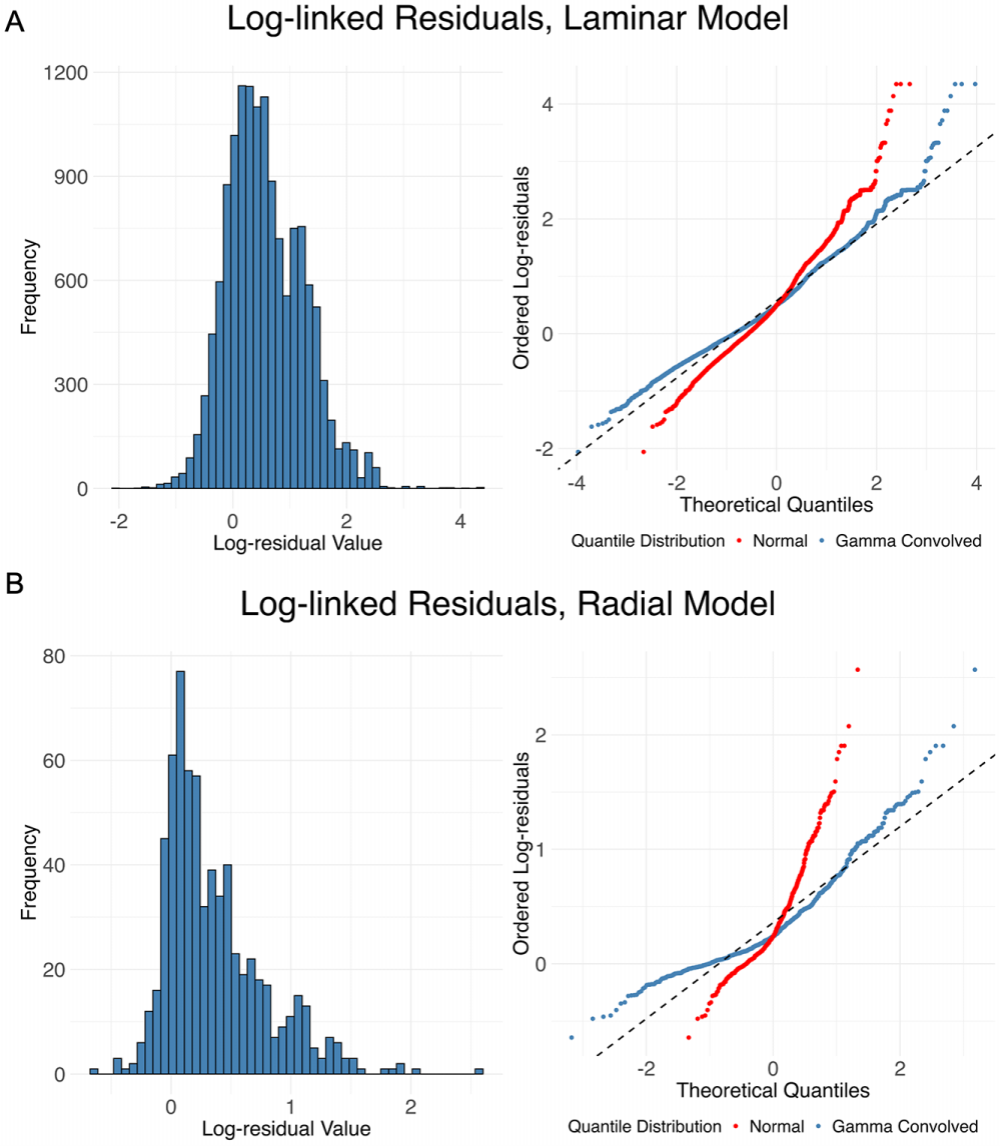
Histograms (left) and *Q*–*Q* plots (right) for the residuals from the L-BFGS WSP model fit, for both the model of (**A**) the laminar axis of the cortex data and (**B**) the radial axis of the liver data. The *Q–Q* plots show both the theoretical quantiles from a normal distribution (red) and the theoretical quantiles derived from a normal distribution convolved with the gamma distribution used to fit the data (blue, equation A.16). The way the blue points are closer to the dashed black line than the red points shows that the residuals are more normal when the over-dispersion from the gamma distribution is taken into consideration. That is, modeling the over-dispersion with a gamma kernel notably improves model fit.

Model degree $d$ was estimated for each gene $g$ with a likelihood-ratio outlier (LRO) change-point detection algorithm (Appendix 1.1), resulting in a WSP model with 612 parameters $\Phi=\langle \beta ,\rho \rangle$. The posterior distribution $\mathrm{P}(\beta ,\rho \, |\, y,\zeta )$ of the WSP model parameters was estimated with a 10,000 resample bootstrap, which was used to compute CIs and $p$-values (Appendix 2). Total time to complete the bootstraps was 205 min, averaging about 1.23 s per bootstrap, implying about 24.6 seconds per thread. L-BFGS converged (tolerance of $10^{-7}$) on 9,990 bootstraps (mean of 200 iterations, max of 335), giving 9,990 samples of the parameter vector $\Phi$, plus the original parameters fit to the full data. The residuals from fitting a WSP model to the cortex data are shown in Fig. 5A as a histogram and *Q-Q* plot. Full numeric results for the parameter estimates are provided in Supplementary Table S1.

**Figure 6.**
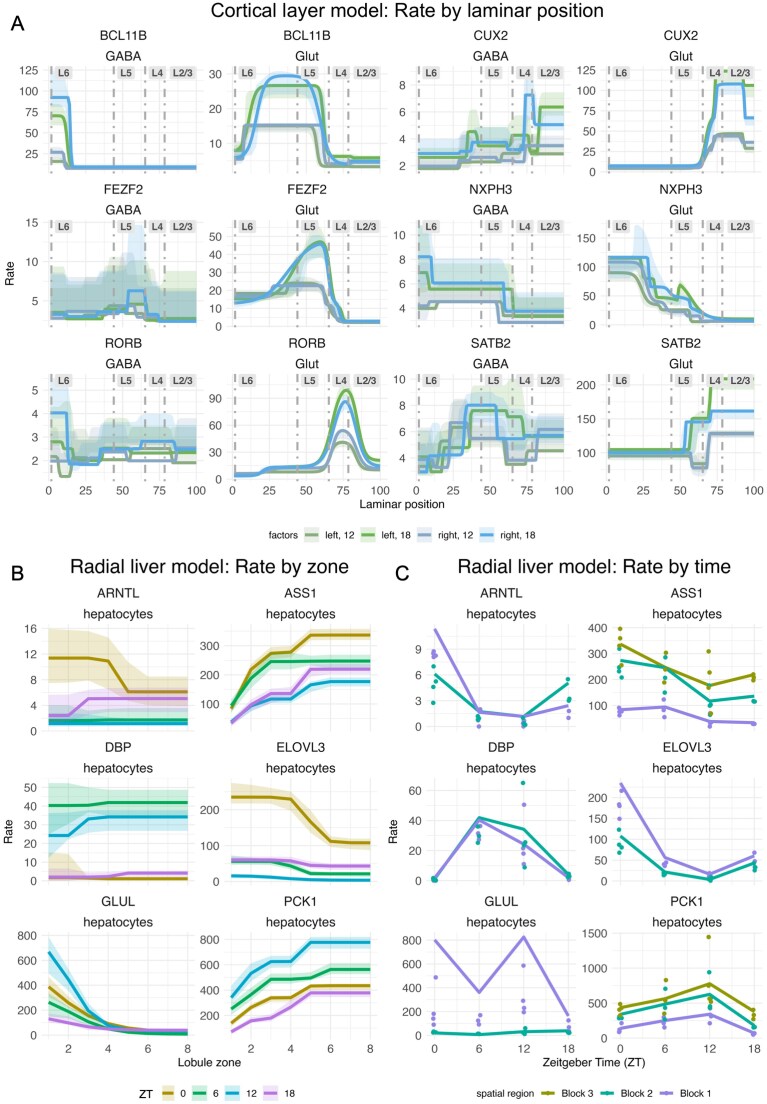
Results of fitting a WSP model in the two demonstration cases. (**A**) Results showing predicted rate versus spatial position from the WSP model fit to the MERFISH data on somatosensory cortex. (**B**) Results showing predicted rate versus spatial position from the WSP model fit to the liver data with extrapolated zonal coordinates. (**C**) Results showing predicted block rate versus time (ZT) from the same WSP model fit to the liver data.

**Figure 7.**
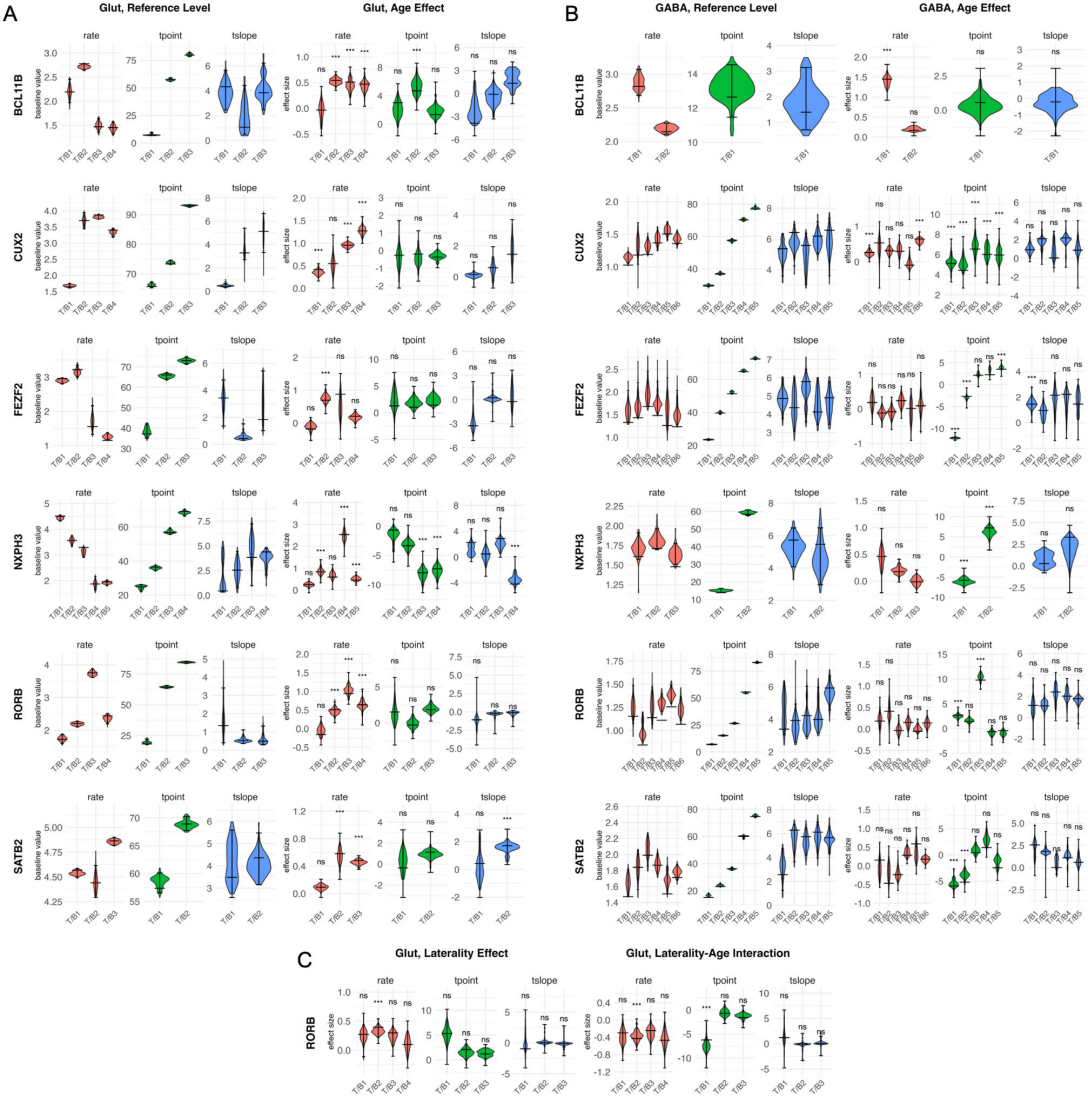
Selected parameters from the WSP model of the laminar axis of the cortex data. All plot facets show violin-style distribution plots of the parameters extracted from the bootstrap resamples. Parameter types rate $r$, transition point location $p$, and transition point slope scalar $s$ are labeled above the plot facets as “rate,” “tpoint,” and “tslope,” respectively. The mid horizontal bar represents the parameter value estimated from the model fit to the original data, while top and bottom horizontal bars show the 95%-CI range. The horizontal axis labels “T/B1,” “T/B2,” etc. stand for “transition-point/block” and indicate the transition point (for transition point location and slope scalar) or block (for rate) of the parameter. Significance marks for the effect parameters are based on adjusted $p$-values and are defined $\mathrm{ns}=p\ge 0.05$, $\mathrm{*}=p< 0.05$, $\mathrm{**}=p< 0.01$, and $\mathrm{***}=p< 0.001$. (**A**) Baseline (reference level) parameters (left) and age-effect (the shift from P12 to P18) parameters (right) for all analyzed genes in glutamatergic cells. (**B**) Same as panel (A), but for GABAergic cells. (**C**) Laterality effect (the shift from left-to-right hemisphere) and laterality-age interaction (the shift from left-to-right hemisphere when at age P18) for ROR$\beta$ in glutamatergic cells.

The estimates of transition points $p$ between expression rates made by the WSP model were generally in the expected locations, based on the laminar boundaries obtained independently from the CCFv3 registration and the expected layers listed in Table 1, as seen in the plots in Fig. 6A. Quantitative parameter estimates largely confirmed this picture for glutamatergic cells (Fig. 7A), but showed a more unclear picture for GABAergic cells (Fig. 7B).

Specifically, for glutamatergic cells, SATB2 is upregulated in L2/3 (block 3 in the model), as expected (95%-CI for log-linked kernel rate of the reference level of $\langle 4.819, 4.895\rangle$ for block 3, versus $\langle 4.500, 4.583\rangle$ and $\langle 4.294, 4.613\rangle$ for blocks 1 and 2). CUX2 is upregulated in L2/3 and L4 (blocks 2–4 in the model), as expected, for glutametergic cells (95%-CIs for log-linked kernel rate of the reference level of $\langle 3.442, 3.943\rangle$, $\langle 3.739, 3.874\rangle$, and $\langle 3.184, 3.457\rangle$ for blocks 2–4, versus $\langle 1.608, 1.742\rangle$ for block 1). Also as expected, ROR$\beta$ shows a strong, sharp spike at L4 (block 3 in the model) in glutamatergic cells (95%-CI for log-linked kernel rate of the reference level of $\langle 3.608, 3.872\rangle$ for block 3, versus $\langle 1.592, 1.863\rangle$, $\langle 2.106, 2.266\rangle$, and $\langle 2.195, 2.505\rangle$ for blocks 1, 2, and 4). FEZF2 showed upregulation in L5 (block 2 in the model) for glutamatergic cells, as expected; while the upregulation is minor at the reference level (95%-CI for log-linked kernel rate of $\langle 3.000, 3.355\rangle$ for block 2, versus $\langle 2.816, 2.979\rangle$ for block 1 and $\langle 1.329, 2.161\rangle$ and $\langle 1.169, 1.371\rangle$ for blocks 3 and 4), there was a significant age effect for L5 ($\beta =0.693$ for block 2, $p< 0.001$) lacking for all other regions. Fitting the expectation for upregulation in L5 and L6, BCL11B showed upregulation in glutamatergic cells in L6a and L5 (block 2 in the model, which had a 95%-CI for log-linked kernel rate of $\langle 2.650, 2.780\rangle$, versus $\langle 1.975, 2.455\rangle$, $\langle 1.361, 1.641\rangle$, and $\langle 1.316, 1.571\rangle$ for blocks 1, 3, and 4). Finally, NXPH3 was upregulated in L6 (blocks 1–3 in the model), as expected, in glutamatergic cells (95%-CIs for log-linked kernel rate of the reference level of $\langle 4.332, 4.556\rangle$, $\langle 3.362, 3.743\rangle$, and $\langle 2.902, 3.310\rangle$ for blocks 1–3, versus $\langle 1.643, 2.157\rangle$ and $\langle 1.809, 2.011\rangle$ for blocks 4 and 5).

For GABAergic cells, SATB2, ROR$\beta$, FEZF2, and NXPH3 showed relatively consistent expression levels through all cortical layers (Fig. 7B) with no significant rate effects for GABAergic cells (Supplementary Table S1). For GABAergic cells, CUX2 expression was consistent across the cortical layers at the reference age of P12, although there was a significant increase in the superficial range of L2/3 from age (block 6 in the model, $\beta = 0.639$, $p< 0.001$) that isn’t matched in the deeper regions. The deepest region of cortex, block 1, did see a significant increase in CUX2 with age ($\beta = 0.260$, $p< 0.001$). BCL11B is expected to be upregulated in L5 and L6, and GABAergic cells showed upregulation in L6b (block 1 in the model, which had a 95%-CI for log-linked kernel rate of $\langle 2.683, 3.070\rangle$ with an age effect of $\beta = 1.448$ with $p< 0.001$, versus $\langle 2.105, 2.270\rangle$ and no age effect on rate for block 2).

Consistent with our hypothesis, we found effects of both age and laterality on ROR$\beta$ expression in glutamatergic cells (Fig. 7A and C), including two potential FSEs of note. (i) The WSP model showed an increase in ROR$\beta$ expression (kernel rate) from P12 to P18 (the two ages for which we have data) across all but the deep part of L6 along the laminar axis, with the largest natural-log fold change in block 3, the block most closely aligned with L4 ($\beta =0.940$, $p< 0.001$). (ii) The WSP model showed a significant effect of laterality in L5 and the superficial part of L6, both at the reference age of P12 and as an interaction with the treatment age of P18. Interestingly, these effects went in opposite directions: ROR$\beta$ expression (kernel rate) in block 2 in the right hemisphere was increased (relative to the left) at P12 ($\beta =0.392$, $p< 0.001$), but decreased (relative to the left) at P18 ($\beta =-0.428$, $p< 0.001$). Along with these effects on rate, the first transition point, in the middle of L6, moves deeper with age in the right hemisphere relative to the left ($\beta =-6.229$, $p< 0.001$). This effect is equivalent to about $6\%$ the thickness of the cortex and suggests that, between P12 and P18, the area of active ROR$\beta$ expression in L6 expands downward toward the white matter in the right hemisphere.

In GABAergic cells, there were effects of age and laterality on ROR$\beta$ transition point locations and transition point slope scalars, but not on rate parameters. As GABAergic cells lacked the characteristic upregulation of ROR$\beta$ in L4 (and showed little spatial variation in general), these results are therefore outside the main scope of our demonstration hypothesis.

### Liver data

We fit a WSP model to the counts reconstructed from the Droin *et al*. [14] data using a single time-series fixed effect (ZT, yielding four treatment levels ZT0, ZT6, ZT12, ZT18 with ZT0 as reference level) and a random-effect level for each of the ten mice from the original data. Each radial zone was taken as a bin coordinate, resulting in a spatial axis of eight points. The central-most point was $x=1$, the portal-most point was $x=8$. Unlike the cortex data which divided cells into two types, a single cell type (hepatocytes) was used in this model.

Six genes ($g$), one cell type (${c}$), eleven random-effect levels (mice plus extrapolated reference, $\nu$), four treatment interaction levels (ZTs, $w$), and eight position bins ($x$) gave 2,112 potential observations (rows of data) to fit. As with the cortex data, the count $y$ for each row was gotten by summing the transcript count of that row’s gene $g$ from all cells annotated by the values $x$, $\nu$, and $w$ of that row. Also as with the cortex data, having samples for each mouse at only one time point meant that 1,440 of the observation rows were empty, although the WSP model still made predictions for these rows. Only $48\cdot 2\cdot (3+4) = 672$ of the 2,112 rows were filled because four time points and eleven random-effect levels implies 48 potential rows per time point per random-effect level. Two of the four time points had data from two replicates and the other two had data from three (the extrapolated reference level gave data for all time points).

As with the WSP model for the cortex data, LRO change-point detection was used to estimate model degree $d$ (Appendix 1.1) and a 10,000 resample bootstrap was used to compute $p$-values and 95%-CIs (Appendix 2). The resulting model had 300 parameters. Total time to complete the bootstraps was 76 min, averaging 0.46 s per bootstrap, implying about 9.1 s per thread. L-BFGS converged (tolerance of $10^{-7}$) on all 10,000 bootstraps (mean of 150 iterations, max of 259). Residuals from the model fit are shown in Fig. 5B as a histogram and *Q–Q* plot. Full numeric results for the parameter estimates are provided in Supplementary Table S2. Plots visualizing the model fits plus 95%-CIs are provided in Fig. 6B. Plots visualizing the rate parameter for each gene as a function of ZT are provided in Fig. 6C.

The WSP model of the radial liver data reproduced the general patterns uncovered by Droin *et al*. [14] (Fig. 6B and C). These patterns were largely consistent with those listed in Table 2, as seen in the plots in Fig. 6B and C. However, matching the results from Droin *et al*. [14], these patterns exhibited more complexity than suggested by Table 2.

**Figure 8.**
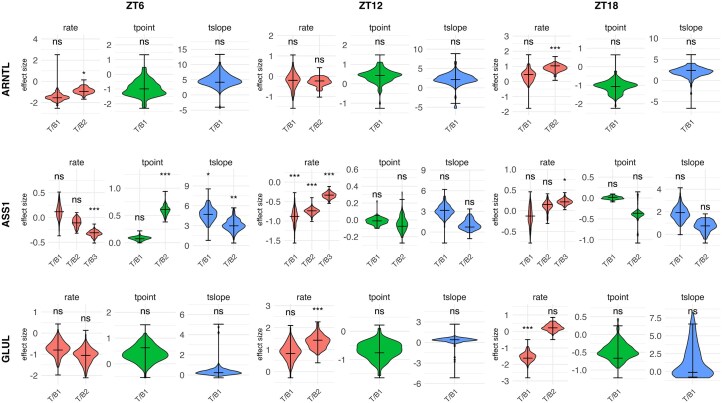
Selected model parameters for selected genes from the WSP model model of the radial axis of the liver lobule data. The baseline (reference level) parameters for ZT0 are left off; what’s shown are the fixed-effect parameters for the three treatment conditions (ZT6, ZT12, and ZT18). All plot formatting is as explained in Fig. 7.

The two zonated genes, GLUL and ASS1, showed the expected zonation (central and portal, respectively), but the time-series plots (Fig. 6C) and parameter estimates (Fig. 8) revealed change over ZT as well. The portal region of ASS1 (block 3 in the model) showed a drop in rate between ZT0 and ZT6 ($\beta = -0.307$, $p< 0.001$), a drop in rate between ZT6 and ZT12 ($\beta = -0.334$, $p< 0.001$), and an uptick in rate between ZT12 and ZT18 ($\beta = 0.215$, $p=0.044$). The central region of GLUL (block 1 in the model) appears in the time-series plot to have an oscillation in expression with peaks at ZT0 and ZT12, although the only significant effect found was the drop from ZT12 to ZT18 ($\beta = -1.618$, $p< 0.001$); curiously, the portal region (block 2) had a significant increase in expression from ZT6 to ZT12 ($\beta = 1.432$, $p< 0.001$).

The two rhythmic genes, ARNTL and DBP, seemed to show less spatial variation than the zonated genes showed temporal variation, but the time-series plot for ARNTL suggested that, from ZT0 to ZT6, the central region (block 1) starts with higher expression and drops more than the portal region (block 2) and that, from ZT12 to ZT18, expression in the portal region increases more than in the central region. However, the large drop in expression in the central region (block 1) from ZT0 to ZT6 was not significant within the model ($\beta = -1.542$, $p= 0.127$), while the smaller drop in the portal region (block 2) was significant ($\beta = -0.957$, $p=0.045$). From ZT12 to ZT18, the portal region (block 2) showed a large increase ($\beta = 1.030$, $p< 0.001$), while the central region (block 1) showed a much smaller and insignificant increase ($\beta = 0.459$, $p> 1$ after adjustment). This difference in effects of ZT on the central vs portal regions is itself interesting, as it suggests that the effects of the temporal rhythm of ARNTL depends on the spatial region.

## Discussion

Despite a growing number of new tools for analyzing ST data, before WSP models there were none available for answering seemingly simple questions such as whether ROR$\beta$ expression rate is lower in the right hemisphere compared to the left. While technically possible, tackling such a question would have required a bespoke model, similar to how Droin *et al*. [14] used a bespoke GLM to model zonation and circadian rhythm in liver-lobule gene expression, or bespoke data preprocessing with a high risk of biasing the answer, e.g. by manually specifying the intracortical boundaries of ROR$\beta$ expression. WSP models provide a powerful modeling framework applicable to different contexts and simplify spatial preprocessing to a basic step—aligning spatial coordinates with an axis of interest—with less risk of biasing the answer. WSP models provide this framework via a plausible mathematical model of the spatial distribution of gene transcription rates and enable this less-biased hypothesis testing by offloading the estimation of key spatial parameters (especially transition points between expression rates) to a data-driven, likelihood-based regression. Thus, WSP models provide a way to test hypotheses about FSEs, i.e. about the factors which have functionally relevant effects on the spatial distribution of gene expression. By enabling testing of FSEs, WSP models provide a powerful new tool for uncovering the genetic mechanisms behind biological and cognitive functioning.

### Biological applications

To demonstrate WSP models’ capability to test hypotheses concerning FSEs, we hypothesized that ROR$\beta$ expression in developing mice varies significantly with age and hemisphere. We hypothesized an effect from age based on prior work showing two key developmental trends in mice [10, 11]: (i) barrel distinctness and ROR$\beta$ expression rise in parallel from birth to P7, and (ii) barrel distinctness then wanes from P10 to P30, with a significant decrease by P20 in males. The first observation suggests a tight early correlation between ROR$\beta$ and the physical refinement of barrels, yet leaves open whether that relationship persists later, e.g. during the waning period observed after P10. Results from the WSP model, fit to data we collected from P12 and P18 male mice, provides a preliminary answer. Instead of the decline suggested by observations (i) and (ii), the WSP model showed that ROR$\beta$ increased further in glutamatergic cells at P18, compared to P12 (Fig. 7A), even as barrel distinctness presumably fell, revealing a decoupling of gene expression from the macroscopic barrel phenotype in late development. This late rise implies that ROR$\beta$’s role extends beyond initial barrel sharpening, underscoring WSP models’ power to pinpoint age-dependent shifts in gene–circuit relationships.

Laterality is essential for a variety of sensorimotor tasks [60], but when and how it emerges in cortex is still unclear. We recently showed circuit-level mechanisms that could drive left–right specialization in the mouse auditory cortex [40], yet molecular programs remain unresolved. Observations of laterality during development have also been observed in S1 of rats; at P20, total cortical area containing barrels was observed to be significantly greater on the left (and therefore smaller on the right) in males [41]. The WSP model fit to our data from P12 and P18 male mice uncovered molecular asymmetry in glutamatergic cells (Fig. 7C). Specifically, ROR$\beta$ expression in these cells increased from P12 to P18 in both hemispheres, but increased more in the left. This difference was significant in block 2 (corresponding to L6a and L5), but not in block 3 (corresponding to L4), although the general trend appeared across the entire laminar axis. Some adult mouse studies ($> $P60) do report no barrel-field laterality [61, 62], but those analyses may miss the transient window we and others observe in pups ($< $P20).

To demonstrate that WSP models apply beyond the case of gene expression along the laminar axis of the cortex, we recreated part of the analysis done by Droin *et al*. [14] on the functionally relevant radial axis of liver lobules. In this case, we showed how a WSP model can be used to model changes in the spatial distribution of gene expression over time. In addition to an entirely new biological context and the modeling of a time series, this case demonstrated that WSP models do not require high-resolution ST data and can be used with coarse-grain extrapolated spatial coordinates.

### Comparison with SVG testing

Recent statistical methods for ST data have focused on detecting spatially variable genes (SVGs), both within a single cell and across larger tissue samples [28, 29]. SVG detection methods include both artificial neural network classifiers and traditional regression models. Most regression approaches fit a model to transcript counts, although some fit to a proxy of spatial gene count distribution, such as diffusion time (sepal [63]) or interaction energy (BOOST-MI [64]). Many of the count-based regression approaches (e.g. SpatialDE [65], SPARK [66], SpatialDE2 [67], and SMASH [68]) predict gene expression rate (a log-linked raw transcript count or normalized transformation of it) with a GLM, under the assumption that observed counts are Poisson distributed, possibly also using an inner convolution or negative binomial to handle over-dispersion (equations 4 and 5). These approaches further assume that one of the terms in the model, either some set of covariates $X$ or coefficients $\beta$, comes from a multivariate normal distribution ($\mathrm{MVN}$). The idea is that if the covariance matrix, or *kernel*, of this distribution is a function of spatial position, then this spatial covariation indicates that the gene being tested is spatially variable.

The main difference between these spatial kernel-based SVG tests and WSP models is the latter’s emphasis on theory-driven hypothesis testing. While some SVG tests are explicitly formulated as tools for hypothesis testing, they face one or both of two limitations [29, 69]. The first limitation is that these tools only allow for testing the hypothesis that a gene is spatially variable. For example, SPARK [66] models expression rate $\Lambda$ as a linear function of position $x$ including a term $\mathbf {b}(x)\sim \mathrm{MVN}(0,\tau \mathbf {K}(x))$ assumed to come from a $\mathrm{MVN}$ centered on zero with covariance $\mathbf {K}(x)$ scaled by $\tau$. The symbols “$\tau$,” “$\mathbf {b}$,” and “$\mathbf {K}$” are their notation. The Satterthwaite method with score statistics is used to compute a $p$-value for the null hypothesis that $\tau =0$. While this procedure tests for non-zero spatial covariance (i.e., tests for SVGs), it does not allow for testing for effects on that spatial covariance.

SPARK also exemplifies the second limitation common to SVG tests (e.g. SpatialDE, MERINGUE, SpaGCN, and DESpace): it does not allow for testing for *between-group* effects [69]. For example, SPARK cannot be used to test whether a factor like cell-type, age, or gene-knockout affects the spatial-covariance scalar $\tau$, i.e., affects whether a gene is a SVG. SPARK is limited to *within-group* testing, i.e., to testing whether a gene as observed in one set of conditions (e.g., holding cell-type and age constant) is spatially variable. While between-group testing has been a staple of traditional differential expression tools such as DESeq2 and some recent SVG tools such as SPADE [69] have introduced it as well, the first limitation remains.

### Alternative tools for FSE testing

These limitations are largely intrinsic to the structure of common SVG methods, which essentially work by testing for non-zero spatial covariance (Fig. 1A). The focus on spatial covariance is a serious hurdle for integrating SVG tools with genomics databases such as GO [26] and KEGG [27], as even with the addition of between-group testing, effects on spatial covariance are not readily interpretable as log fold changes. Further, it is unclear how to extend these methods into tests for FSEs. As articulated in the difference between equations 2 and 3, testing for FSEs is most straightforward when there is an explicit parameterization of the spatial distribution—after all, a FSE is an effect on some parameter of the spatial distribution of a gene.

Consider ELLA [31], which instead of modeling spatial covariance models spatial distribution explicitly. Spatial distributions are modeled in ELLA as combinations of component kernel distributions $f$. So, in contrast to a WSP model, which allows for testing a hypothesis on measurable parameters, such as “factor $\xi$ moves transition points $p$ so that expression is upregulated across the entire region,” adaptations of ELLA (or any other kernel-based GLM) to FSE testing would be limited to testing hypotheses with forms like “factor $\xi$ has effect $\beta$ on kernel component $f$,” which would be challenging to interpret.

Three other recent tools for modeling ST data with potential for adaptation for FSE testing are SpaNorm [50], C-SIDE [70], and Niche-DE [71]. SpaNorm is a mixed-effects GLM that decomposes gene expression rate into two kinds of effects: those representing “biologically relevant smooth spatial variation” and those representing “smooth spatial variation related to (log) library size” [50, p. 10]. Both kinds of effects are modeled as linear coefficients on non-linear B-spline basis functions of spatial coordinates. SpaNorm outputs transcript counts that reflect only biologically relevant spatial variation, thereby factoring out random effects, and this output could be integrated with some other statistical model to estimate FSEs. However, SpaNorm will face a limitation similar to ELLA. SpaNorm is ultimately still a “kernel” based method which represents spatial variation with *ad hoc* nonlinear functions. Thus, like any kernel-based approach, it’s statistical power is only as good as the match between its kernels and the true underlying spatial distribution, and effects on kernel coefficients are difficult to interpret intuitively.

C-SIDE (cell type-specific inference of differential expression) is a powerful modeling framework based around a mixed effects GLM [70]. Like DESeq2, the model predicts log-linked expression rate and is intended for DE testing. As the name implies, this expression rate, observed within a given “pixel,” is assumed to be a weighted sum of expression rates from different cell types, although this aspect of C-SIDE is not relevant to potential extensions to FSE testing. Unlike DESeq2, C-SIDE covariates can include continuous distance measures, spatial coordinates, and smooth kernels representing density distributions. So, in the simplest case, a C-SIDE model would take the schematic form of equation 2:


(27) \begin{eqnarray*}
\lambda =f(x,\xi )=\beta _{0} + \beta _{x}x+ \beta _{\xi }\xi
\end{eqnarray*}

This form suggests a general strategy for testing whether a factor $\xi$ has a FSE on $\lambda$: use a GLM with separate spatial and categorical factors $x$ and $\xi$. However, such a model would only tell us whether $\xi$ affects the spatial distribution of $\lambda$ if it included an interaction term:


(28) \begin{eqnarray*}
\lambda =\beta _{0} + \beta _{x}x+ \beta _{\xi }\xi + \beta _{x\times \xi }x\times \xi
\end{eqnarray*}

for $x\times \xi$ defined via standard multiplication as $x\times \xi =x\xi$ and $\xi \in \lbrace 0,1\rbrace$, in which case the model reduces to:


(29) \begin{eqnarray*}
\lambda = \left\lbrace \begin{array}{@{}l@{\quad }l@{}}\beta _{0} + \beta _{x}x& \text{if } \xi = 0 \\\beta _{0} + \beta _{\xi } + (\beta _{x} + \beta _{x\times \xi })x & \text{if } \xi = 1 \end{array}\right.
\end{eqnarray*}

In other words, a GLM with separate spatial and categorical factors $x$ and $\xi$, such as C-SIDE, is at best able to test for a FSE $\beta = \beta _{x\times \xi }$ which modifies the slope of $\lambda$ (if $x$ is a spatial coordinate or distance measure) or the weight of kernel $x$ (if $x$ is a basis density distribution). Hence, this approach is limited in the range of FSEs for which it could test.

Niche-DE [71], as the name implies, is a tool for niche-DE testing. As Mason *et al*. [71] define, a gene is *niche-differentially expressed* (niche-DE) if the cell-type composition of the space around a cell affects the expression level of the gene in that cell. Thus, niche-DE connects gene expression to spatial location indirectly, via modeling the gene expression rate within a cell as a function of a spatially dependent attribute (cell-type composition of the neighborhood around the cell). As Mason *et al*. [71] explain well, this is certainly a valuable kind of analysis to run, but it’s not the same as FSE testing. Niche-DE tests for an effect of space (“spatial niche”) on a non-spatial attribute (gene expression rate), while an FSE test involves testing for an effect of a factor that itself is not necessarily spatial (e.g. age and rearing condition) on spatial attributes (e.g. position-dependent expression rate and expression rate gradient). Thus, there is no straightforward way to turn a test for niche-DE into a test for a FSE, or vice versa.

### Random variance estimation

Whether testing for SVGs or FSEs, it is useful to estimate random effects from variance due to differences between individual tissue samples. This issue is especially pressing, given that many ST studies use small tissue-sample sizes ($n< 5$), including our demonstration here using MERFISH data. The need to estimate random effects in transcriptomics data (both spatial and RNA-Seq) is most acute as it relates to testing for effects from age and other temporal factors such as rearing conditions.

The problem is that temporal factors are of theoretical interest and can be expected to have systematic (i.e. fixed) effects, but current technology usually does not allow for sampling multiple time points from the same individual animal—as seen here in both our own MERFISH data, and the data from Droin *et al*. [14] used for the liver–lobule analysis. Thus, researchers attempting to model transcriptomics data from multiple time points will need to account for the effects of time, but usually have no direct way of experimentally separating temporal effects from random variation between samples. A sufficiently large sample at each time point will allow for estimating whether there is age-related variance that is unlikely given the individual variation within an age, thus solving the problem. However, ST data collection in particular is still extremely costly in terms of both money and time. Thus, more indirect strategies will be necessary for the foreseeable future.

Mixed-effects modeling, i.e. modeling individual variance explicitly through random-effects terms $\rho$, is one such indirect strategy [51]. Along with other strategies (e.g. regularization or “shrinkage”), mixed-effects modeling is being used to account for random effects by a growing number of transcriptomic analysis tools, e.g., C-SIDE [70] and SpaNorm [50]. However, mixed-effects modeling is helpful only if the model is sufficiently constrained. Holding random effects constant across grouping variable levels (i.e., genes) is one way to obtain the needed constraint. We felt it likely that random variation between individuals would depend on the specific gene, and so gave each random level a random effect value for each gene. However, these random effects are assumed to be constant across cell types, thereby introducing the needed constraint when modeling multiple cell types. With WSP models, a second source of constraint comes from including a second factor (e.g. hemisphere) which can be sampled fully within each random level and is orthogonal to, and interacting with, the problematic factor (e.g. age) which can only be sampled once per random level.

Another way to address the problem is with priors. Instead of fitting the model by maximizing the likelihood of the observations given the parameters, the model could be fit by maximizing the product of the likelihood and a prior for the given parameters, which would be equivalent to maximizing the posterior for the parameters. Potential priors could take many forms. For example, the assumption of a normal effect distribution could be codified into a prior by setting


(30) \begin{eqnarray*}
\mathrm{P}(\Phi _J)=\mathcal {N}(\Phi _J\, |\, 0,\sigma _{\Phi })
\end{eqnarray*}

Another approach would be to estimate an expected value $R$ for the ratio of total variance over random variance from the observed counts and set


(31) \begin{eqnarray*}
\mathrm{P}(\Phi ) = \mathcal {N}\left( \mu \left(\frac{|{\rho +\beta }|_{\mathrm{abs}}}{|{\rho }|_{\mathrm{abs}}}\right) \,\,\Bigg | \,\, R,\sigma _{R} \right)
\end{eqnarray*}

A third approach would be to estimate expected random variation $V$ based on prior data and set


(32) \begin{eqnarray*}
\mathrm{P}(\rho )=\mathcal {N}(\mu (|{\rho }|_{\mathrm{abs}})\, |\, V,\sigma _{V})
\end{eqnarray*}

Future versions of WSP models could incorporate one or more of these priors to improve random variance estimation. More broadly, we hope that WSP models raise awareness of the need to handle the small sample sizes in ST data.

### Limitations and future development

WSP models have limitations worth addressing in future development. First, as shown in the benchmarking, parameter estimation takes substantial time and compute power, even for a few dozen genes and a handful of tissue samples. While wispack is already highly optimized, fitting WSP models in 5–30 s per thread, compared to fits during early develop with the R optim function which took over twenty minutes, we estimate that improved optimization methods and more efficient code could get fit times down into the 1–5 s range. However, we don’t believe fit time is a critical issue, as WSP models are meant for theory-driven hypothesis testing. Large-scale data exploration is not feasible, but also is not the intended use.

A second limitation is the need for bootstrapping. While MCMC is possible, we have so far seen mostly poor performance, as explained in the documentation (michaelbarkasi.github.io/wispack/articles/tutorial_stats.html). We assume this is due to the many exponential nonlinearities involved in a WSP model and the complex parameter boundaries. These aspects of WSP models presumably lead to a rocky likelihood landscape. A random walk may not be suitable for navigating this landscape; a directed method such as gradient descent may be needed. The downside of bootstrapping is not only the increased computation time, but also the need for data that is structured in a way allowing for resampling. Improvements to step-size selection (equation B.2) or an alternative to Metropolis–Hastings sampling may make MCMC parameter estimation more reliable.

The constraint to a single spatial dimension $x$ is also a limitation. First, there may be functionally relevant expression distributions over two or more dimensions. Second, the need to align to a single spatial axis of interest both necessitates bespoke coordinate transformations (e.g. Fig. 4C) and leaves room for biased axis selection. Future work on WSP models should aim to generalize the mathematical principles to two or more dimensions.

In the interim, automated methods of axis identification could be used. For example, GASTON [47] uses machine learning to chart contours of constant cell-type composition, called “isodepth,” and extracts the gradient of these contours as a 1D axis for analysis. A WSP model could be applied to data labeled with isodepth coordinates, although this would have the downside of not detecting expression variation which cross-cuts the isodepth gradient.

Statistically, the estimation of dispersion factors (equation 7) is simplistic. However, we see this issue as a minor limitation related to refining the best possible fit. While improvements in fit are always desirable, we believe there is more to be gained from improving the LRO change-point detection algorithm for degree estimation over improving the dispersion factor estimates.

A final question concerns the extent to which the rate, slope scalar and transition point location parameterization is itself a limitation. At least in the case of a single dimension, we think it’s unlikely that there are any biologically realistic transcript distributions which cannot be described by the parameters of a WSP model. However, even so, it’s an open question whether there are hypotheses this parameterization cannot test, e.g. because those hypotheses concern incommensurate distribution parameters. This question can be settled only after seeing the hypotheses researcher attempt to test with WSP model.

## Conclusion

We developed a novel form of logistic regression, WSP models, based around a parameterization for spatial distributions and released it as an open-source R package, wispack. WSP models allow for both random-effect estimation and between-group hypothesis testing with spatial transcriptomic data. We tested their statistical validity on semi-synthetic simulated data and compared the results to two other computational tools for transcriptomic data analysis. To demonstrate the utility of WSP models, we tested for effects of age and laterality on the distribution of ROR$\beta$ expression across the laminar axis of S1 in mouse pups, which is known to play a critical role in the development of cortical whisker-input organization. We also reproduced the spatial analysis of gene expression in liver lobules performed by Droin *et al*. [14]. The results show the utility of WSP models for uncovering subtle molecular asymmetries and testing hypotheses related to biological and cognitive function.

## Supplementary Material

gkag466_Supplemental_File

## Data Availability

S1 MERFISH data: Raw image files are available on the European Bioinformatics Institute’s (EBI) BioImage Archive at ebi.ac.uk/bioimage-archive/, accession number S-BIAD2957, DOI: 10.6019/S-BIAD2957. Vizgen Post-processing Tool output with CCFv3 registration is available as HDF5 files on the NIH Gene Expression Omnibus (GEO) atncbi.nlm.nih.gov/geo/, accession number GSE319949. Liver–lobule data from Droin *et al*. [14] is available at github.com/naef-lab/Circadian-zonation/tree/master/Datasets/Profiles. MERFISH data from Yao *et al*. [55] is available at alleninstitute.github.io/abc_atlas_access/descriptions/MERFISH-C57BL6J-638850.html. Available at github.com/Oviedo-Lab/wspmm_methods and https://doi.org/10.5281/zenodo.19681335 are the following files: S1_laminar_countdata_NARresub_feb14_2026 .csv: final version of the S1 MERFISH data with transformation into laminar coordinates. Droin_radial_count_data_sim.csv: preprocessed version of the Droin *et al*. [14] data used here. Allen_data.csv: seed data used for attractor simulations. benchmark_results_NARresub_feb14_2026 .csv: results of running the 250 attractor-simulation benchmarking. This Git Repo also contains all the code used in this paper, excluding wispack.

## Appendix 1 Model fitting

### 1.1 Degree estimation

Estimating the degree $d$ for each gene $g$ within each cell type ${c}$ amounts to estimating the number of transition points in the kernel rate $\lambda$ along spatial dimension $x$. To this end, we adopt the basic idea behind likelihood-ratio outlier (LRO) change-point detection algorithms [54]. We assume that $d(g,{c})$ is fixed for each combination of $g$ and ${c}$, but that the positions and slopes of transition points potentially vary between both treatment levels $w_j= \xi _{j_1}\times \ldots \times \xi _{j_m}$ and random-effect levels $k$ of $\nu$. To estimate a single degree for each gene within each cell type, the relative likelihood of a transition point compared to the likelihood of no transition point is computed separately for observations across treatment and random levels. These likelihood ratios are aggregated into a centroid and it is this centroid which is tested for transition points by searching for outliers.

Specifically, a matrix $\mathcal {C}$ is constructed which organizes the observed counts $y$ for a gene $g$ within cell type ${c}$ by spatial coordinates $x$ (rows) and the interaction between treatment conditions $w_j$ and random levels $k$ (columns). Each column of this matrix $\mathcal {C}$ gives the total transcript count $y$ for $g$ within ${c}$ in each bin of $x$, observed for a given random level $k$ under treatment condition $w_j$. Columns representing a random level not observed under that column’s treatment condition are thrown out.

(A.1)
\begin{eqnarray*}
&&\,\,\,\,\,\,\,\,\,\,\,\,\,\,\, \,\,\,\,\,\,\,\,\,\,\,\, \,\,\,\,\,\,\,\,\,\, w_1 \,\,\,\,\,\,\,\,\,\, \,\,\,\,\,\,\,\,\,\, \,\,\,\,\,\,\,\,\,\, \,\,\,\,\,\,\,\,\,\, \,\,\,\,\,\,\,\,\qquad w_l\nonumber \\
&&\,\,\,\,\,\,\,\,\,\,\,\,\,\,\,\,\,\,\,\,\, \overbrace{k_1 \,\,\,\,\,\,\,\,\,\, \cdots \,\,\,\,\,\,\,\,\,\, k_c} \quad \cdots \quad \overbrace{k_1 \,\,\,\,\,\,\,\,\,\, \cdots \qquad k_c}\nonumber \\
&& \mathcal {C}= \begin{bmatrix}y_{{x_1}11} & \cdots & y_{{x_1}1c} & \cdots & y_{{x_1}l1} & \cdots & y_{{x_1}lc} \\
y_{{x_2}11} & \cdots & y_{{x_2}1c} & \cdots & y_{{x_2}l1} & \cdots & y_{{x_2}lc} \\
\vdots & \ddots & \vdots & \ddots & \vdots & \ddots & \vdots \\
y_{{x_{b_{p}}}11} & \cdots & y_{{x_{b_{p}}}1c} & \cdots & y_{{x_{b_{p}}}l1} & \cdots & y_{{x_{b_{p}}}lc} \\
\end{bmatrix} \begin{array}{l}x_1\\
x_2\\
\vdots \\
x_{b_{p}}\\
\end{array}
\end{eqnarray*}

The count values in this matrix are transformed into log space as in equation 8:

(A.2)
\begin{eqnarray*}
\mathcal {C}_{xjk} \mapsto \mathrm{log}_e(\mathcal {C}_{xjk}+1) = \mathcal {C}_{xjk}^\prime
\end{eqnarray*}

For each column $\mathcal {C}_{jk}^\prime = \langle \mathcal {C}_{x_1jk}^\prime , \ldots , \mathcal {C}_{x_{b_{p}}jk}^\prime \rangle$ we estimate, for each point $x_1,\ldots ,x_{b_{p}}$, the log of the ratio of the likelihood $\ell _{\mathrm{cp}}$ of a change in kernel rate over the likelihood $\ell _{\mathrm{null}}$ of no change in kernel rate:

(A.3)
\begin{eqnarray*}
L_{\mathrm{ratio}}\left(\mathcal {C}_{xjk}^\prime \right) = \mathrm{log}_e\left( \frac{ \ell _{\mathrm{cp}}\left(\mathcal {C}_{xjk}^\prime \right) }{ \ell _{\mathrm{null}}\left(\mathcal {C}_{xjk}^\prime \right) } \right)
\end{eqnarray*}

These individual ratios are collected into their own column:

(A.4)
\begin{eqnarray*}
L_{\mathrm{ratio}}\left(\mathcal {C}_{jk}^\prime \right) = \left\langle L_{\mathrm{ratio}}(\mathcal {C}_{x_{1}jk}^\prime ), \ldots , L_{\mathrm{ratio}}(\mathcal {C}_{x_{b_{p}}jk}^\prime ) \right\rangle
\end{eqnarray*}

The logs are taken for numerical stability. The likelihood ratios themselves are taken as a measure of how much better a given point $x$ is modeled as a transition point vs as a point of constant expression.

The ratios $L_{\mathrm{ratio}}$ are computed using local windows $\mathcal {X}$ around the points $x$. To compute their value, we use the quotient identity:

(A.5)
\begin{eqnarray*}
\mathrm{log}_e\left( \frac{ \ell _{\mathrm{cp}}\left(\mathcal {C}_{xjk}^\prime \right) }{ \ell _{\mathrm{null}}\left(\mathcal {C}_{xjk}^\prime \right) } \right) = L_{\mathrm{cp}}\left(\mathcal {C}_{xjk}^\prime \right) - L_{\mathrm{null}}\left(\mathcal {C}_{xjk}^\prime \right)
\end{eqnarray*}

To compute $L_{\mathrm{cp}}$ and $L_{\mathrm{null}}$ for a point $x$ in column $jk$, let $\mathcal {X}$ be a window of points centered on $x$. This set of points defines a set of log-linked counts $\mathcal {C}_{\mathcal {X}jk}^\prime$:

(A.6)
\begin{eqnarray*}
\mathcal {C}_{\mathcal {X}jk}^\prime = \lbrace \mathcal {C}_{xjk}^\prime \rbrace _{x\in \mathcal {X}}
\end{eqnarray*}

The log-likelihood of the null hypothesis that there is no change-point is defined:

(A.7)
\begin{eqnarray*}
L_{\mathrm{null}}\left(\mathcal {C}_{xjk}^\prime \right)= \sum _{x\in \mathcal {X}} \mathrm{log}_e\left( \mathcal {N}\left( \mathcal {C}_{xjk}^\prime \, |\, \mu (\mathcal {C}_{\mathcal {X}jk}^\prime ), \sigma (\mathcal {C}_{\mathcal {X}jk}^\prime ) \right) \right)
\end{eqnarray*}

For each window $\mathcal {X}$, the log-likelihood that the window contains a transition point at its center can be computed by summing the log-likelihoods of the null hypothesis for the points $x$ in the first half $\mathcal {X}_{\frac{1}{2}}$ and second half $\mathcal {X}_{\frac{2}{2}}$ of the window:

(A.8)
\begin{eqnarray*}
\begin{aligned} & L_{\mathrm{cp}}\left(\mathcal {C}_{xjk}^\prime \right) = \\& \,\,\,\,\,\, \sum _{x\in \mathcal {X}_{\frac{1}{2}}} \mathrm{log}_e\left( \mathcal {N}\left( \mathcal {C}_{xjk}^\prime \, |\, \mu (\mathcal {C}_{\mathcal {X}_{\frac{1}{2}}jk}^\prime ), \sigma (\mathcal {C}_{\mathcal {X}_{\frac{1}{2}}jk}^\prime ) \right) \right) + \\& \,\,\,\,\,\, \sum _{x\in \mathcal {X}_{\frac{2}{2}}} \mathrm{log}_e\left( \mathcal {N}\left( \mathcal {C}_{xjk}^\prime \, |\, \mu (\mathcal {C}_{\mathcal {X}_{\frac{2}{2}}jk}^\prime ), \sigma (\mathcal {C}_{\mathcal {X}_{\frac{2}{2}}jk}^\prime ) \right) \right) \end{aligned}
\end{eqnarray*}

Three different window sizes are used, giving three estimates for each ratio. Multiple window sizes are used because the size of the window corresponds to the frequency of transition points detectable by that window, with smaller windows acting as high-pass filters and larger windows acting as low-pass filters. In wispack, the high-pass window size is a user-defined multiple of the transition-point buffer factor $p_\mathrm{buffer}$ (default in wispack v2.0 of 1.25) and the mid-pass and low-pass window sizes are $2\times$ and $4\times$ multiples of the high-pass window size.

Next, three matrices $L(\mathcal {C})$ of the log-likelihood ratios $L_{\mathrm{ratio}}$ are defined, one for each filter, as follows:

(A.9)
\begin{eqnarray*}
L(\mathcal {C})_{xjk} = L_{\mathrm{ratio}}\left(\mathcal {C}_{jk}^\prime \right)_{x}
\end{eqnarray*}

Taking the means across the rows of these new matrices gives, for each of the three filters, an estimate of the centroid $\mathcal {O}$ of the transition-point log-likelihood ratio over $x$ across all combinations of treatment and random-factor levels:

(A.10)
\begin{eqnarray*}
\mathcal {O}= \left\langle \mu \left(L(\mathcal {C})_{x_1}\vphantom{L(\mathcal {C})_{x_{b_{p}}}}\right), \ldots , \mu \left(L(\mathcal {C})_{x_{b_{p}}}\right) \right\rangle
\end{eqnarray*}

A point $x$ is treated as a transition point $p$ if and only if, for at least one of the high-pass, mid-pass, or low-pass $\mathcal {O}$, the element $\mathcal {O}_x$ in $\mathcal {O}$ is more than some multiple $\tau$ of the standard deviation of $\mathcal {O}$ above the mean:

(A.11)
\begin{eqnarray*}
\mathcal {O}_x> \mu (\mathcal {O}) + \tau \sigma (\mathcal {O})
\end{eqnarray*}

By default in wispack (v2.0), $\tau =2.0$, on the assumption that transition points rarely make up >5% of bins $x$. Potential transition points too close to zero or $b_{p}$ are thrown out, and if two potential transition points are too close together, the point with the larger of the two log-likelihood ratios is used. “Too close” is defined as within a distance of half the size of the high-pass filter window.

### 1.2 Initial parameter values

WSP models are fit to observations $y$ by finding the effects parameters $\beta$ and $\rho$ which, given the estimated dispersion factors $\zeta$ (equation 7), maximize the joint likelihood of those observations within the log-link. This is done by optimizing an initial set of parameter values using gradient descent. Initial values for the random effects $\rho$ are picked from a uniform random distribution spanning $-0.1$ to 0.1. Model fitting is greatly improved if the fixed effects are initialized close to their optimal values, and so the points $p$ determined by the LRO outlier threshold (equation A.11) are used to set, for all genes $g$, cell types ${c}$, and treatment levels $w_j$, initial values for all fixed effects $\beta _{j}$. The following procedure is performed separately on each combination of gene $g$ and cell type ${c}$.

The LRO outlier threshold only estimates a single set of transition points $p$ spanning all conditions for a gene and cell type, so the first step is to estimate the transition point locations for each combination of treatment and random-effect level. A dynamic time-warping algorithm [72] is used to align each column of the mid-pass $L(\mathcal {C})$ to the mid-pass centroid $\mathcal {O}$ (equation A.10). The resulting alignments (one per column) are used to project the points $p$ onto the row numbers of $L(\mathcal {C})$, yielding one projection $\hat{p}$ per column of $L(\mathcal {C})$. For this step wispack uses the dtw package in R (v1.23-1, [73]) with the warping assuming a symmetric step pattern of slope 0.5 [74]. These projections are collected into a new matrix $\mathcal {C}_{\mathcal {O}}$ with the column structure of $\mathcal {C}$, the number of rows of which is the degree (i.e. is the number of transition points).

The second step is to estimate transition point locations $p$ for each treatment level $w_j$ by taking the mean across the rows of $\mathcal {C}_{\mathcal {O}}$ within columns under $w_j$. The resulting subsetted and factored matrix has the form:

(A.12)
\begin{eqnarray*}
&& \qquad\,\,\,\,\,\, w_1 \,\,\, \cdots \,\,\, w_l \\&& \mathcal {P}= \begin{bmatrix}\hat{p}_{{1}1} & \cdots & \hat{p}_{{1}l} \\\hat{p}_{{2}1} & \cdots & \hat{p}_{{2}l} \\\vdots & \ddots & \vdots \\\hat{p}_{{{d}}1} & \cdots & \hat{p}_{{{d}}l} \\\end{bmatrix} \begin{array}{l}1 \\2 \\\vdots \\{d}\\\end{array}
\end{eqnarray*}

For each transition point index $i$ ($1\le i\le d$), the row $\mathcal {P}_i$ provides an estimate of the transition point location observed under each treatment condition $j$. These estimates can be used to define $d+1$ blocks of points $x$, such that block $i$ includes $\mathcal {P}_{(i-1)j}\le x\le \mathcal {P}_{ij}$ with $\mathcal {P}_{0j}=0$ and $\mathcal {P}_{(d+1)j}=b_{p}$.

Next, from these blocks a rate matrix $\mathcal {R}$ can be defined such that $\mathcal {R}_{ij}$ is the mean of the log (plus one) of the observed counts $y$ in block $i$ for treatment $w_j$. Similarly, a slope matrix $\mathcal {S}$ can be defined such that initial estimates $\mathcal {S}_{ij}$ for the slope scalars are calculated by finding the number of bins (points $x$) which must be stepped ahead and behind transition point $\hat{p}_{ij}$ such that the difference of the means of the log (plus one) of the observed counts before and after $\hat{p}_{ij}$ is greater than or equal to some stipulated proportion (by default in wispack v2.0, 0.8) of $\mathcal {R}_{(i+1)j} - \mathcal {R}_{ij}$. This number of bins is the run of the slope, and the implied slope scalar is 4 divided by the run (Appendix 4.2).

To use the initial estimates $\mathcal {R}$, $\mathcal {S}$, and $\mathcal {P}$ of the spatial parameters to set the initial values of the effects $\beta _{r}$, $\beta _{s}$, and $\beta _{p}$, we first define a weight matrix:

(A.13)
\begin{eqnarray*}
&& \,\,\,\,\,\,\,\,\,\,\,\,\,\,\,\,\,\,\,\,\,\,\,\, w_1 \,\,\,\,\,\,\,\,\,\,\,\, \cdots \,\,\,\,\,\,\,\,\,\,\,\, w_l \\&& \mathcal {W}= \begin{bmatrix}w_1(\xi _{w_1}) & \cdots & w_l(\xi _{w_1}) \\w_1(\xi _{w_2}) & \cdots & w_l(\xi _{w_2}) \\\vdots & \ddots & \vdots \\w_1(\xi _{w_l}) & \cdots & w_l(\xi _{w_l}) \\\end{bmatrix} \begin{array}{l}\xi _{w_1}\\\xi _{w_2}\\{\vdots }\\\xi _{w_l}\\\end{array}
\end{eqnarray*}

For convenience, “$\xi _{w_j}$” is short-hand for the factor $\xi$ which has the components of $w_j$ at treatment level and all other components at reference level. Intuitively, $\mathcal {W}$ says whether the treatment represented by a column applies (i.e. is weighted 1) in the treatment condition represented by each row. By equation 12, for each transition point or block $i$, treatment level $j$, and spatial parameter matrix $X$ (i.e. $X$ one of $\mathcal {R}$, $\mathcal {S}$, and $\mathcal {P}$), the initial effects $\beta _{Xij}$ can be got by solving the following system of equations:

(A.14)
\begin{eqnarray*}
\mathcal {W}\left\langle \beta _{Xi1},\ldots , \beta _{Xi{}l}\right\rangle ^{\mathrm{T}} = \left(X_{i}\right)^{\mathrm{T}}
\end{eqnarray*}

Full-pivot LU decomposition [75] is used in wispack to solve this system.

### 1.3 Parameter optimization

With initial parameters estimated, the next step is to maximize the joint likelihood of the observations $y$ within the log-link using gradient descent. It is assumed that values $y$ depend only on the effects $\beta$ and $\rho$ and the dispersion factor $\zeta$, and so are marginally independent of all other factors. For example, while expression rates of genes $g$ are surely correlated within a cell type ${c}$, this correlation is captured in the effects and dispersion factor. Thus, the joint likelihood of all values $y$ is simply the product of their individual likelihoods. For numerical stability, the negative of the log of the joint likelihood (now a sum) is minimized:

(A.15)
\begin{eqnarray*}
\mathcal {L}(y\, |\, \beta ,\rho ,\zeta ) = -\sum _{y}\mathrm{log}_e\left( \ell (y\, |\, \Psi ,\widetilde{{\sigma _{\gamma }^2}}) \right)
\end{eqnarray*}

where the likelihood is:

(A.16)
\begin{eqnarray*}
\begin{aligned} &\ell (y\, |\, \Psi ,\widetilde{{\sigma _{\gamma }^2}}) = \\&\,\,\,\, \int _0^\infty \mathrm{Pois}(\mathrm{log}_e(y+ 1)\, |\, \Lambda )\mathrm{Gam}(\Lambda \, |\, \Psi ,\widetilde{{\sigma _{\gamma }^2}})d\Lambda \end{aligned}
\end{eqnarray*}

The integral in this equation can be solved analytically (Appendix 4.4), which greatly simplifies the minimization. Given that both $y$ and $\Psi$ are log-linked (equation 8), a log-linked version of ${\sigma _{\gamma }^2}$ must be used in equations A.15 and A.16. Using the tilde to represent the transform into, or out of, the log link (e.g., $\widetilde{\Psi } = e^{\Psi } - 1$) and keeping with the interpretation of $\zeta$ as a dispersion factor (equation 6), the variance of the kernel can be approximated using the delta method [76] as:

(A.17)
\begin{eqnarray*}
\widetilde{{\sigma _{\gamma }^2}} \approx {\sigma _{\gamma }^2}\left(\frac{\partial (\mathrm{log}_e(X) + 1)}{\partial X}\biggr \vert _{X= \widetilde{\Psi }}\right)^2 = \frac{\widetilde{\Psi } + \zeta \widetilde{\Psi }^2}{(\widetilde{\Psi } + 1)^2}
\end{eqnarray*}

When minimizing the joint log-likelihood of the observations $y$ within the log-link, all model parameters must stay within bounds of feasibility. Specifically, (1) all slope scalars $s$ must be positive, (2) transition points $p$ must be in increasing order, not too close together, and must be between zero and the bound $b_{p}$, and (3) the predicted value $\Psi$ must always be positive. Regarding this last constraint, $\Psi > 0$ if and only if $r_i> 0$ for all rate components used in the calculation of $\Psi$ (Appendix 4.5). Thus, it suffices to ensure that all rate components are positive.

To enforce these boundaries, for each possible combination $g\times {c}\times \xi \times k$ of $g$, ${c}$, $\xi$, and $k$, the bounded variables are computed and collected in a set:

(A.18)
\begin{eqnarray*}
\mathcal {B}_{g{c}\xi k} = s(g,{c},\xi ,k) \cup \Delta p(g,{c},\xi ,k) \cup r(g,{c},\xi ,k) \\
\end{eqnarray*}

In this equation, $s(g,{c},\xi ,k)$ and $r(g,{c},\xi ,k)$ are the slopes and rates determined by the given combination of gene, cell type, fixed-effects, and random-effect level (equation 22). The middle term $\Delta p(g,{c},\xi ,k)$ captures the constraints on transition points. It is defined:

(A.19)
\begin{eqnarray*}
\begin{aligned} &\Delta p(g,{c},\xi ,k) = \\&\,\,\,\,\,\,\lbrace p_{i+ 1}^\prime (g,{c},\xi ,k) - p_{i}^\prime (g,{c},\xi ,k) - b_{p}p_\mathrm{buffer}\rbrace _{i= 0}^{d(g,{c}) + 1} \end{aligned} \\
\end{eqnarray*}

in terms of an extension $p^\prime$ of $p$:

(A.20)
\begin{eqnarray*}
p^\prime (g,{c},\xi ,k) = \langle 0, p_{1}(g,{c},\xi ,k), \ldots , p_{d(g,{c})}(g,{c},\xi ,k), b_{p} \rangle \\
\end{eqnarray*}

Notice that this equation defines “too close together” as a distance smaller than $b_{p}p_\mathrm{buffer}$, for a scalar $p_\mathrm{buffer}$ between zero and one (set to 0.05 by default in wispack v2.0).

The total set of boundary distances is the union of all of these sets:

(A.21)
\begin{eqnarray*}
\mathcal {B}= \bigcup _{g\times {c}\times \xi \times k} \mathcal {B}_{g{c}\xi k}
\end{eqnarray*}

and from this set $\mathcal {B}$ a boundary penalty is defined:

(A.22)
\begin{eqnarray*}
{\mathcal {B}^\times }= \sum _{\delta \in \mathcal {B}} \frac{1}{(\delta B_{\delta })^2}
\end{eqnarray*}

This penalty is nearly zero when all boundary distances $\delta$ in $\mathcal {B}$ are far above zero ($\delta \ge 1$) and smoothly approaches infinity exponentially as any one of those distances approach zero. It is added to the negative log-likelihood $\mathcal {L}$ (equation A.15) to obtain the value to be minimized:

(A.23)
\begin{eqnarray*}
\mathcal {L}^\prime (y\, |\, \beta ,\rho ,\zeta ) = \mathcal {L}(y\, |\, \beta ,\rho ,\zeta ) + {\mathcal {B}^\times }
\end{eqnarray*}

To keep the penalty term ${\mathcal {B}^\times }$ near zero when all $\delta$ are far above zero, a weighting coefficient $B_{\delta }$ is included for each distance $\delta$ such that, for $\varepsilon$ some near-zero penalty value and the boundary distances computed from the set of initializing effects parameters $\beta$ and $\rho$:

(A.24)
\begin{eqnarray*}
B_{\delta } = \frac{ 1 }{ \delta _{\mathrm{initial}} } \sqrt{ \frac{ \left|{\mathcal {B}}\right|_{\#} }{ \varepsilon } }
\end{eqnarray*}

The minimization of $\mathcal {L}^\prime$ is performed in wispack using the L-BFGS algorithm (NLopt, [77, 78]) with gradients calculated via reverse-mode automatic differentiation (Stan, [79]).

## Appendix 2 Statistical methods

WSP models test the hypothesis that factor $\xi$ has effect $\beta _\xi$ on gene-expression distribution $z$ by estimating the probability of a given value $\beta _{z\xi }$, conditional on observed transcript counts $y$. That is, the conditional joint distribution $\mathrm{P}(\beta ,\rho \, |\, y,\zeta )$ is estimated. This can be done by resampling $\beta$ and $\rho$, either (1) using a Metropolis–Hastings random walk for MCMC estimation, or (2) by refitting the model to resamples of $y$ with new estimates of $\zeta$ (bootstrapping). As fixed $\beta$ and random $\rho$ effect parameters are handled the same when resampling, we use the variable $\Phi$ to cover both cases and use $J$ to index the parameters in $\Phi$.

### 2.1 Resampling

MCMC resampling is done in wispack with a Metropolis–Hastings algorithm [80] which steps from one parameter vector $\Phi$ to the next $\Phi ^+$ by random walk, starting with the same parameters used to initialize the L-BFGS algorithm. Steps are drawn from a normal distribution:

(B.1)
\begin{eqnarray*}
\Phi ^{+}_J\sim \mathcal {N}\left( \Phi _{J}, \sigma _{\Phi _J} \right)
\end{eqnarray*}

The values in $\Phi$ can differ by orders of magnitude and must stay within the boundaries of feasibility. Thus, step size $\sigma _{\Phi _J}$ is a multiple $\phi$ of the absolute value of the parameter $\Phi _J$ (plus one) and further scaled by the boundary penalty $\mathcal {B}_{\Phi }^\times$ of $\Phi$ (equation A.22):

(B.2)
\begin{eqnarray*}
\sigma _{\Phi _J} = \frac{ \phi (|{\Phi _J}|_{\mathrm{abs}} + 1) }{ 1 + \mathcal {B}_{\Phi }^\times }
\end{eqnarray*}

Aside from the reference values $\beta _{pi1}$ for the transition points, which are assumed to be uniformly distributed, the prior $\mathrm{P}_{\mathrm{prior}}(\Phi _J)$ of each $\Phi _J$ is calculated from the density function of a normal distribution centered on zero with adjustable $\sigma$. The precise value of $\sigma$ is not crucial, so long as it is around the size of a typical parameter in $\Phi$. By default in wispack v2.0, $\sigma =1$. The bell shape of this prior captures the assumption that larger values are less likely than smaller ones. The overall log-prior $\mathrm{log}_e(\mathrm{P}_{\mathrm{prior}}(\Phi ))$ is computed as the sum of all $\mathrm{log}_e(\mathrm{P}_{\mathrm{prior}}(\Phi _J))$. Log-likelihoods $L(y\, |\, \Phi ,\zeta )$ are computed according to equation A.23 as $L(y\, |\, \Phi ,\zeta )=-\mathcal {L}^\prime (y\, |\, \Phi ,\zeta )$. The posteriors are computed as:

(B.3)
\begin{eqnarray*}
\mathrm{log}_e(\mathrm{P}_{\mathrm{post}}(\Phi ))=L(y\, |\, \Phi ,\zeta ) + \mathrm{log}_e(\mathrm{P}_{\mathrm{prior}}(\Phi ))
\end{eqnarray*}

The acceptance ratio $a$ for a new step $\Phi ^{+}$ is then given by:

(B.4)
\begin{eqnarray*}
\mathrm{log}_e(a) = \mathrm{log}_e(\mathrm{P}_{\mathrm{post}}(\Phi ^{+})) - \mathrm{log}_e(\mathrm{P}_{\mathrm{post}}(\Phi ))
\end{eqnarray*}

Bootstrapping is viable and preferred if the structure of the data allows for resampling and computational resources allow for refitting. Bootstraps can be run in parallel by wispack using forking on Mac and Linux systems; wispack does not provide parallelization for Windows. The MERFISH cortex data used here provide such an example. In these data, the total count $y_x$ in a bin $x$ for gene $g$ of cell type ${c}$ is the sum of the counts of $g$ in each cell of type ${c}$ in $x$. Thus, the cells themselves can be resampled with replacement and the counts summed again to get a resampling of $y_x$. After resampling every gene $g$ in every bin $x$ for each cell type ${c}$, a WSP model can be fit again to the resampled observations $y^{+}$ to get a new parameter estimate $\Phi ^{+}$. Before estimating $\Phi ^{+}$ via gradient descent, new dispersion factors $\zeta$ are computed from $y^{+}$ using equation 7 and new initial parameter values are computed using the procedure described in appendix 1.2, although the original degree estimation is used in all bootstrap resamples.

For both bootstrap resampling and the Metropolis–Hastings random walk, wispack uses a permuted congruential generator (PCG) implemented in C++ for random number generation. PCGs outperform standard algorithms like Mersenne Twister in computational speed, unpredictability, and conformity to expected statistical behavior [81]. The PCG provides sampling from a uniform distribution, which is used directly for bootstraps and transformed into a normal distribution with the Box-Muller algorithm for the random walk.

### 2.2 Confidence intervals

Whether by MCMC or bootstrapping, resamples $I$ yield a matrix $\overline{\Phi }$ of resampled parameters $\Phi _{IJ}$. To clarify notation, “$\Phi _J$” will continue to represent a scalar value for parameter $J$, while “$\langle \Phi \rangle _{J}$” and “$\langle \Phi \rangle _{I}$” represent the column of $\overline{\Phi }$ for parameter $J$ and row of $\overline{\Phi }$ for resample $I$, respectively. Each row $\langle \Phi \rangle _{I}$ is thus a complete parameter vector $\Phi$. A confidence interval for significance level $\alpha$ can be computed for each parameter $J$ by estimating the first and last ($\alpha /2$)-quantile of column $\langle \Phi \rangle _{J}$:

(B.5)
\begin{eqnarray*}
\mathrm{CI}(J,\alpha ) = \langle Q_J(\alpha /2), Q_J(1-\alpha /2)\rangle
\end{eqnarray*}

The quantiles $Q_J$ are computed over $\langle \Phi \rangle _{J}$ with the standard linear interpolation used in R (type 7, [82]).

### 2.3 *p*-values

For a specific observed sample $I$, a $p$-value can be computed from the empirical cumulative distribution function ($\mathrm{ecdf}$), defined for an input vector $X$ and scalar value $X^\prime$:

(B.6)
\begin{eqnarray*}
\mathrm{ecdf}(X, X^\prime ) = \frac{ \left|{ \lbrace X\le X^\prime \rbrace }\right|_{\#} }{ \left|{X}\right|_{\#} }
\end{eqnarray*}

The $p$-value for the observed parameter $\Phi _{IJ}$, with null hypothesis that $\Phi _{J} = 0$, is computed by first recentering the sampled values $\langle \Phi \rangle _{J}$ around the null:

(B.7)
\begin{eqnarray*}
\langle \Phi \rangle _{J}^{\mu =0} = \langle \Phi _{J} - \mu (\langle \Phi \rangle _{J}) \rangle _{J}
\end{eqnarray*}

and then estimating the probability of observing parameter $J$ with at least the magnitude $|{\Phi _{IJ}}|_{\mathrm{abs}}$, assuming the null distribution:

(B.8)
\begin{align*}
p(\Phi _{IJ}) = 1 &- \mathrm{ecdf}\left( \langle \Phi \rangle _{J}^{\mu =0}, |{\Phi _{IJ}}|_{\mathrm{abs}} \right) \\& + \mathrm{ecdf}\left( \langle \Phi \rangle _{J}^{\mu =0}, -|{\Phi _{IJ}}|_{\mathrm{abs}} \right)
\end{align*}

While $p$-values can be computed for any of the resampled parameters, presumably it only makes sense to compute them for some distinguished set of parameters representing the best estimate, e.g. those parameters found by the L-BFGS optimization on the observed data, or an average taken from the resampled parameters. Further, while confidence intervals can be computed for all parameters, the $\mathrm{ecdf}$ method (equation B.8) only makes sense when the null hypothesis is that the parameter has value zero (presumably representing no effect). Thus, at most the $\mathrm{ecdf}$ method makes sense for random-effect parameters $\rho$ and fixed-effect parameters $\beta _{j}$ such that $j\ne 1$, i.e. fixed-effect parameters except for the baseline level. The $\mathrm{ecdf}$ method works for these parameters because they were constructed to have unconstrained distributions with center zero representing no effect.

In contrast, there is no similarly direct test for a $p$-value from the resamples of the baseline parameters (i.e. $j=1$), which all have a floor of zero. While there are ways around this difficulty, such as a maximum likelihood ratio test comparing the unconstrained model to one with the target baseline parameter held to zero, these approaches raise new issues, most notably the problem of estimating degrees of freedom. The aim of WSP models is to test for FSEs, not to test whether a given gene is an SVG (non-zero slope scalar) or to test whether that gene is being expressed at all (non-zero baseline rate parameters). Thus, we make no recommendations on how to calculate $p$-values for the baseline parameters.

While the $\mathrm{ecdf}$ method can be applied to the random effect parameters, wispack does not apply it to them. This is because of the aim of testing for FSEs, but also to limit the number of statistical tests run. Even a handful of genes and cell types typically will lead to hundreds of effects parameters in a WSP model, so running additional tests with no clear purpose is not advised. To handle the inevitable multiple comparisons problem with testing even just the fixed effects (for $j\ne 1$), wispack by default adjusts confidence intervals and $p$-values with a Holm–Bonferroni correction, with the more conservative Bonferroni correction available as well.

## Appendix 3 Wispack MWE

Instructions on how to install wispack are available at michaelbarkasi.github.io/wispack/articles/install.html. The minimum input required to use wispack is a data frame in the format used by standard R functions for linear modeling. For example, when modeling data with the formula observation ∼ factor1 * factor2, standard functions like lm expect a data frame with columns named observation, factor1, and factor2, with each row being an observation. Calling the wisp function with such a data frame will fit a WSP model to the data, estimate confidence intervals and $p$-values, and output a number of data frames and summary plots with results. For example, a version without cell-type labels of the S1 MERFISH data used in this demonstration is provided in the git repository for wispack. The following code fits a WSP model to it with default settings:

Unlike standard functions for linear models in R, wisp does not take a formula specifying a model, as the model formula is always of the same form (equation 10). What varies are the variables to use as fixed effects, random effects, etc. These are specified by giving the variables argument a list of elements naming columns in the data, each labeled with the model component given by that element (count for observed counts, bin for the spatial coordinate, ran for the random effect variable, etc):

If no variables are specified, wispack looks for columns in the data with names matching the labels in the data.variables example list. There are many other settings, e.g., for controlling the MCMC walk, running bootstraps, adjusting the LRO algorithm, etc. These are all shown in the full_options.R demo in wispack.

## Appendix 4 Proofs and algorithms

### 4.1 Sigmoid rate theorem

**Theorem:** If $s_i\gg 0$ for all $i$ and $i^\prime$ is the largest $i$ such that $x\gg p_i$, then $\Psi _{d}(x,r,s,p) \approx r_{i^\prime +1}$.

**Proof:** By definition (equation 10):

\begin{eqnarray*}
\Psi _{d}(x,r,s,p) = r_1 + \sum _{i=1}^{d} \frac{ r_{i+1} - r_i}{ 1 + e^{-s_i(x- p_i)} }
\end{eqnarray*}

For all $i$, if $s_i\gg 0$, the tendency of the exponential in the denominator of the summand to be either a very large value or a value near zero implies that:

\begin{eqnarray*}
\frac{ r_{i+1} - r_i}{ 1 + e^{-s_i(x- p_i)} } \approx \left\lbrace \begin{array}{@{}l@{\quad }l@{}}r_{i+1} - r_i& \text{if }x\gg p_i\\0 & \text{if } x\ll p_i\end{array}\right.
\end{eqnarray*}

It follows that:

\begin{eqnarray*}
\Psi _{d}(x,r,s,p) \approx r_1 + \negthickspace \negthickspace \sum _{i:\,\, x\gg p_i} \negthickspace \negthickspace r_{i+1} - r_i
\end{eqnarray*}

Using $i^\prime$ for the largest $i$ such that $x\gg p_i$, this can be rewritten as:

\begin{eqnarray*}
\Psi _{d}(x,r,s,p) \approx r_1 - r_1 + r_{2} - \ldots - r_{i^\prime } + r_{i^\prime +1}
\end{eqnarray*}

In the term on the right-hand side, all $r_i$ cancel out except for the last, leaving $\Psi _{d}(x,r,s,p) \approx r_{i^\prime +1}$.

### 4.2 Sigmoid slope theorem

**Theorem:**

\begin{eqnarray*}
\frac{\partial \Psi _{d}}{\partial x}\biggr \vert _{x=p_u} \approx (r_{u+1}-r_u)s_u/4
\end{eqnarray*}

**Proof:** By the linearity of derivatives and definition of $\Psi$ in equation 10:

\begin{eqnarray*}
\frac{\partial \Psi _{d}}{\partial x} = \sum _{i=1}^{d} \frac{\partial }{\partial x} \frac{ r_{i+1} - r_i}{ 1 + e^{-s_i(x- p_i)} }
\end{eqnarray*}

By the quotient rule and basic algebra, this equals:

\begin{eqnarray*}
\sum _{i=1}^{d} -\frac{ (r_{i+1} - r_i) }{ (1 + e^{s_i(p_i- x)})^2 } \frac{\partial }{\partial x}(1 + e^{s_i(p_i- x)})
\end{eqnarray*}

By the linearity of derivatives, the chain rule, and more algebra, this equals:

\begin{eqnarray*}
\sum _{i=1}^{d} \frac{ (r_{i+1} - r_i)s_ie^{s_i(p_i- x)} }{ (1 + e^{s_i(p_i- x)})^2 }
\end{eqnarray*}

Evaluating this formula at $x=p_u$ gives:

\begin{eqnarray*}
\sum _{i=1}^{d} \frac{ (r_{i+1} - r_i)s_ie^{s_i(p_i- p_u)} }{ (1 + e^{s_i(p_i- p_u)})^2 }
\end{eqnarray*}

Note that for all $X$, $e^{X}/(1+e^{X})^2\approx 0$ unless $X\approx 0$. In our case, $X=s_i(p_i-p_u)$, which only approaches zero if $s_i\approx 0$ or $p_i\approx p_u$. The former is impossible, given the constraint on the slope parameter that $s> 0$. The latter is also impossible when $i\ne {}u$, given the constraint that there exist a nontrivial buffer distance between all transition points $p$. Thus:

\begin{eqnarray*}
\frac{\partial \Psi _{d}}{\partial x} \biggr \vert _{x=p_u} \approx \frac{ (r_{u+1} - r_u)s_ue^{s_u(p_u - p_u)} }{ (1 + e^{s_u(p_u - p_u)})^2 } = (r_{u+1}-r_u)s_u/4
\end{eqnarray*}

### 4.3 Warping limit theorem

**Theorem:** Equation 21 is true:

\begin{eqnarray*}
\lim _{b_{q}\rightarrow \infty }\omega (z_q,\rho _{q},b_{q}) = \left\lbrace \begin{array}{@{}l@{\quad }l@{}}z_q(1 + (\rho _{q})^2) & \text{if } z_q\ge 0 \\z_qe^{-(\rho _{q})^2} & \text{if } z_q< 0 \end{array}\right.
\end{eqnarray*}

**Proof:** Begin by considering the limit of the warping ratio (equation 19) as the bound $b$ approaches infinity:

\begin{align*}
\lim _{b\rightarrow \infty } \varphi (X,\rho ,b) &= \lim _{b\rightarrow \infty } \left( 1 - ({e^{\rho ^2}})^{-X/b} \right) \\&= \lim _{b\rightarrow \infty } \left( 1 - e^{\mathrm{log}_e\left( ({e^{\rho ^2}})^{-X/b} \right)} \right) \\&= \lim _{b\rightarrow \infty } \left( 1 - e^{-X\mathrm{log}_e({e^{\rho ^2}})/b } \right) \\&= \lim _{b\rightarrow \infty } \left( 1 - e^{-X\rho ^2/b } \right)
\end{align*}

It is well known that:

\begin{align*}
\lim _{X\rightarrow 0}\frac{e^{X}-1}{X}=1
\end{align*}

It follows that $1 - e^{X}\approx -X$ as $X\rightarrow 0$, and so:

\begin{align*}
\lim _{b\rightarrow \infty } \varphi (X,\rho ,b) &= \lim _{b\rightarrow \infty } \left( 1 - e^{-X\rho ^2/b } \right) \\&= \lim _{{-X\rho ^2/b }\rightarrow 0} \left( 1 - e^{-X\rho ^2/b } \right) \\&= X\rho ^2/b
\end{align*}

Now consider the full warping function for positive $\rho$:

\begin{align*}
\lim _{b\rightarrow {}\infty }\omega (X,\rho ,b) &= \lim _{b\rightarrow {}\infty }\left(X+ \varphi (X,\rho ,b)(b - X)\right) \\&= X+ \lim _{b\rightarrow {}\infty }\varphi (X,\rho ,b)\lim _{b\rightarrow {}\infty }(b - X) \\&= X+ \left(X\rho ^2/b\right)\lim _{b\rightarrow {}\infty }(b - X) \\&= X+ \left(X\rho ^2/b\right)b \\&= X+ X\rho ^2 \\&= X(1 + \rho ^2)
\end{align*}

Next, consider the full warping function for negative $\rho$:

\begin{align*}
\lim _{b\rightarrow {}\infty }\omega (X,\rho ,b) &= \lim _{b\rightarrow {}\infty }\left(X- \varphi (b - X,\rho ,b)X\right) \\&= X- \lim _{b\rightarrow {}\infty }\varphi (b - X,\rho ,b)\lim _{b\rightarrow {}\infty }X\\&= X\left(1 - \lim _{b\rightarrow {}\infty }\varphi (b - X,\rho ,b)\right)
\end{align*}

The original reasoning about the warping ratio’s behavior as the bound approaches infinity implies that:

\begin{align*}
\lim _{b\rightarrow \infty } \varphi (b - X,\rho ,b) &= \lim _{b\rightarrow \infty } \left( 1 - e^{(X-b)\rho ^2/b } \right) \\&= \lim _{b\rightarrow \infty } \left( 1 - e^{(X/b-1)\rho ^2} \right) \\&= 1 - e^{-\rho ^2}
\end{align*}

And so, finally:

\begin{align*}
\lim _{b\rightarrow {}\infty }\omega (X,\rho ,b) &= X\left(1 - \lim _{b\rightarrow {}\infty }\varphi (b - X,\rho ,b)\right) \\&= Xe^{-\rho ^2}
\end{align*}

### 4.4 Kernel convolution solution

Equation A.16 gives the likelihood of the observed data, given the predicted kernel rate and estimated gamma dispersion. This likelihood is given by integration over a Poisson-gamma convolution:

\begin{eqnarray*}
\ell (y\, |\, \Psi ,\widetilde{{\sigma _{\gamma }^2}}) = \int _0^\infty \mathrm{Pois}(\mathrm{log}_e(y+ 1)\, |\, \Lambda )\mathrm{Gam}(\Lambda \, |\, \Psi ,\widetilde{{\sigma _{\gamma }^2}})d\Lambda
\end{eqnarray*}

This integral can be solved analytically. Note that the above equation parameterizes the gamma density function in terms of its expected value and variance. In order to use the standard formula for gamma density, these need to be converted to the standard parameterization in terms of “rate” ($\mathrm{rt}$) and “shape” ($\mathrm{sp}$), by setting $\gamma _{\mathrm{rt}}= \lambda /{\sigma _{\gamma }^2}$ and $\gamma _{\mathrm{sp}}= \lambda ^2/{\sigma _{\gamma }^2}$. Using standard formulae for the Poisson and gamma density functions and using the symbol “$\Gamma$” for the gamma function:

\begin{eqnarray*}
&&\int _0^\infty \mathrm{Pois}(Y\, |\, X)\mathrm{Gam}(X\, |\, \gamma _{\mathrm{rt}},{\gamma _{\mathrm{sp}}})dX\\&=& \int _0^\infty \frac{ X^Ye^{-X} }{ Y! } \frac{ \gamma _{\mathrm{rt}}^{\gamma _{\mathrm{sp}}}X^{{\gamma _{\mathrm{sp}}}-1}e^{-\gamma _{\mathrm{rt}}X} }{ \Gamma (\gamma _{\mathrm{sp}}) } dX\\&=& \frac{ \gamma _{\mathrm{rt}}^{\gamma _{\mathrm{sp}}} }{ Y!\Gamma (\gamma _{\mathrm{sp}}) } \int _0^\infty X^{Y+\gamma _{\mathrm{sp}}-1} e^{-X(1+\gamma _{\mathrm{rt}})} dX\\&=& \frac{ \gamma _{\mathrm{rt}}^{\gamma _{\mathrm{sp}}}\Gamma (Y+\gamma _{\mathrm{sp}}) }{ Y!\Gamma (\gamma _{\mathrm{sp}})(\gamma _{\mathrm{rt}}+1)^{Y+\gamma _{\mathrm{sp}}} } \\
\end{eqnarray*}

For generalization to the reals, wispack replaces $Y!$ with $\Gamma (Y+1)$. For numerical stability, the difference between the sum of the natural log of the numerator terms and the sum of the natural log of the denominator terms is computed first, before returning the exponential of this difference.

### 4.5 The rate-boundary theorem

**Theorem:** $\Psi > 0$ if and only if $r_i> 0$, for all rate components $r_i$ used in the calculation of $\Psi$.

**Proof:** The left-to-right direction of the biconditional follows immediately from the sigmoid rate theorem (Appendix 4.1). The right-to-left direction does not also immediately follow because the sigmoid rate theorem does not constrain what happens to $\Psi$ near the transition points $p$. So, consider the case when $x\approx p_{i^\prime }$. In this case:

\begin{eqnarray*}
1 + e^{-s_{i}(x- p_{i})} \approx \left\lbrace \begin{array}{@{}l@{\quad }l@{}}1 &\text{if } p_{i} < p_{i^\prime } \\2 &\text{if } p_{i} = p_{i^\prime } \\\infty &\text{if } p_{i} > p_{i^\prime } \end{array}\right.
\end{eqnarray*}

Thus, equation 10 can be rewritten as:

\begin{eqnarray*}
\Psi _{d}(x,r,s,p) && \approx r_1 + \left( \sum _{i=1}^{i^\prime -1} \frac{ r_{i+1} - r_i}{ 1 }\right) + \frac{ r_{{i^\prime }+1} - r_{i^\prime } }{ 2 } \\&& \approx r_{i^\prime } + \frac{ r_{{i^\prime }+1} - r_{i^\prime } }{ 2 } \\&& \approx \frac{ r_{{i^\prime }+1} + r_{i^\prime } }{ 2 } \\
\end{eqnarray*}

Assuming that all rate components $r_i> 0$, it follows immediately that $\Psi _{d}> 0$. Indeed, as expected, when near a transition point, $\Psi _{d}$ is approximately the mean of rates of the blocks immediately before and after that point.
