# Supplementary material for: Logistic regression for estimating functional effects with spatial transcriptomics
Source: Nucleic Acids Res. 2026 May 15;54(9):gkag466. doi: 10.1093/nar/gkag466 (PMC13176786; doi:10.1093/nar/gkag466)
Supplement: gkag466_Supplemental_File [file gkag466_supplemental_file.pdf]

Supplemental Material for "Logistic regression for estimating functional effects with spatial transcriptomics"

Michael Barkasi, Cody Nhan Pham, Demetrios Neophytou, Hysell V. Oviedo

February 15, 2026

Table S1: Parameter estimates for MERFISH cortex model.

parameter	estimate	CI.low	CI.high	p.value	p.value.adj	alpha.adj	sig
baseline_GABA_Rt_BCL11B_Tns/Blk1	2.8195301	2.6829598	3.0704716	NA	NA	NA	
beta_Rt_GABA_BCL11B_right_X_Tns/Blk1	0.5054260	-0.1925320	0.9623859	0.0015014	0.4323892	0.0001736	ns
beta_Rt_GABA_BCL11B_18_X_Tns/Blk1	1.4482024	0.9229194	1.8157278	0.0000000	0.0000000	0.0001235	***
beta_Rt_GABA_BCL11B_right18_X_Tns/Blk1	-0.2376868	-0.7184703	0.6365474	0.2585327	32.0580522	0.0004032	ns
baseline_GABA_Rt_BCL11B_Tns/Blk2	2.1447050	2.1047200	2.2701129	NA	NA	NA	
beta_Rt_GABA_BCL11B_right_X_Tns/Blk2	-0.0066565	-0.1181779	0.1389748	0.8804924	15.8488640	0.0027778	ns
beta_Rt_GABA_BCL11B_18_X_Tns/Blk2	0.1642594	0.0306518	0.3695174	0.0003003	0.0933840	0.0001608	ns
beta_Rt_GABA_BCL11B_right18_X_Tns/Blk2	0.0273375	-0.1836642	0.1928259	0.6465819	37.5017516	0.0008621	ns
baseline_GABA_Rt_CUX2_Tns/Blk1	1.0261534	1.0566853	1.2281960	NA	NA	NA	
beta_Rt_GABA_CUX2_right_X_Tns/Blk1	0.0635444	-0.2150046	0.2788161	0.3535182	37.8264438	0.0004673	ns
beta_Rt_GABA_CUX2_18_X_Tns/Blk1	0.2601694	0.0009581	0.4690194	0.0000000	0.0000000	0.0001238	***
beta_Rt_GABA_CUX2_right18_X_Tns/Blk1	0.0137257	-0.2278981	0.3788367	0.8936042	14.2976679	0.0031250	ns
baseline_GABA_Rt_CUX2_Tns/Blk2	1.1807639	1.0396814	1.6772184	NA	NA	NA	
beta_Rt_GABA_CUX2_right_X_Tns/Blk2	0.0456657	-0.7068178	0.7891727	0.8246422	21.4406966	0.0019231	ns
beta_Rt_GABA_CUX2_18_X_Tns/Blk2	0.5304809	-0.7386293	1.1252482	0.0130117	3.0187168	0.0002155	ns
beta_Rt_GABA_CUX2_right18_X_Tns/Blk2	-0.3515954	-1.3295524	0.8850431	0.1731558	25.8002202	0.0003356	ns
baseline_GABA_Rt_CUX2_Tns/Blk3	1.1889980	1.1940390	1.4616203	NA	NA	NA	
beta_Rt_GABA_CUX2_right_X_Tns/Blk3	0.1002944	-0.3331673	0.3765507	0.3108798	35.7511761	0.0004348	ns
beta_Rt_GABA_CUX2_18_X_Tns/Blk3	0.3106829	-0.1302061	0.6489616	0.0026023	0.7182464	0.0001812	ns
beta_Rt_GABA_CUX2_right18_X_Tns/Blk3	-0.0418173	-0.4700352	0.4184949	0.7538785	27.8935042	0.0013514	ns
baseline_GABA_Rt_CUX2_Tns/Blk4	1.3668507	1.2539513	1.6085012	NA	NA	NA	
beta_Rt_GABA_CUX2_right_X_Tns/Blk4	-0.1470410	-0.5707199	0.4292519	0.3080773	36.0450405	0.0004274	ns
beta_Rt_GABA_CUX2_18_X_Tns/Blk4	0.2946824	-0.1761711	0.7512294	0.0168151	3.6825143	0.0002283	ns
beta_Rt_GABA_CUX2_right18_X_Tns/Blk4	-0.0792391	-0.7288066	0.4395690	0.6648984	37.2343109	0.0008929	ns
baseline_GABA_Rt_CUX2_Tns/Blk5	1.5033258	1.4104866	1.6882960	NA	NA	NA	
beta_Rt_GABA_CUX2_right_X_Tns/Blk5	-0.3097366	-0.6592707	0.3101677	0.0189170	4.0860775	0.0002315	ns
beta_Rt_GABA_CUX2_18_X_Tns/Blk5	-0.0943877	-0.4972223	0.4036043	0.4371935	39.3474127	0.0005556	ns
beta_Rt_GABA_CUX2_right18_X_Tns/Blk5	1.0113832	0.0143865	1.5128120	0.0000000	0.0000000	0.0001241	***
baseline_GABA_Rt_CUX2_Tns/Blk6	1.3573149	1.3404511	1.5394962	NA	NA	NA	
beta_Rt_GABA_CUX2_right_X_Tns/Blk6	0.1456781	-0.1210766	0.3538258	0.0233210	4.9673706	0.0002347	ns
beta_Rt_GABA_CUX2_18_X_Tns/Blk6	0.6391522	0.3282621	0.8399882	0.0000000	0.0000000	0.0001244	***
beta_Rt_GABA_CUX2_right18_X_Tns/Blk6	-0.3419530	-0.6367331	0.0824029	0.0006005	0.1813632	0.0001656	ns
baseline_GABA_Rt_FEZF2_Tns/Blk1	1.3296296	1.3236053	2.0324247	NA	NA	NA	
beta_Rt_GABA_FEZF2_right_X_Tns/Blk1	0.0011281	-0.3345561	0.6407314	0.9945951	1.9891903	0.0250000	ns
beta_Rt_GABA_FEZF2_18_X_Tns/Blk1	0.1824554	-0.4474186	0.8991309	0.3507156	37.8772896	0.0004630	ns
beta_Rt_GABA_FEZF2_right18_X_Tns/Blk1	-0.0598040	-1.0798720	0.7366949	0.8237414	23.0647583	0.0017857	ns
baseline_GABA_Rt_FEZF2_Tns/Blk2	1.4335121	1.4631346	2.2023286	NA	NA	NA	
beta_Rt_GABA_FEZF2_right_X_Tns/Blk2	0.1055075	-0.4115951	0.4273740	0.3681313	37.9175258	0.0004854	ns
beta_Rt_GABA_FEZF2_18_X_Tns/Blk2	-0.1207688	-0.5255293	0.3497182	0.2786508	33.9953959	0.0004098	ns
beta_Rt_GABA_FEZF2_right18_X_Tns/Blk2	-0.0327218	-0.4403884	0.5168432	0.8252427	20.6310680	0.0020000	ns
baseline_GABA_Rt_FEZF2_Tns/Blk3	1.6818856	1.6830901	2.4510950	NA	NA	NA	
beta_Rt_GABA_FEZF2_right_X_Tns/Blk3	-0.2089180	-0.7754500	0.4046745	0.1772595	26.2344110	0.0003378	ns

Continued on next page

parameter	estimate	CI.low	CI.high	p.value	p.value.adj	alpha.adj	sig
beta_Rt_GABA_FEZF2_18_X_Tns/Blk3	-0.0845241	-0.6002127	0.3352768	0.5364828	38.6267641	0.0006944	ns
beta_Rt_GABA_FEZF2_right18_X_Tns/Blk3	0.1422716	-0.5391043	0.8191489	0.4550095	40.0408368	0.0005682	ns
baseline_GABA_Rt_FEZF2_Tns/Blk4	1.4797930	1.5180067	2.2310426	NA	NA	NA	
beta_Rt_GABA_FEZF2_right_X_Tns/Blk4	0.2011664	-0.2491831	0.8052325	0.1483335	22.8433590	0.0003247	ns
beta_Rt_GABA_FEZF2_18_X_Tns/Blk4	0.2389944	-0.2731519	0.6579237	0.0609549	11.1547393	0.0002732	ns
beta_Rt_GABA_FEZF2_right18_X_Tns/Blk4	0.0654803	-0.7745112	0.6522996	0.7651887	26.7816034	0.0014286	ns
baseline_GABA_Rt_FEZF2_Tns/Blk5	1.2625320	1.1778421	2.3116524	NA	NA	NA	
beta_Rt_GABA_FEZF2_right_X_Tns/Blk5	0.1020613	-1.2431192	0.6854820	0.7421680	29.6867180	0.0012500	ns
beta_Rt_GABA_FEZF2_18_X_Tns/Blk5	0.0128362	-0.9113908	0.5793061	0.9731759	4.8658793	0.0100000	ns
beta_Rt_GABA_FEZF2_right18_X_Tns/Blk5	0.0766936	-0.7357197	1.3324375	0.8054249	23.3573216	0.0017241	ns
baseline_GABA_Rt_FEZF2_Tns/Blk6	1.2356855	1.2810620	2.0333595	NA	NA	NA	
beta_Rt_GABA_FEZF2_right_X_Tns/Blk6	-0.0149581	-0.5176955	0.3648521	0.9126214	12.7766990	0.0035714	ns
beta_Rt_GABA_FEZF2_18_X_Tns/Blk6	0.0880208	-0.6513955	1.0549446	0.6587929	37.5511961	0.0008772	ns
beta_Rt_GABA_FEZF2_right18_X_Tns/Blk6	-0.0893642	-1.0553310	0.7940417	0.6988289	34.9414473	0.0010000	ns
baseline_GABA_Rt_NXPH3_Tns/Blk1	1.6077003	1.5706988	1.9086609	NA	NA	NA	
beta_Rt_GABA_NXPH3_right_X_Tns/Blk1	0.0401729	-0.5062920	0.6514890	0.8614753	16.3680312	0.0026316	ns
beta_Rt_GABA_NXPH3_18_X_Tns/Blk1	0.4612022	-0.2174626	0.9819908	0.0088079	2.1491342	0.0002049	ns
beta_Rt_GABA_NXPH3_right18_X_Tns/Blk1	0.1116219	-0.8787840	0.9236777	0.6893204	37.9126214	0.0009091	ns
baseline_GABA_Rt_NXPH3_Tns/Blk2	1.7101172	1.6941353	1.9633457	NA	NA	NA	
beta_Rt_GABA_NXPH3_right_X_Tns/Blk2	0.0023357	-0.1666109	0.1601424	0.9681714	6.7771995	0.0071429	ns
beta_Rt_GABA_NXPH3_18_X_Tns/Blk2	0.1709564	-0.0192443	0.4098401	0.0050045	1.2911620	0.0001938	ns
beta_Rt_GABA_NXPH3_right18_X_Tns/Blk2	0.0719150	-0.2579903	0.3604267	0.4029627	38.2814533	0.0005263	ns
baseline_GABA_Rt_NXPH3_Tns/Blk3	1.4784139	1.5075049	1.7772711	NA	NA	NA	
beta_Rt_GABA_NXPH3_right_X_Tns/Blk3	-0.1340701	-0.3689557	0.0674852	0.0337304	6.8472625	0.0002463	ns
beta_Rt_GABA_NXPH3_18_X_Tns/Blk3	-0.0134258	-0.2161946	0.2079525	0.8378541	20.1084976	0.0020833	ns
beta_Rt_GABA_NXPH3_right18_X_Tns/Blk3	0.2278569	-0.1414875	0.5719192	0.0158142	3.5740166	0.0002212	ns
baseline_GABA_Rt_RORB_Tns/Blk1	1.1533011	1.0738135	1.4862814	NA	NA	NA	
beta_Rt_GABA_RORB_right_X_Tns/Blk1	-0.0623515	-0.4903746	0.4795328	0.6246622	38.1043940	0.0008197	ns
beta_Rt_GABA_RORB_18_X_Tns/Blk1	0.1814440	-0.3706272	0.7354653	0.1890702	26.8479632	0.0003521	ns
beta_Rt_GABA_RORB_right18_X_Tns/Blk1	0.3432421	-0.4275111	1.0159261	0.0553498	10.2950656	0.0002688	ns
baseline_GABA_Rt_RORB_Tns/Blk2	0.8437766	0.8873580	1.1981389	NA	NA	NA	
beta_Rt_GABA_RORB_right_X_Tns/Blk2	0.2271103	-0.4400831	0.6034221	0.0602542	11.0867781	0.0002717	ns
beta_Rt_GABA_RORB_18_X_Tns/Blk2	0.4107413	-0.3290771	1.1598831	0.0215194	4.6266640	0.0002326	ns
beta_Rt_GABA_RORB_right18_X_Tns/Blk2	-0.4332198	-1.3242692	0.3626690	0.0393354	7.7490742	0.0002538	ns
baseline_GABA_Rt_RORB_Tns/Blk3	1.1385643	1.0665442	1.4709061	NA	NA	NA	
beta_Rt_GABA_RORB_right_X_Tns/Blk3	-0.0030870	-0.2928041	0.3856774	0.9815834	3.9263337	0.0125000	ns
beta_Rt_GABA_RORB_18_X_Tns/Blk3	-0.0409604	-0.3732937	0.3178910	0.7087379	34.0194175	0.0010417	ns
beta_Rt_GABA_RORB_right18_X_Tns/Blk3	-0.0532617	-0.5873160	0.4047747	0.7273546	32.7309579	0.0011111	ns
baseline_GABA_Rt_RORB_Tns/Blk4	1.1107275	1.2071577	1.3952487	NA	NA	NA	
beta_Rt_GABA_RORB_right_X_Tns/Blk4	0.1032258	-0.1986624	0.3560244	0.2113903	28.5376839	0.0003704	ns
beta_Rt_GABA_RORB_18_X_Tns/Blk4	0.1497881	-0.1746147	0.4640428	0.0821740	13.9695726	0.0002941	ns
beta_Rt_GABA_RORB_right18_X_Tns/Blk4	-0.1124873	-0.5322495	0.3588180	0.3463117	37.7479732	0.0004587	ns
baseline_GABA_Rt_RORB_Tns/Blk5	1.2556335	1.2943126	1.4912771	NA	NA	NA	
beta_Rt_GABA_RORB_right_X_Tns/Blk5	-0.1604802	-0.3277687	0.1368997	0.0104094	2.4670203	0.0002110	ns
beta_Rt_GABA_RORB_18_X_Tns/Blk5	-0.0554919	-0.2217666	0.2280303	0.3914523	39.1452307	0.0005000	ns
beta_Rt_GABA_RORB_right18_X_Tns/Blk5	0.3013954	-0.1287790	0.5626709	0.0019017	0.5419878	0.0001754	ns
baseline_GABA_Rt_RORB_Tns/Blk6	1.0673655	1.1084859	1.3653438	NA	NA	NA	
beta_Rt_GABA_RORB_right_X_Tns/Blk6	0.1964233	-0.1597003	0.5715334	0.0475428	8.9855870	0.0002646	ns
beta_Rt_GABA_RORB_18_X_Tns/Blk6	0.1384924	-0.2007506	0.4279097	0.1020919	16.8451606	0.0003030	ns
beta_Rt_GABA_RORB_right18_X_Tns/Blk6	-0.1581418	-0.6545137	0.3102614	0.2188970	29.1133020	0.0003759	ns
baseline_GABA_Rt_SATB2_Tns/Blk1	1.4725987	1.4839000	1.8162795	NA	NA	NA	
beta_Rt_GABA_SATB2_right_X_Tns/Blk1	0.1131692	-0.6326304	0.6280024	0.6400761	37.7644880	0.0008475	ns
beta_Rt_GABA_SATB2_18_X_Tns/Blk1	0.1537410	-0.5768404	0.6261492	0.4918427	38.8555700	0.0006329	ns
beta_Rt_GABA_SATB2_right18_X_Tns/Blk1	-0.3670711	-1.0555909	0.9066344	0.2326093	30.2392153	0.0003846	ns
baseline_GABA_Rt_SATB2_Tns/Blk2	1.8396813	1.5625268	2.0496154	NA	NA	NA	
beta_Rt_GABA_SATB2_right_X_Tns/Blk2	-0.3843806	-0.7928144	0.3206374	0.0160144	3.5712141	0.0002242	ns
beta_Rt_GABA_SATB2_18_X_Tns/Blk2	-0.4658241	-0.8438884	0.5317252	0.0182164	3.9711741	0.0002294	ns
beta_Rt_GABA_SATB2_right18_X_Tns/Blk2	0.6522568	-0.5026794	1.2498425	0.0118106	2.7518767	0.0002146	ns
baseline_GABA_Rt_SATB2_Tns/Blk3	1.9920246	1.8585312	2.2515324	NA	NA	NA	

Continued on next page

parameter	estimate	CI.low	CI.high	p.value	p.value.adj	alpha.adj	sig
beta_Rt_GABA_SATB2_right_X_Tns/Blk3	0.0471731	-0.5914811	0.4558024	0.7856070	25.1394255	0.0015625	ns
beta_Rt_GABA_SATB2_18_X_Tns/Blk3	-0.2358596	-0.6589341	0.3964047	0.0718647	12.6481834	0.0002841	ns
beta_Rt_GABA_SATB2_right18_X_Tns/Blk3	-0.1484245	-0.8333081	0.6279931	0.4699229	39.4735262	0.0005952	ns
baseline_GABA_Rt_SATB2_Tns/Blk4	1.8664756	1.7596113	2.0093973	NA	NA	NA	
beta_Rt_GABA_SATB2_right_X_Tns/Blk4	0.0045875	-0.1827652	0.3316907	0.9610650	8.6495846	0.0055556	ns
beta_Rt_GABA_SATB2_18_X_Tns/Blk4	0.2853461	0.0564939	0.6906743	0.0009008	0.2657392	0.0001695	ns
beta_Rt_GABA_SATB2_right18_X_Tns/Blk4	0.0426500	-0.5423724	0.2692533	0.7245521	33.3293965	0.0010870	ns
baseline_GABA_Rt_SATB2_Tns/Blk5	1.5080116	1.5129541	1.8464447	NA	NA	NA	
beta_Rt_GABA_SATB2_right_X_Tns/Blk5	0.0675310	-0.3897325	0.3671659	0.5059554	38.9585627	0.0006494	ns
beta_Rt_GABA_SATB2_18_X_Tns/Blk5	0.5885825	-0.2181993	1.0316191	0.0009008	0.2675408	0.0001684	ns
beta_Rt_GABA_SATB2_right18_X_Tns/Blk5	-0.2977345	-0.8998222	0.6427016	0.1851666	26.6639976	0.0003472	ns
baseline_GABA_Rt_SATB2_Tns/Blk6	1.7137057	1.6805702	1.8764357	NA	NA	NA	
beta_Rt_GABA_SATB2_right_X_Tns/Blk6	0.2567348	-0.0616373	0.5094479	0.0015014	0.4338905	0.0001730	ns
beta_Rt_GABA_SATB2_18_X_Tns/Blk6	0.1801487	-0.0641012	0.4499735	0.0102092	2.4399960	0.0002092	ns
beta_Rt_GABA_SATB2_right18_X_Tns/Blk6	-0.2455804	-0.6487460	0.1972909	0.0411370	7.9805825	0.0002577	ns
baseline_Glut_Rt_BCL11B_Tns/Blk1	2.1935955	1.9749994	2.4547426	NA	NA	NA	
beta_Rt_Glut_BCL11B_right_X_Tns/Blk1	-0.3276586	-1.0530939	0.1689859	0.0375338	7.4692223	0.0002513	ns
beta_Rt_Glut_BCL11B_18_X_Tns/Blk1	-0.0323118	-0.5336975	0.4282206	0.8399560	19.3189871	0.0021739	ns
beta_Rt_Glut_BCL11B_right18_X_Tns/Blk1	0.0713536	-0.4850199	0.9026790	0.7443699	29.0304274	0.0012821	ns
baseline_Glut_Rt_BCL11B_Tns/Blk2	2.7762724	2.6497800	2.7797287	NA	NA	NA	
beta_Rt_Glut_BCL11B_right_X_Tns/Blk2	0.0127486	-0.1009077	0.1892296	0.7794015	26.4996497	0.0014706	ns
beta_Rt_Glut_BCL11B_18_X_Tns/Blk2	0.5440577	0.3499394	0.7114918	0.0000000	0.0000000	0.0001247	***
beta_Rt_Glut_BCL11B_right18_X_Tns/Blk2	0.0852183	-0.2059892	0.3087107	0.2807527	33.9710740	0.0004132	ns
baseline_Glut_Rt_BCL11B_Tns/Blk3	1.4694747	1.3607454	1.6406448	NA	NA	NA	
beta_Rt_Glut_BCL11B_right_X_Tns/Blk3	0.1628272	-0.2012092	0.4892987	0.1204084	19.1449304	0.0003145	ns
beta_Rt_Glut_BCL11B_18_X_Tns/Blk3	0.5103942	0.0754773	0.8030824	0.0000000	0.0000000	0.0001250	***
beta_Rt_Glut_BCL11B_right18_X_Tns/Blk3	-0.5454118	-1.0864618	0.1147843	0.0011010	0.3236913	0.0001701	ns
baseline_Glut_Rt_BCL11B_Tns/Blk4	1.4529922	1.3159316	1.5709826	NA	NA	NA	
beta_Rt_Glut_BCL11B_right_X_Tns/Blk4	0.2571698	-0.0637198	0.6478020	0.0044040	1.1538385	0.0001908	ns
beta_Rt_Glut_BCL11B_18_X_Tns/Blk4	0.4712298	0.0426059	0.7777277	0.0000000	0.0000000	0.0001253	***
beta_Rt_Glut_BCL11B_right18_X_Tns/Blk4	-0.4115236	-0.9585655	0.0645133	0.0026023	0.7156441	0.0001818	ns
baseline_Glut_Rt_CUX2_Tns/Blk1	1.6436645	1.6083699	1.7420309	NA	NA	NA	
beta_Rt_Glut_CUX2_right_X_Tns/Blk1	0.1344790	-0.0533224	0.2783108	0.0048043	1.2491242	0.0001923	ns
beta_Rt_Glut_CUX2_18_X_Tns/Blk1	0.4174309	0.1625226	0.5491625	0.0000000	0.0000000	0.0001256	***
beta_Rt_Glut_CUX2_right18_X_Tns/Blk1	-0.0649062	-0.2965506	0.1907965	0.3740366	38.1517366	0.0004902	ns
baseline_Glut_Rt_CUX2_Tns/Blk2	3.6979865	3.4418038	3.9430360	NA	NA	NA	
beta_Rt_Glut_CUX2_right_X_Tns/Blk2	-0.3800113	-0.9330770	0.2491141	0.0155140	3.5526974	0.0002183	ns
beta_Rt_Glut_CUX2_18_X_Tns/Blk2	0.5492928	0.0065822	1.1837109	0.0008007	0.2386148	0.0001678	ns
beta_Rt_Glut_CUX2_right18_X_Tns/Blk2	0.4076690	-0.6110906	0.9067013	0.0425383	8.2098889	0.0002591	ns
baseline_Glut_Rt_CUX2_Tns/Blk3	3.8705061	3.7388951	3.8742085	NA	NA	NA	
beta_Rt_Glut_CUX2_right_X_Tns/Blk3	-0.0622719	-0.2473486	0.0781984	0.1796617	26.4102692	0.0003401	ns
beta_Rt_Glut_CUX2_18_X_Tns/Blk3	0.9553937	0.7907913	1.1374982	0.0000000	0.0000000	0.0001259	***
beta_Rt_Glut_CUX2_right18_X_Tns/Blk3	-0.0731054	-0.2461263	0.1296898	0.1886698	26.9797818	0.0003497	ns
baseline_Glut_Rt_CUX2_Tns/Blk4	3.3999085	3.1844823	3.4571880	NA	NA	NA	
beta_Rt_Glut_CUX2_right_X_Tns/Blk4	0.2203696	-0.0945777	0.5422675	0.0166150	3.6552898	0.0002273	ns
beta_Rt_Glut_CUX2_18_X_Tns/Blk4	1.2743217	0.9984091	1.5896268	0.0000000	0.0000000	0.0001263	***
beta_Rt_Glut_CUX2_right18_X_Tns/Blk4	-0.6863882	-1.0471060	-0.2765430	0.0000000	0.0000000	0.0001266	***
baseline_Glut_Rt_FEZF2_Tns/Blk1	2.9629409	2.8155311	2.9785485	NA	NA	NA	
beta_Rt_Glut_FEZF2_right_X_Tns/Blk1	-0.0946582	-0.3447239	0.1617503	0.1535382	23.4913422	0.0003268	ns
beta_Rt_Glut_FEZF2_18_X_Tns/Blk1	-0.1801618	-0.5291968	0.1707059	0.0751677	13.0040036	0.0002890	ns
beta_Rt_Glut_FEZF2_right18_X_Tns/Blk1	-0.0899547	-0.5785508	0.4341312	0.5584026	39.6465819	0.0007042	ns
baseline_Glut_Rt_FEZF2_Tns/Blk2	3.2177995	3.0002888	3.3546059	NA	NA	NA	
beta_Rt_Glut_FEZF2_right_X_Tns/Blk2	-0.0564055	-0.3716982	0.2618078	0.5183665	38.8774897	0.0006667	ns
beta_Rt_Glut_FEZF2_18_X_Tns/Blk2	0.6930899	0.2865350	1.1619088	0.0000000	0.0000000	0.0001269	***
beta_Rt_Glut_FEZF2_right18_X_Tns/Blk2	0.0511411	-0.4643510	0.5168773	0.7356621	30.1621459	0.0012195	ns
baseline_Glut_Rt_FEZF2_Tns/Blk3	1.5547373	1.3290924	2.1612609	NA	NA	NA	
beta_Rt_Glut_FEZF2_right_X_Tns/Blk3	1.0088722	-0.4309847	1.5958522	0.0002002	0.0632569	0.0001582	ns
beta_Rt_Glut_FEZF2_18_X_Tns/Blk3	0.8771851	-0.4853015	1.5072534	0.0022020	0.6187569	0.0001779	ns
beta_Rt_Glut_FEZF2_right18_X_Tns/Blk3	-0.7780823	-1.7115182	0.8332750	0.0255230	5.3853468	0.0002370	ns

Continued on next page

parameter	estimate	CI.low	CI.high	p.value	p.value.adj	alpha.adj	sig
baseline_Glut_Rt_FEZF2_Tns/Blk4	1.1513069	1.1685426	1.3707260	NA	NA	NA	
beta_Rt_Glut_FEZF2_right_X_Tns/Blk4	0.1224343	-0.2001732	0.3550603	0.1038935	16.9346412	0.0003067	ns
beta_Rt_Glut_FEZF2_18_X_Tns/Blk4	0.2013515	-0.1465923	0.4085940	0.0080072	1.9857872	0.0002016	ns
beta_Rt_Glut_FEZF2_right18_X_Tns/Blk4	-0.1192186	-0.4228135	0.2910400	0.2568311	32.1038935	0.0004000	ns
baseline_Glut_Rt_NXPH3_Tns/Blk1	4.5107104	4.3324371	4.5563729	NA	NA	NA	
beta_Rt_Glut_NXPH3_right_X_Tns/Blk1	0.1842539	-0.0522297	0.3966551	0.0025023	0.6931238	0.0001805	ns
beta_Rt_Glut_NXPH3_18_X_Tns/Blk1	0.2443917	-0.1187912	0.5189324	0.0042038	1.1055950	0.0001901	ns
beta_Rt_Glut_NXPH3_right18_X_Tns/Blk1	-0.1717531	-0.7783298	0.1188614	0.0885797	14.7928135	0.0002994	ns
baseline_Glut_Rt_NXPH3_Tns/Blk2	3.5677735	3.3622569	3.7426455	NA	NA	NA	
beta_Rt_Glut_NXPH3_right_X_Tns/Blk2	0.1323362	-0.2943859	1.3074449	0.3925533	38.8627765	0.0005051	ns
beta_Rt_Glut_NXPH3_18_X_Tns/Blk2	0.8284155	0.3312164	1.3916426	0.0000000	0.0000000	0.0001272	***
beta_Rt_Glut_NXPH3_right18_X_Tns/Blk2	-0.3265706	-1.3857879	0.5869458	0.1098989	17.6937244	0.0003106	ns
baseline_Glut_Rt_NXPH3_Tns/Blk3	3.2910316	2.9020029	3.3100673	NA	NA	NA	
beta_Rt_Glut_NXPH3_right_X_Tns/Blk3	-0.1225484	-1.1276396	0.4661965	0.4780302	39.1984786	0.0006098	ns
beta_Rt_Glut_NXPH3_18_X_Tns/Blk3	0.5954773	0.0372335	1.1635986	0.0002002	0.0630568	0.0001587	ns
beta_Rt_Glut_NXPH3_right18_X_Tns/Blk3	0.1728384	-0.7843626	1.1911583	0.4645181	39.4840356	0.0005882	ns
baseline_Glut_Rt_NXPH3_Tns/Blk4	1.8733297	1.6432396	2.1568336	NA	NA	NA	
beta_Rt_Glut_NXPH3_right_X_Tns/Blk4	0.9243272	0.1739024	1.9963856	0.0003003	0.0936843	0.0001603	ns
beta_Rt_Glut_NXPH3_18_X_Tns/Blk4	2.5379919	1.5366691	3.2561533	0.0000000	0.0000000	0.0001276	***
beta_Rt_Glut_NXPH3_right18_X_Tns/Blk4	-1.7501303	-3.0952692	-0.6436025	0.0000000	0.0000000	0.0001279	***
baseline_Glut_Rt_NXPH3_Tns/Blk5	1.9596828	1.8091806	2.0108473	NA	NA	NA	
beta_Rt_Glut_NXPH3_right_X_Tns/Blk5	0.0692389	-0.1233278	0.3301576	0.2917626	35.0115104	0.0004167	ns
beta_Rt_Glut_NXPH3_18_X_Tns/Blk5	0.4532450	0.2237270	0.8201551	0.0000000	0.0000000	0.0001282	***
beta_Rt_Glut_NXPH3_right18_X_Tns/Blk5	-0.3171868	-0.7683415	0.1097154	0.0093084	2.2619357	0.0002058	ns
baseline_Glut_Rt_RORB_Tns/Blk1	1.7214600	1.5921143	1.8630494	NA	NA	NA	
beta_Rt_Glut_RORB_right_X_Tns/Blk1	0.2702357	-0.1133717	0.6383633	0.0103093	2.4536082	0.0002101	ns
beta_Rt_Glut_RORB_18_X_Tns/Blk1	-0.1577337	-0.4378810	0.3256305	0.1482334	22.9761786	0.0003226	ns
beta_Rt_Glut_RORB_right18_X_Tns/Blk1	-0.2985615	-0.9381943	0.1265438	0.0448404	8.6093484	0.0002604	ns
baseline_Glut_Rt_RORB_Tns/Blk2	2.2003880	2.1056117	2.2657101	NA	NA	NA	
beta_Rt_Glut_RORB_right_X_Tns/Blk2	0.3920735	0.1132753	0.5390163	0.0000000	0.0000000	0.0001285	***
beta_Rt_Glut_RORB_18_X_Tns/Blk2	0.5057493	0.1562223	0.7153569	0.0000000	0.0000000	0.0001289	***
beta_Rt_Glut_RORB_right18_X_Tns/Blk2	-0.4277056	-0.6962438	0.0250693	0.0000000	0.0000000	0.0001292	***
baseline_Glut_Rt_RORB_Tns/Blk3	3.7533412	3.6079213	3.8720414	NA	NA	NA	
beta_Rt_Glut_RORB_right_X_Tns/Blk3	0.2966947	-0.1027089	0.5438629	0.0012011	0.3519167	0.0001706	ns
beta_Rt_Glut_RORB_18_X_Tns/Blk3	0.9399965	0.6565578	1.5066641	0.0000000	0.0000000	0.0001295	***
beta_Rt_Glut_RORB_right18_X_Tns/Blk3	-0.2452655	-0.7939946	0.1414674	0.0563507	10.4248824	0.0002703	ns
baseline_Glut_Rt_RORB_Tns/Blk4	2.4375478	2.1948779	2.5051658	NA	NA	NA	
beta_Rt_Glut_RORB_right_X_Tns/Blk4	0.0977092	-0.3055977	0.5013836	0.4026624	38.6555900	0.0005208	ns
beta_Rt_Glut_RORB_18_X_Tns/Blk4	0.6419830	0.0981159	1.0595645	0.0000000	0.0000000	0.0001299	***
beta_Rt_Glut_RORB_right18_X_Tns/Blk4	-0.4678093	-1.1075026	0.1797191	0.0101091	2.4261836	0.0002083	ns
baseline_Glut_Rt_SATB2_Tns/Blk1	4.5675886	4.4999043	4.5834893	NA	NA	NA	
beta_Rt_Glut_SATB2_right_X_Tns/Blk1	0.0525241	-0.0598522	0.1380097	0.0477430	8.9756781	0.0002660	ns
beta_Rt_Glut_SATB2_18_X_Tns/Blk1	0.0926942	-0.0560451	0.2084169	0.0069062	1.7334601	0.0001992	ns
beta_Rt_Glut_SATB2_right18_X_Tns/Blk1	-0.0956642	-0.2277833	0.0687146	0.0156141	3.5443900	0.0002203	ns
baseline_Glut_Rt_SATB2_Tns/Blk2	4.4411793	4.2935508	4.6134453	NA	NA	NA	
beta_Rt_Glut_SATB2_right_X_Tns/Blk2	-0.0745789	-0.4742846	0.2302581	0.4290862	39.4759283	0.0005435	ns
beta_Rt_Glut_SATB2_18_X_Tns/Blk2	0.5812596	0.2062626	0.8788119	0.0000000	0.0000000	0.0001302	***
beta_Rt_Glut_SATB2_right18_X_Tns/Blk2	0.0387803	-0.3051657	0.4277318	0.7229507	33.9786808	0.0010638	ns
baseline_Glut_Rt_SATB2_Tns/Blk3	4.8648168	4.8194819	4.8952712	NA	NA	NA	
beta_Rt_Glut_SATB2_right_X_Tns/Blk3	-0.0052159	-0.0894491	0.0842677	0.8519668	17.8913022	0.0023810	ns
beta_Rt_Glut_SATB2_18_X_Tns/Blk3	0.4820575	0.3521527	0.5510889	0.0000000	0.0000000	0.0001305	***
beta_Rt_Glut_SATB2_right18_X_Tns/Blk3	-0.2514160	-0.3724241	-0.0982563	0.0000000	0.0000000	0.0001309	***
baseline_GABA_tslope_BCL11B_Tns/Blk1	1.3954290	0.7046353	3.1374179	NA	NA	NA	
beta_tslope_GABA_BCL11B_right_X_Tns/Blk1	2.2003764	-0.6613871	4.9943860	0.0035032	0.9353418	0.0001873	ns
beta_tslope_GABA_BCL11B_18_X_Tns/Blk1	-0.2047032	-2.2869942	1.8488578	0.6944250	36.8045241	0.0009434	ns
beta_tslope_GABA_BCL11B_right18_X_Tns/Blk1	-0.1144804	-3.1909893	1.8146598	0.9353418	10.2887599	0.0045455	ns
baseline_GABA_tslope_CUX2_Tns/Blk1	5.3630175	3.5842022	6.3348848	NA	NA	NA	
beta_tslope_GABA_CUX2_right_X_Tns/Blk1	1.5319688	-0.1806036	3.5367152	0.0022020	0.6165549	0.0001786	ns
beta_tslope_GABA_CUX2_18_X_Tns/Blk1	0.9260782	-0.3455136	2.7356432	0.0337304	6.8135322	0.0002475	ns

Continued on next page

parameter	estimate	CI.low	CI.high	p.value	p.value.adj	alpha.adj	sig
beta_tslope_GABA_CUX2_right18_X_Tns/Blk1	0.1155760	-1.6629072	2.1833920	0.8239415	22.2464218	0.0018519	ns
baseline_GABA_tslope_CUX2_Tns/Blk2	6.4211902	4.2481539	6.9127542	NA	NA	NA	
beta_tslope_GABA_CUX2_right_X_Tns/Blk2	1.9635877	-1.3323896	3.4949618	0.0031028	0.8408568	0.0001845	ns
beta_tslope_GABA_CUX2_18_X_Tns/Blk2	2.1020807	-0.6304214	3.8894547	0.0017015	0.4866380	0.0001748	ns
beta_tslope_GABA_CUX2_right18_X_Tns/Blk2	0.7356610	-2.0324981	2.6688650	0.4340907	39.5022520	0.0005495	ns
baseline_GABA_tslope_CUX2_Tns/Blk3	5.5791405	3.0503611	6.3709546	NA	NA	NA	
beta_tslope_GABA_CUX2_right_X_Tns/Blk3	1.6828428	-0.8671700	3.6213305	0.0335302	6.8736863	0.0002439	ns
beta_tslope_GABA_CUX2_18_X_Tns/Blk3	0.0502709	-0.9846978	2.8958468	0.9611650	7.6893204	0.0062500	ns
beta_tslope_GABA_CUX2_right18_X_Tns/Blk3	2.2307010	-1.8019249	3.4826504	0.0158142	3.5582024	0.0002222	ns
baseline_GABA_tslope_CUX2_Tns/Blk4	6.1783635	5.0298688	7.1116855	NA	NA	NA	
beta_tslope_GABA_CUX2_right_X_Tns/Blk4	1.2138786	-0.5565549	3.2100886	0.0083075	2.0436393	0.0002033	ns
beta_tslope_GABA_CUX2_18_X_Tns/Blk4	2.1928914	-0.9434482	4.0080363	0.0021019	0.5948354	0.0001767	ns
beta_tslope_GABA_CUX2_right18_X_Tns/Blk4	1.2553113	-1.1270353	2.8397450	0.0340306	6.8061255	0.0002500	ns
baseline_GABA_tslope_CUX2_Tns/Blk5	6.5643882	3.6101331	7.3503842	NA	NA	NA	
beta_tslope_GABA_CUX2_right_X_Tns/Blk5	1.8634176	-2.7201671	4.2516235	0.0318286	6.5885297	0.0002415	ns
beta_tslope_GABA_CUX2_18_X_Tns/Blk5	0.8799161	-3.1755715	4.0448149	0.2309078	30.2489240	0.0003817	ns
beta_tslope_GABA_CUX2_right18_X_Tns/Blk5	2.0342720	-0.8973639	3.8966902	0.0032029	0.8615754	0.0001859	ns
baseline_GABA_tslope_FEZF2_Tns/Blk1	4.8563449	3.7569442	5.9368547	NA	NA	NA	
beta_tslope_GABA_FEZF2_right_X_Tns/Blk1	0.4364971	-0.0565851	3.0395305	0.8051246	24.1537384	0.0016667	ns
beta_tslope_GABA_FEZF2_18_X_Tns/Blk1	1.4497228	0.0267416	2.7431331	0.0000000	0.0000000	0.0001312	***
beta_tslope_GABA_FEZF2_right18_X_Tns/Blk1	1.6137219	-1.3703770	2.7733288	0.0155140	3.5371835	0.0002193	ns
baseline_GABA_tslope_FEZF2_Tns/Blk2	4.3654299	3.0686062	5.9988239	NA	NA	NA	
beta_tslope_GABA_FEZF2_right_X_Tns/Blk2	2.2347463	-0.2151308	3.5564123	0.0000000	0.0000000	0.0001316	***
beta_tslope_GABA_FEZF2_18_X_Tns/Blk2	0.9638524	-0.7367885	2.4387069	0.0379341	7.5109599	0.0002525	ns
beta_tslope_GABA_FEZF2_right18_X_Tns/Blk2	1.4315629	-0.7072229	3.2865955	0.0329296	6.7835052	0.0002427	ns
baseline_GABA_tslope_FEZF2_Tns/Blk3	5.7888061	3.5921856	6.4609776	NA	NA	NA	
beta_tslope_GABA_FEZF2_right_X_Tns/Blk3	0.8287437	-0.7525208	2.5779903	0.0711640	12.5960364	0.0002825	ns
beta_tslope_GABA_FEZF2_18_X_Tns/Blk3	2.1453893	-1.3759214	3.8846319	0.0024022	0.6678010	0.0001799	ns
beta_tslope_GABA_FEZF2_right18_X_Tns/Blk3	1.6499991	-1.0706736	3.3336564	0.0153138	3.5221700	0.0002174	ns
baseline_GABA_tslope_FEZF2_Tns/Blk4	4.1259089	3.4198059	6.0006863	NA	NA	NA	
beta_tslope_GABA_FEZF2_right_X_Tns/Blk4	0.8016366	-1.4431424	2.5856529	0.0839756	14.1918727	0.0002959	ns
beta_tslope_GABA_FEZF2_18_X_Tns/Blk4	2.2174360	-1.2146629	3.1405350	0.0037033	0.9813832	0.0001887	ns
beta_tslope_GABA_FEZF2_right18_X_Tns/Blk4	-0.5727467	-1.5972328	1.8037126	0.3157842	35.6836153	0.0004425	ns
baseline_GABA_tslope_FEZF2_Tns/Blk5	4.8987296	3.4521340	5.7911654	NA	NA	NA	
beta_tslope_GABA_FEZF2_right_X_Tns/Blk5	2.1223920	0.2283298	3.5367483	0.0001001	0.0319287	0.0001567	*
beta_tslope_GABA_FEZF2_18_X_Tns/Blk5	1.4557452	-1.3367488	3.3920612	0.0037033	0.9850866	0.0001880	ns
beta_tslope_GABA_FEZF2_right18_X_Tns/Blk5	-0.6279028	-4.2704567	1.2453939	0.2524272	31.8058252	0.0003968	ns
baseline_GABA_tslope_NXPH3_Tns/Blk1	5.7208799	4.0799345	6.4721097	NA	NA	NA	
beta_tslope_GABA_NXPH3_right_X_Tns/Blk1	-0.0545553	-0.8833554	2.4691948	0.9694725	5.8168352	0.0083333	ns
beta_tslope_GABA_NXPH3_18_X_Tns/Blk1	0.2840404	-0.7828696	2.9160365	0.8032229	24.8999099	0.0016129	ns
beta_tslope_GABA_NXPH3_right18_X_Tns/Blk1	1.2034507	-2.2344419	3.1359139	0.2300070	30.3609248	0.0003788	ns
baseline_GABA_tslope_NXPH3_Tns/Blk2	5.4586913	2.9700896	6.4782902	NA	NA	NA	
beta_tslope_GABA_NXPH3_right_X_Tns/Blk2	3.0511744	-2.6043635	4.1903827	0.0051046	1.3118807	0.0001946	ns
beta_tslope_GABA_NXPH3_18_X_Tns/Blk2	3.3234412	-3.0755542	4.6918885	0.0028025	0.7650886	0.0001832	ns
beta_tslope_GABA_NXPH3_right18_X_Tns/Blk2	-1.8859903	-4.9888074	3.2457449	0.0721649	12.6288660	0.0002857	ns
baseline_GABA_tslope_RORB_Tns/Blk1	3.3980126	3.0428528	6.2688364	NA	NA	NA	
beta_tslope_GABA_RORB_right_X_Tns/Blk1	1.6081188	-1.7854483	4.1246004	0.0266240	5.5910319	0.0002381	ns
beta_tslope_GABA_RORB_18_X_Tns/Blk1	1.1430705	-2.0480605	3.7002771	0.0762686	13.0419377	0.0002924	ns
beta_tslope_GABA_RORB_right18_X_Tns/Blk1	0.7015853	-2.6562321	4.3703569	0.3958563	38.3980583	0.0005155	ns
baseline_GABA_tslope_RORB_Tns/Blk2	3.9277450	2.5789011	4.9401425	NA	NA	NA	
beta_tslope_GABA_RORB_right_X_Tns/Blk2	2.3900829	-1.2130620	5.9136216	0.0032029	0.8583725	0.0001866	ns
beta_tslope_GABA_RORB_18_X_Tns/Blk2	1.0887423	-2.5656282	3.0699474	0.0062056	1.5824242	0.0001961	ns
beta_tslope_GABA_RORB_right18_X_Tns/Blk2	-0.0876864	-3.1167275	2.4256522	0.8909018	15.1453308	0.0029412	ns
baseline_GABA_tslope_RORB_Tns/Blk3	4.2293420	2.9616197	6.0315026	NA	NA	NA	
beta_tslope_GABA_RORB_right_X_Tns/Blk3	2.3638948	-0.3882536	3.7601968	0.0001001	0.0318286	0.0001572	*
beta_tslope_GABA_RORB_18_X_Tns/Blk3	2.3970139	-0.2964903	4.2052661	0.0005005	0.1536383	0.0001629	ns
beta_tslope_GABA_RORB_right18_X_Tns/Blk3	0.3394400	-1.8594896	2.8611151	0.6972275	35.5586027	0.0009804	ns
baseline_GABA_tslope_RORB_Tns/Blk4	3.9879660	3.1603809	5.9459322	NA	NA	NA	
beta_tslope_GABA_RORB_right_X_Tns/Blk4	2.3526750	-0.2425766	3.7579690	0.0013012	0.3786408	0.0001718	ns

Continued on next page

parameter	estimate	CI.low	CI.high	p.value	p.value.adj	alpha.adj	sig
beta_tslope_GABA_RORB_18_X_Tns/Blk4	2.0059081	-0.3234195	3.6502702	0.0009008	0.2666400	0.0001689	ns
beta_tslope_GABA_RORB_right18_X_Tns/Blk4	-1.0045401	-2.0543291	2.4220728	0.1917726	26.8481633	0.0003571	ns
baseline_GABA_tslope_RORB_Tns/Blk5	5.9273598	4.5645174	6.6937901	NA	NA	NA	
beta_tslope_GABA_RORB_right_X_Tns/Blk5	0.5138537	-0.4330517	2.4998742	0.1076969	17.4469022	0.0003086	ns
beta_tslope_GABA_RORB_18_X_Tns/Blk5	1.7604046	-0.5627260	3.4888659	0.0111100	2.6108498	0.0002128	ns
beta_tslope_GABA_RORB_right18_X_Tns/Blk5	0.2229627	-1.1894889	2.7825816	0.7353618	30.8851967	0.0011905	ns
baseline_GABA_tslope_SATB2_Tns/Blk1	2.5611451	1.2442595	4.7452014	NA	NA	NA	
beta_tslope_GABA_SATB2_right_X_Tns/Blk1	1.5463853	-2.1019225	3.5473853	0.0238214	5.0501451	0.0002358	ns
beta_tslope_GABA_SATB2_18_X_Tns/Blk1	2.5539506	-0.9086039	4.7474106	0.0024022	0.6702032	0.0001792	ns
beta_tslope_GABA_SATB2_right18_X_Tns/Blk1	1.4814305	-1.6785320	2.9799119	0.0463417	8.8512661	0.0002618	ns
baseline_GABA_tslope_SATB2_Tns/Blk2	6.2860786	3.6507339	6.7822119	NA	NA	NA	
beta_tslope_GABA_SATB2_right_X_Tns/Blk2	1.8796573	-3.8382761	3.8893594	0.0621559	11.3123811	0.0002747	ns
beta_tslope_GABA_SATB2_18_X_Tns/Blk2	1.8386848	-3.3561196	4.0763206	0.0336303	6.8605745	0.0002451	ns
beta_tslope_GABA_SATB2_right18_X_Tns/Blk2	0.4013356	-1.8154056	4.6815061	0.4713242	39.1199079	0.0006024	ns
baseline_GABA_tslope_SATB2_Tns/Blk3	5.7400005	4.1986517	6.4827336	NA	NA	NA	
beta_tslope_GABA_SATB2_right_X_Tns/Blk3	-1.5697749	-2.9170382	1.1128302	0.0109098	2.5747172	0.0002119	ns
beta_tslope_GABA_SATB2_18_X_Tns/Blk3	0.0042421	-0.7531515	1.5494084	0.9947953	0.9947953	0.0500000	ns
beta_tslope_GABA_SATB2_right18_X_Tns/Blk3	-1.8084125	-3.4780161	1.0682209	0.0164148	3.6276649	0.0002262	ns
baseline_GABA_tslope_SATB2_Tns/Blk4	6.1264096	3.9574301	6.9656409	NA	NA	NA	
beta_tslope_GABA_SATB2_right_X_Tns/Blk4	0.7919911	-1.4325450	2.8846211	0.1032930	16.9400460	0.0003049	ns
beta_tslope_GABA_SATB2_18_X_Tns/Blk4	1.1011526	-2.1549939	3.3551707	0.1151036	18.4165749	0.0003125	ns
beta_tslope_GABA_SATB2_right18_X_Tns/Blk4	-0.7686281	-3.7139910	2.3154608	0.1894705	26.7153438	0.0003546	ns
baseline_GABA_tslope_SATB2_Tns/Blk5	5.6501525	4.3285355	6.5900227	NA	NA	NA	
beta_tslope_GABA_SATB2_right_X_Tns/Blk5	0.2460982	-3.6795563	2.9008297	0.7045341	34.5221700	0.0010204	ns
beta_tslope_GABA_SATB2_18_X_Tns/Blk5	0.5823229	-2.5570045	3.1759154	0.2947653	34.7823041	0.0004237	ns
beta_tslope_GABA_SATB2_right18_X_Tns/Blk5	2.3288468	-2.3342603	3.3695084	0.0066059	1.6646982	0.0001984	ns
baseline_Glut_tslope_BCL11B_Tns/Blk1	4.3175307	2.4486944	5.6457737	NA	NA	NA	
beta_tslope_Glut_BCL11B_right_X_Tns/Blk1	0.7724696	-4.5173900	2.9524313	0.6931238	37.4286858	0.0009259	ns
beta_tslope_Glut_BCL11B_18_X_Tns/Blk1	-3.8753860	-5.5182335	2.8826053	0.0028025	0.7622861	0.0001838	ns
beta_tslope_Glut_BCL11B_right18_X_Tns/Blk1	-0.8770393	-4.5270276	5.4202261	0.6089481	38.3637274	0.0007937	ns
baseline_Glut_tslope_BCL11B_Tns/Blk2	1.0599015	0.4900954	4.4186241	NA	NA	NA	
beta_tslope_Glut_BCL11B_right_X_Tns/Blk2	1.5229027	-4.2267301	4.6744434	0.2490241	31.6260635	0.0003937	ns
beta_tslope_Glut_BCL11B_18_X_Tns/Blk2	-0.1025375	-3.3141499	2.6664925	0.9243319	12.0163147	0.0038462	ns
beta_tslope_Glut_BCL11B_right18_X_Tns/Blk2	-2.1899429	-5.9745013	2.6426032	0.0638575	11.5582024	0.0002762	ns
baseline_Glut_tslope_BCL11B_Tns/Blk3	3.8365813	2.4590020	6.2192428	NA	NA	NA	
beta_tslope_Glut_BCL11B_right_X_Tns/Blk3	3.1933862	-1.6590679	4.1208380	0.0007006	0.2108898	0.0001661	ns
beta_tslope_Glut_BCL11B_18_X_Tns/Blk3	1.3281477	-1.3285628	4.1095781	0.1828646	26.6982284	0.0003425	ns
beta_tslope_Glut_BCL11B_right18_X_Tns/Blk3	0.8619993	-2.1320361	3.3809904	0.4138725	38.9040136	0.0005319	ns
baseline_Glut_tslope_CUX2_Tns/Blk1	0.4396114	0.3420971	0.6756717	NA	NA	NA	
beta_tslope_Glut_CUX2_right_X_Tns/Blk1	0.9934570	-0.5751006	4.5380391	0.2145931	28.7554799	0.0003731	ns
beta_tslope_Glut_CUX2_18_X_Tns/Blk1	0.1556226	-0.6609902	0.9544342	0.1849665	26.8201381	0.0003448	ns
beta_tslope_Glut_CUX2_right18_X_Tns/Blk1	-1.0525081	-4.6328278	2.0066574	0.2006806	27.4932439	0.0003650	ns
baseline_Glut_tslope_CUX2_Tns/Blk2	3.2781859	2.7159895	5.3865518	NA	NA	NA	
beta_tslope_Glut_CUX2_right_X_Tns/Blk2	0.7687009	-0.4757711	3.2614892	0.1985787	27.6024422	0.0003597	ns
beta_tslope_Glut_CUX2_18_X_Tns/Blk2	0.5956249	-0.7205624	1.9484801	0.2372135	30.6005405	0.0003876	ns
beta_tslope_Glut_CUX2_right18_X_Tns/Blk2	0.4080989	-0.9902590	2.9194965	0.6163547	38.2139926	0.0008065	ns
baseline_Glut_tslope_CUX2_Tns/Blk3	5.1211117	3.3172763	6.6541938	NA	NA	NA	
beta_tslope_Glut_CUX2_right_X_Tns/Blk3	2.4819717	-1.3917103	3.6797256	0.0055050	1.4092683	0.0001953	ns
beta_tslope_Glut_CUX2_18_X_Tns/Blk3	1.4635061	-0.3912665	3.7030366	0.0409368	7.9826844	0.0002564	ns
beta_tslope_Glut_CUX2_right18_X_Tns/Blk3	0.9647903	-1.9455838	2.9271337	0.3116805	35.5315784	0.0004386	ns
baseline_Glut_tslope_FEZF2_Tns/Blk1	3.4343468	1.3620295	4.7683931	NA	NA	NA	
beta_tslope_Glut_FEZF2_right_X_Tns/Blk1	-1.1355968	-4.4474337	3.0842208	0.4897408	39.6690021	0.0006173	ns
beta_tslope_Glut_FEZF2_18_X_Tns/Blk1	-3.2639994	-5.0862751	2.2596175	0.0394355	7.7293564	0.0002551	ns
beta_tslope_Glut_FEZF2_right18_X_Tns/Blk1	1.0804718	-5.0367877	4.6577313	0.5823241	39.0157141	0.0007463	ns
baseline_Glut_tslope_FEZF2_Tns/Blk2	0.4511706	0.3309937	1.5218220	NA	NA	NA	
beta_tslope_Glut_FEZF2_right_X_Tns/Blk2	2.0062596	0.0254890	6.4530043	0.0079071	1.9688720	0.0002008	ns
beta_tslope_Glut_FEZF2_18_X_Tns/Blk2	0.2219403	-2.7125064	3.3202138	0.3925533	38.4702232	0.0005102	ns
beta_tslope_Glut_FEZF2_right18_X_Tns/Blk2	-1.8832669	-5.0063356	2.8670604	0.0279251	5.8084276	0.0002404	ns
baseline_Glut_tslope_FEZF2_Tns/Blk3	1.8275546	1.0235020	5.4349465	NA	NA	NA	

Continued on next page

parameter	estimate	CI.low	CI.high	p.value	p.value.adj	alpha.adj	sig
beta_tslope_Glut_FEZF2_right_X_Tns/Blk3	-0.7086463	-3.4604157	3.8128745	0.5248724	38.8405565	0.0006757	ns
beta_tslope_Glut_FEZF2_18_X_Tns/Blk3	-0.2408617	-3.3594280	3.6839222	0.8399560	18.4790311	0.0022727	ns
beta_tslope_Glut_FEZF2_right18_X_Tns/Blk3	0.3934699	-2.7627967	4.5067947	0.7324592	31.4957462	0.0011628	ns
baseline_Glut_tslope_NXPH3_Tns/Blk1	0.4496032	0.3467242	4.8240237	NA	NA	NA	
beta_tslope_Glut_NXPH3_right_X_Tns/Blk1	0.1237115	-2.8636760	3.3426779	0.8558703	17.1174057	0.0025000	ns
beta_tslope_Glut_NXPH3_18_X_Tns/Blk1	2.2668953	-0.6947573	4.3866343	0.0027024	0.7404664	0.0001825	ns
beta_tslope_Glut_NXPH3_right18_X_Tns/Blk1	1.3697017	-1.2051974	2.9560337	0.0269242	5.6271644	0.0002392	ns
baseline_Glut_tslope_NXPH3_Tns/Blk2	2.5501294	0.7266250	4.4475336	NA	NA	NA	
beta_tslope_Glut_NXPH3_right_X_Tns/Blk2	-1.4627209	-3.9527463	3.2547450	0.1566410	23.6527875	0.0003311	ns
beta_tslope_Glut_NXPH3_18_X_Tns/Blk2	0.4825128	-2.9823393	4.1024211	0.5921329	37.8965069	0.0007813	ns
beta_tslope_Glut_NXPH3_right18_X_Tns/Blk2	-0.7638621	-3.6515032	3.4600036	0.4563107	39.6990291	0.0005747	ns
baseline_Glut_tslope_NXPH3_Tns/Blk3	3.8322517	0.9943575	7.2220923	NA	NA	NA	
beta_tslope_Glut_NXPH3_right_X_Tns/Blk3	2.9812048	-0.1983295	6.0561455	0.0006005	0.1825643	0.0001645	ns
beta_tslope_Glut_NXPH3_18_X_Tns/Blk3	2.8574379	-0.0154394	6.0241664	0.0006005	0.1819638	0.0001650	ns
beta_tslope_Glut_NXPH3_right18_X_Tns/Blk3	-0.5946026	-1.8558047	2.3425538	0.3371034	37.7555800	0.0004464	ns
baseline_Glut_tslope_NXPH3_Tns/Blk4	4.3839794	2.0623737	4.7628972	NA	NA	NA	
beta_tslope_Glut_NXPH3_right_X_Tns/Blk4	0.5537195	-2.3003239	4.9015180	0.4904414	39.2353118	0.0006250	ns
beta_tslope_Glut_NXPH3_18_X_Tns/Blk4	-4.1807408	-5.5304806	0.0719335	0.0000000	0.0000000	0.0001319	***
beta_tslope_Glut_NXPH3_right18_X_Tns/Blk4	-0.5756142	-2.7148018	4.3922719	0.5706136	39.9429487	0.0007143	ns
baseline_Glut_tslope_RORB_Tns/Blk1	1.3475468	0.4061117	3.4133476	NA	NA	NA	
beta_tslope_Glut_RORB_right_X_Tns/Blk1	-0.9409314	-4.0548293	5.3728436	0.3600240	37.4424982	0.0004808	ns
beta_tslope_Glut_RORB_18_X_Tns/Blk1	-1.1012695	-4.5250987	4.6871519	0.1322190	20.8906015	0.0003165	ns
beta_tslope_Glut_RORB_right18_X_Tns/Blk1	1.1833716	-4.7980369	6.6903598	0.3447102	37.9181263	0.0004545	ns
baseline_Glut_tslope_RORB_Tns/Blk2	0.5186540	0.3611848	1.1081735	NA	NA	NA	
beta_tslope_Glut_RORB_right_X_Tns/Blk2	0.0249696	-1.6460350	2.9903325	0.9059153	13.5887299	0.0033333	ns
beta_tslope_Glut_RORB_18_X_Tns/Blk2	-0.2407776	-2.9889925	0.1669404	0.0968872	16.0832749	0.0003012	ns
beta_tslope_Glut_RORB_right18_X_Tns/Blk2	-0.0709034	-3.3056889	2.0399873	0.7502752	28.5104594	0.0013158	ns
baseline_Glut_tslope_RORB_Tns/Blk3	0.4852875	0.3169402	1.3153101	NA	NA	NA	
beta_tslope_Glut_RORB_right_X_Tns/Blk3	-0.1241287	-2.0835852	2.7671321	0.5740166	39.6071464	0.0007246	ns
beta_tslope_Glut_RORB_18_X_Tns/Blk3	-0.0950582	-2.0092809	0.1907438	0.7301571	32.1269142	0.0011364	ns
beta_tslope_Glut_RORB_right18_X_Tns/Blk3	0.0114759	-2.3741702	1.4478804	0.9586628	9.5866280	0.0050000	ns
baseline_Glut_tslope_SATB2_Tns/Blk1	3.4860475	2.5863298	5.6046334	NA	NA	NA	
beta_tslope_Glut_SATB2_right_X_Tns/Blk1	1.6711822	-0.6575656	3.5851486	0.0185167	4.0181163	0.0002304	ns
beta_tslope_Glut_SATB2_18_X_Tns/Blk1	0.4267372	-2.0135943	2.8491914	0.5858272	38.6645981	0.0007576	ns
beta_tslope_Glut_SATB2_right18_X_Tns/Blk1	1.0320616	-2.0782109	3.0459005	0.2927635	34.8388550	0.0004202	ns
baseline_Glut_tslope_SATB2_Tns/Blk2	4.3575237	3.1534192	5.4735502	NA	NA	NA	
beta_tslope_Glut_SATB2_right_X_Tns/Blk2	2.5162660	0.0280218	3.4247966	0.0000000	0.0000000	0.0001323	***
beta_tslope_Glut_SATB2_18_X_Tns/Blk2	1.7096470	0.3519595	2.9048305	0.0000000	0.0000000	0.0001326	***
beta_tslope_Glut_SATB2_right18_X_Tns/Blk2	-0.0664251	-0.6884387	2.2581022	0.9302372	11.1628466	0.0041667	ns
baseline_GABA_tpoint_BCL11B_Tns/Blk1	12.6455649	11.4769778	14.5333998	NA	NA	NA	
beta_tpoint_GABA_BCL11B_right_X_Tns/Blk1	-1.9279348	-4.4760707	-0.2371486	0.0016014	0.4596137	0.0001742	ns
beta_tpoint_GABA_BCL11B_18_X_Tns/Blk1	0.5590151	-1.8804685	3.0543967	0.3570213	37.8442598	0.0004717	ns
beta_tpoint_GABA_BCL11B_right18_X_Tns/Blk1	3.6730210	0.9274932	6.3839079	0.0000000	0.0000000	0.0001330	***
baseline_GABA_tpoint_CUX2_Tns/Blk1	29.2873810	28.8683617	30.5322264	NA	NA	NA	
beta_tpoint_GABA_CUX2_right_X_Tns/Blk1	-0.7423644	-2.3751850	1.6759934	0.0675608	12.0933840	0.0002793	ns
beta_tpoint_GABA_CUX2_18_X_Tns/Blk1	5.1219631	3.2771022	7.5429069	0.0000000	0.0000000	0.0001333	***
beta_tpoint_GABA_CUX2_right18_X_Tns/Blk1	-4.3989669	-6.0983621	-2.5062705	0.0000000	0.0000000	0.0001337	***
baseline_GABA_tpoint_CUX2_Tns/Blk2	36.7002191	36.1761802	38.1946088	NA	NA	NA	
beta_tpoint_GABA_CUX2_right_X_Tns/Blk2	-0.3163762	-2.2704857	2.3250604	0.5176659	39.3426083	0.0006579	ns
beta_tpoint_GABA_CUX2_18_X_Tns/Blk2	4.4377822	2.6760690	7.7854304	0.0000000	0.0000000	0.0001340	***
beta_tpoint_GABA_CUX2_right18_X_Tns/Blk2	-3.5817453	-5.9289649	-1.1066130	0.0000000	0.0000000	0.0001344	***
baseline_GABA_tpoint_CUX2_Tns/Blk3	57.6676912	56.2270837	58.6132051	NA	NA	NA	
beta_tpoint_GABA_CUX2_right_X_Tns/Blk3	-3.0337549	-5.1781307	-0.4879069	0.0000000	0.0000000	0.0001348	***
beta_tpoint_GABA_CUX2_18_X_Tns/Blk3	6.5465888	3.8551747	9.6126205	0.0000000	0.0000000	0.0001351	***
beta_tpoint_GABA_CUX2_right18_X_Tns/Blk3	0.4661015	-1.3848413	3.6221819	0.3095786	35.9111200	0.0004310	ns
baseline_GABA_tpoint_CUX2_Tns/Blk4	70.3493897	69.3957726	71.6601283	NA	NA	NA	
beta_tpoint_GABA_CUX2_right_X_Tns/Blk4	-3.8243286	-5.5937492	-1.5412365	0.0000000	0.0000000	0.0001355	***
beta_tpoint_GABA_CUX2_18_X_Tns/Blk4	6.0156772	3.1837992	7.9665421	0.0000000	0.0000000	0.0001359	***
beta_tpoint_GABA_CUX2_right18_X_Tns/Blk4	0.5450030	-1.4368632	2.6077252	0.2427184	31.0679612	0.0003906	ns

Continued on next page

parameter	estimate	CI.low	CI.high	p.value	p.value.adj	alpha.adj	sig
baseline_GABA_tpoint_CUX2_Tns/Blk5	77.0201213	76.8566789	78.9984839	NA	NA	NA	
beta_tpoint_GABA_CUX2_right_X_Tns/Blk5	-3.3793762	-5.5784584	0.0155026	0.0002002	0.0628566	0.0001592	ns
beta_tpoint_GABA_CUX2_18_X_Tns/Blk5	5.9561421	3.0087753	8.5830720	0.0000000	0.0000000	0.0001362	***
beta_tpoint_GABA_CUX2_right18_X_Tns/Blk5	0.1624106	-2.5066872	2.1188090	0.7851066	25.9085177	0.0015152	ns
baseline_GABA_tpoint_FEZF2_Tns/Blk1	23.4262021	23.0706659	24.1257411	NA	NA	NA	
beta_tpoint_GABA_FEZF2_right_X_Tns/Blk1	-12.3453981	-13.4189663	-11.3004514	0.0000000	0.0000000	0.0001366	***
beta_tpoint_GABA_FEZF2_18_X_Tns/Blk1	-12.1127966	-12.7051740	-10.9070564	0.0000000	0.0000000	0.0001370	***
beta_tpoint_GABA_FEZF2_right18_X_Tns/Blk1	13.4234753	12.6710348	14.3005804	0.0000000	0.0000000	0.0001374	***
baseline_GABA_tpoint_FEZF2_Tns/Blk2	39.6519141	38.8777573	41.0258547	NA	NA	NA	
beta_tpoint_GABA_FEZF2_right_X_Tns/Blk2	-5.9355180	-7.1409802	-3.6290883	0.0000000	0.0000000	0.0001377	***
beta_tpoint_GABA_FEZF2_18_X_Tns/Blk2	-2.6830719	-5.3127632	-0.7393235	0.0000000	0.0000000	0.0001381	***
beta_tpoint_GABA_FEZF2_right18_X_Tns/Blk2	1.7156582	0.8075103	3.8556860	0.0014013	0.4063657	0.0001724	ns
baseline_GABA_tpoint_FEZF2_Tns/Blk3	51.2600525	51.0832484	53.1014575	NA	NA	NA	
beta_tpoint_GABA_FEZF2_right_X_Tns/Blk3	-3.6317074	-6.0109048	-1.7301443	0.0000000	0.0000000	0.0001385	***
beta_tpoint_GABA_FEZF2_18_X_Tns/Blk3	2.1412278	-0.4539432	4.4568630	0.0006005	0.1831648	0.0001639	ns
beta_tpoint_GABA_FEZF2_right18_X_Tns/Blk3	3.6307369	1.1247875	5.1866548	0.0000000	0.0000000	0.0001389	***
baseline_GABA_tpoint_FEZF2_Tns/Blk4	64.5674432	64.2729100	66.0971948	NA	NA	NA	
beta_tpoint_GABA_FEZF2_right_X_Tns/Blk4	-5.7716864	-8.8233306	-4.5567587	0.0000000	0.0000000	0.0001393	***
beta_tpoint_GABA_FEZF2_18_X_Tns/Blk4	2.2442110	1.1284173	5.3626721	0.0002002	0.0634571	0.0001577	ns
beta_tpoint_GABA_FEZF2_right18_X_Tns/Blk4	4.3097826	1.7023616	5.8822533	0.0000000	0.0000000	0.0001397	***
baseline_GABA_tpoint_FEZF2_Tns/Blk5	72.2578399	71.7668728	73.5665194	NA	NA	NA	
beta_tpoint_GABA_FEZF2_right_X_Tns/Blk5	2.4797428	0.6551937	3.5432622	0.0000000	0.0000000	0.0001401	***
beta_tpoint_GABA_FEZF2_18_X_Tns/Blk5	3.5148971	1.8855804	5.5859336	0.0000000	0.0000000	0.0001404	***
beta_tpoint_GABA_FEZF2_right18_X_Tns/Blk5	-3.9114436	-5.3705793	-2.7741569	0.0000000	0.0000000	0.0001408	***
baseline_GABA_tpoint_NXPH3_Tns/Blk1	13.9025604	14.0514599	16.3409272	NA	NA	NA	
beta_tpoint_GABA_NXPH3_right_X_Tns/Blk1	-5.6528938	-8.7711922	-3.4126738	0.0000000	0.0000000	0.0001412	***
beta_tpoint_GABA_NXPH3_18_X_Tns/Blk1	-5.8004223	-8.8397086	-2.7797553	0.0000000	0.0000000	0.0001416	***
beta_tpoint_GABA_NXPH3_right18_X_Tns/Blk1	8.0979032	4.9092954	9.7491377	0.0000000	0.0000000	0.0001420	***
baseline_GABA_tpoint_NXPH3_Tns/Blk2	58.3374256	57.7219307	60.9123414	NA	NA	NA	
beta_tpoint_GABA_NXPH3_right_X_Tns/Blk2	-3.1861231	-6.9143740	0.3193266	0.0013012	0.3799419	0.0001712	ns
beta_tpoint_GABA_NXPH3_18_X_Tns/Blk2	7.2238051	1.7500411	9.9855463	0.0000000	0.0000000	0.0001425	***
beta_tpoint_GABA_NXPH3_right18_X_Tns/Blk2	-2.5787809	-6.2489961	2.1004777	0.0162146	3.5996397	0.0002252	ns
baseline_GABA_tpoint_RORB_Tns/Blk1	6.7505383	6.5571445	7.5913288	NA	NA	NA	
beta_tpoint_GABA_RORB_right_X_Tns/Blk1	12.1571141	11.1588668	14.2402027	0.0000000	0.0000000	0.0001429	***
beta_tpoint_GABA_RORB_18_X_Tns/Blk1	2.6615717	0.5652025	4.3091548	0.0000000	0.0000000	0.0001433	***
beta_tpoint_GABA_RORB_right18_X_Tns/Blk1	-8.8185053	-10.1242515	-6.8453919	0.0000000	0.0000000	0.0001437	***
baseline_GABA_tpoint_RORB_Tns/Blk2	15.1528361	14.5808832	15.9168899	NA	NA	NA	
beta_tpoint_GABA_RORB_right_X_Tns/Blk2	11.7374245	10.5236103	13.8958876	0.0000000	0.0000000	0.0001441	***
beta_tpoint_GABA_RORB_18_X_Tns/Blk2	1.4134245	-0.7412719	3.6395179	0.0094085	2.2768492	0.0002066	ns
beta_tpoint_GABA_RORB_right18_X_Tns/Blk2	-8.2155880	-9.0701935	-6.5111325	0.0000000	0.0000000	0.0001445	***
baseline_GABA_tpoint_RORB_Tns/Blk3	26.3803552	25.7259590	27.5469353	NA	NA	NA	
beta_tpoint_GABA_RORB_right_X_Tns/Blk3	8.7572953	5.3035579	10.5335850	0.0000000	0.0000000	0.0001449	***
beta_tpoint_GABA_RORB_18_X_Tns/Blk3	9.7847275	7.9374620	12.5731701	0.0000000	0.0000000	0.0001453	***
beta_tpoint_GABA_RORB_right18_X_Tns/Blk3	-10.7553651	-12.7475992	-8.4167073	0.0000000	0.0000000	0.0001458	***
baseline_GABA_tpoint_RORB_Tns/Blk4	55.5628184	53.9088767	55.8005297	NA	NA	NA	
beta_tpoint_GABA_RORB_right_X_Tns/Blk4	-2.7591224	-4.1542604	-0.1845714	0.0000000	0.0000000	0.0001462	***
beta_tpoint_GABA_RORB_18_X_Tns/Blk4	-0.6575416	-3.3333080	0.6108784	0.1351216	21.2140927	0.0003185	ns
beta_tpoint_GABA_RORB_right18_X_Tns/Blk4	10.0817037	6.6680104	11.6664198	0.0000000	0.0000000	0.0001466	***
baseline_GABA_tpoint_RORB_Tns/Blk5	83.0560709	82.2084976	84.2426082	NA	NA	NA	
beta_tpoint_GABA_RORB_right_X_Tns/Blk5	3.3338233	1.2846752	5.6785660	0.0000000	0.0000000	0.0001471	***
beta_tpoint_GABA_RORB_18_X_Tns/Blk5	-0.4433076	-2.8429686	1.2728175	0.3597238	37.7709939	0.0004762	ns
beta_tpoint_GABA_RORB_right18_X_Tns/Blk5	-0.7317516	-3.2515058	0.2371916	0.0850766	14.2928636	0.0002976	ns
baseline_GABA_tpoint_SATB2_Tns/Blk1	15.1304019	15.1113088	18.0199346	NA	NA	NA	
beta_tpoint_GABA_SATB2_right_X_Tns/Blk1	-3.4379715	-6.8425302	-1.7778242	0.0000000	0.0000000	0.0001475	***
beta_tpoint_GABA_SATB2_18_X_Tns/Blk1	-5.9066005	-8.0974403	-2.6606221	0.0000000	0.0000000	0.0001479	***
beta_tpoint_GABA_SATB2_right18_X_Tns/Blk1	2.0356545	-1.0370601	3.9462294	0.0021019	0.5969372	0.0001761	ns
baseline_GABA_tpoint_SATB2_Tns/Blk2	23.1320816	23.0631535	25.3315451	NA	NA	NA	
beta_tpoint_GABA_SATB2_right_X_Tns/Blk2	-0.9443888	-4.6671447	1.3645148	0.1352217	21.0945851	0.0003205	ns
beta_tpoint_GABA_SATB2_18_X_Tns/Blk2	-5.2000535	-7.0759839	-0.9853324	0.0000000	0.0000000	0.0001484	***

Continued on next page

parameter	estimate	CI.low	CI.high	p.value	p.value.adj	alpha.adj	sig
beta_tpoint_GABA_SATB2_right18_X_Tns/Blk2	-1.5431153	-4.9842015	0.0715308	0.0117105	2.7402662	0.0002137	ns
baseline_GABA_tpoint_SATB2_Tns/Blk3	36.1904031	35.0246236	37.2627174	NA	NA	NA	
beta_tpoint_GABA_SATB2_right_X_Tns/Blk3	-0.7358645	-4.7327970	0.7800123	0.4452007	39.6228606	0.0005618	ns
beta_tpoint_GABA_SATB2_18_X_Tns/Blk3	0.3983927	-1.3558601	3.5284065	0.5339806	38.9805825	0.0006849	ns
beta_tpoint_GABA_SATB2_right18_X_Tns/Blk3	-3.4592308	-8.3504919	-1.2309840	0.0008007	0.2402162	0.0001667	ns
baseline_GABA_tpoint_SATB2_Tns/Blk4	60.0300847	58.1659387	60.8417932	NA	NA	NA	
beta_tpoint_GABA_SATB2_right_X_Tns/Blk4	1.1730714	-2.6744935	3.8786431	0.0663597	11.9447503	0.0002778	ns
beta_tpoint_GABA_SATB2_18_X_Tns/Blk4	1.4133861	-0.4405619	6.3064120	0.1540386	23.4138725	0.0003289	ns
beta_tpoint_GABA_SATB2_right18_X_Tns/Blk4	-8.1866158	-9.8025574	-2.0019980	0.0000000	0.0000000	0.0001488	***
baseline_GABA_tpoint_SATB2_Tns/Blk5	74.6601397	73.9895246	76.6491187	NA	NA	NA	
beta_tpoint_GABA_SATB2_right_X_Tns/Blk5	7.2037077	1.9003646	8.5323878	0.0000000	0.0000000	0.0001493	***
beta_tpoint_GABA_SATB2_18_X_Tns/Blk5	-2.4547550	-4.8448801	2.1485657	0.0063057	1.6016415	0.0001969	ns
beta_tpoint_GABA_SATB2_right18_X_Tns/Blk5	-5.9383672	-8.2355427	-2.7123994	0.0000000	0.0000000	0.0001497	***
baseline_Glut_tpoint_BCL11B_Tns/Blk1	6.9635248	6.7390812	9.7897678	NA	NA	NA	
beta_tpoint_Glut_BCL11B_right_X_Tns/Blk1	0.7381642	-2.3493072	5.6202379	0.3411070	37.8628766	0.0004505	ns
beta_tpoint_Glut_BCL11B_18_X_Tns/Blk1	3.0264626	-1.6522119	5.6805773	0.0037033	0.9776799	0.0001894	ns
beta_tpoint_Glut_BCL11B_right18_X_Tns/Blk1	3.3659111	-1.1030283	9.1153984	0.0072065	1.8016215	0.0002000	ns
baseline_Glut_tpoint_BCL11B_Tns/Blk2	57.6400431	55.9275831	59.1444824	NA	NA	NA	
beta_tpoint_Glut_BCL11B_right_X_Tns/Blk2	4.2166327	0.3114508	7.3299458	0.0001001	0.0320288	0.0001562	*
beta_tpoint_Glut_BCL11B_18_X_Tns/Blk2	4.6918621	1.9700720	8.5781787	0.0000000	0.0000000	0.0001502	***
beta_tpoint_Glut_BCL11B_right18_X_Tns/Blk2	-4.1543445	-8.0273978	1.6861689	0.0021019	0.5927335	0.0001773	ns
baseline_Glut_tpoint_BCL11B_Tns/Blk3	79.6395545	78.2419491	82.0046562	NA	NA	NA	
beta_tpoint_Glut_BCL11B_right_X_Tns/Blk3	0.5146694	-1.9759732	4.0704952	0.4221800	39.2627365	0.0005376	ns
beta_tpoint_Glut_BCL11B_18_X_Tns/Blk3	1.3512775	-1.3673765	5.9169255	0.0725653	12.6263637	0.0002874	ns
beta_tpoint_Glut_BCL11B_right18_X_Tns/Blk3	-3.1485466	-5.8021405	-0.1118114	0.0000000	0.0000000	0.0001506	***
baseline_Glut_tpoint_CUX2_Tns/Blk1	65.6974743	65.3472278	67.2742468	NA	NA	NA	
beta_tpoint_Glut_CUX2_right_X_Tns/Blk1	-0.3954009	-2.7043509	1.4481072	0.5038535	39.3005705	0.0006410	ns
beta_tpoint_Glut_CUX2_18_X_Tns/Blk1	-0.2683915	-2.1207961	1.6958712	0.5744170	39.0603543	0.0007353	ns
beta_tpoint_Glut_CUX2_right18_X_Tns/Blk1	1.4010387	-0.7228847	3.8245649	0.0220198	4.7122410	0.0002336	ns
baseline_Glut_tpoint_CUX2_Tns/Blk2	73.3722210	73.2414687	74.6291251	NA	NA	NA	
beta_tpoint_Glut_CUX2_right_X_Tns/Blk2	-1.2866491	-2.6779876	0.6519860	0.0049044	1.2702432	0.0001931	ns
beta_tpoint_Glut_CUX2_18_X_Tns/Blk2	-0.1970248	-1.7351512	1.1225571	0.5891302	38.2934641	0.0007692	ns
beta_tpoint_Glut_CUX2_right18_X_Tns/Blk2	2.1355286	0.4685455	3.9732669	0.0002002	0.0626564	0.0001597	ns
baseline_Glut_tpoint_CUX2_Tns/Blk3	92.9741499	92.5126971	93.3512224	NA	NA	NA	
beta_tpoint_Glut_CUX2_right_X_Tns/Blk3	-1.9390953	-3.3594280	-0.9941492	0.0000000	0.0000000	0.0001511	***
beta_tpoint_Glut_CUX2_18_X_Tns/Blk3	-0.3662717	-0.9691985	0.4014085	0.0553498	10.3504154	0.0002674	ns
beta_tpoint_Glut_CUX2_right18_X_Tns/Blk3	2.4928576	1.4559387	3.3940014	0.0000000	0.0000000	0.0001515	***
baseline_Glut_tpoint_FEZF2_Tns/Blk1	37.1693941	35.1142899	42.0966426	NA	NA	NA	
beta_tpoint_Glut_FEZF2_right_X_Tns/Blk1	-10.9584469	-18.3537712	-2.4281804	0.0000000	0.0000000	0.0001520	***
beta_tpoint_Glut_FEZF2_18_X_Tns/Blk1	1.3139806	-4.7662935	7.5488132	0.3846462	38.8492643	0.0004950	ns
beta_tpoint_Glut_FEZF2_right18_X_Tns/Blk1	4.6153199	-4.9589075	10.6326969	0.0086077	2.1088980	0.0002041	ns
baseline_Glut_tpoint_FEZF2_Tns/Blk2	66.0053833	63.8613492	67.0573361	NA	NA	NA	
beta_tpoint_Glut_FEZF2_right_X_Tns/Blk2	-3.7981504	-6.8955681	0.6031668	0.0003003	0.0930838	0.0001613	ns
beta_tpoint_Glut_FEZF2_18_X_Tns/Blk2	1.0439532	-1.0518283	4.9408020	0.1693524	25.4028626	0.0003333	ns
beta_tpoint_Glut_FEZF2_right18_X_Tns/Blk2	2.2486001	-1.4667378	5.6876854	0.0159143	3.5648083	0.0002232	ns
baseline_Glut_tpoint_FEZF2_Tns/Blk3	73.1138734	71.5389438	74.5420047	NA	NA	NA	
beta_tpoint_Glut_FEZF2_right_X_Tns/Blk3	-3.3211822	-5.5248839	0.9317583	0.0003003	0.0927835	0.0001618	ns
beta_tpoint_Glut_FEZF2_18_X_Tns/Blk3	1.4333501	-0.7551920	5.7189759	0.0752677	12.9460514	0.0002907	ns
beta_tpoint_Glut_FEZF2_right18_X_Tns/Blk3	1.7094062	-2.3701963	4.2560666	0.0465419	8.8429587	0.0002632	ns
baseline_Glut_tpoint_NXPH3_Tns/Blk1	24.8670612	22.1669107	26.2080421	NA	NA	NA	
beta_tpoint_Glut_NXPH3_right_X_Tns/Blk1	2.8957706	-2.3605859	5.9183677	0.0099089	2.3880492	0.0002075	ns
beta_tpoint_Glut_NXPH3_18_X_Tns/Blk1	-0.6676021	-6.1316126	1.1854925	0.4564108	39.2513262	0.0005814	ns
beta_tpoint_Glut_NXPH3_right18_X_Tns/Blk1	1.7887496	-4.2422525	4.0992847	0.0681613	12.1327194	0.0002809	ns
baseline_Glut_tpoint_NXPH3_Tns/Blk2	36.3726353	34.4560825	37.3116401	NA	NA	NA	
beta_tpoint_Glut_NXPH3_right_X_Tns/Blk2	4.1335901	-2.5734520	6.4688550	0.0046041	1.2016815	0.0001916	ns
beta_tpoint_Glut_NXPH3_18_X_Tns/Blk2	-3.3404533	-6.8302458	0.1370944	0.0004004	0.1233110	0.0001623	ns
beta_tpoint_Glut_NXPH3_right18_X_Tns/Blk2	5.5962645	0.3321877	9.5149812	0.0000000	0.0000000	0.0001524	***
baseline_Glut_tpoint_NXPH3_Tns/Blk3	56.7628278	55.7627869	59.3188641	NA	NA	NA	
beta_tpoint_Glut_NXPH3_right_X_Tns/Blk3	-2.8611174	-7.6385654	-0.3225524	0.0064058	1.6206586	0.0001976	ns

Continued on next page

parameter	estimate	CI.low	CI.high	p.value	p.value.adj	alpha.adj	sig
beta_tpoint_Glut_NXPH3_18_X_Tns/Blk3	-7.9547732	-11.3541734	-4.2747198	0.0000000	0.0000000	0.0001529	***
beta_tpoint_Glut_NXPH3_right18_X_Tns/Blk3	10.8509025	7.5215007	14.8377629	0.0000000	0.0000000	0.0001534	***
baseline_Glut_tpoint_NXPH3_Tns/Blk4	69.1305487	66.6174533	70.0813126	NA	NA	NA	
beta_tpoint_Glut_NXPH3_right_X_Tns/Blk4	-5.4374350	-8.4259843	-0.9900300	0.0000000	0.0000000	0.0001538	***
beta_tpoint_Glut_NXPH3_18_X_Tns/Blk4	-7.2730405	-10.6678922	-3.8417522	0.0000000	0.0000000	0.0001543	***
beta_tpoint_Glut_NXPH3_right18_X_Tns/Blk4	11.3005301	7.3184826	15.9704346	0.0000000	0.0000000	0.0001548	***
baseline_Glut_tpoint_RORB_Tns/Blk1	18.2716619	17.8401623	22.2045080	NA	NA	NA	
beta_tpoint_Glut_RORB_right_X_Tns/Blk1	5.3082774	-0.9036767	10.2338000	0.0008007	0.2394155	0.0001672	ns
beta_tpoint_Glut_RORB_18_X_Tns/Blk1	1.5083452	-3.0578565	6.3035938	0.1999800	27.5972375	0.0003623	ns
beta_tpoint_Glut_RORB_right18_X_Tns/Blk1	-6.2288576	-12.0389725	-2.2187578	0.0000000	0.0000000	0.0001553	***
baseline_Glut_tpoint_RORB_Tns/Blk2	64.2963919	63.2886670	65.6041551	NA	NA	NA	
beta_tpoint_Glut_RORB_right_X_Tns/Blk2	2.0895339	-1.6394259	4.0691236	0.0082074	2.0272245	0.0002024	ns
beta_tpoint_Glut_RORB_18_X_Tns/Blk2	-0.2939379	-2.1693821	2.2377828	0.6333700	38.0022020	0.0008333	ns
beta_tpoint_Glut_RORB_right18_X_Tns/Blk2	-0.6917517	-2.8226900	1.8602682	0.2607347	32.0703633	0.0004065	ns
baseline_Glut_tpoint_RORB_Tns/Blk3	84.7805733	83.6319724	85.4236042	NA	NA	NA	
beta_tpoint_Glut_RORB_right_X_Tns/Blk3	1.1852808	-1.1479045	3.0816529	0.0339305	6.8200380	0.0002488	ns
beta_tpoint_Glut_RORB_18_X_Tns/Blk3	1.8502133	0.2523063	3.9710752	0.0005005	0.1531378	0.0001634	ns
beta_tpoint_Glut_RORB_right18_X_Tns/Blk3	-1.6063503	-3.6939718	0.8953225	0.0031028	0.8377540	0.0001852	ns
baseline_Glut_tpoint_SATB2_Tns/Blk1	57.3564106	56.5506443	60.1223076	NA	NA	NA	
beta_tpoint_Glut_SATB2_right_X_Tns/Blk1	0.2460073	-1.3584627	3.0402963	0.7621860	27.4386948	0.0013889	ns
beta_tpoint_Glut_SATB2_18_X_Tns/Blk1	-0.3645015	-3.1780423	3.2639761	0.6962266	36.2037834	0.0009615	ns
beta_tpoint_Glut_SATB2_right18_X_Tns/Blk1	-4.1008125	-6.5105989	-0.5223777	0.0000000	0.0000000	0.0001558	***
baseline_Glut_tpoint_SATB2_Tns/Blk2	68.9007834	67.7199519	70.2298571	NA	NA	NA	
beta_tpoint_Glut_SATB2_right_X_Tns/Blk2	-0.0037548	-1.4518751	1.3216717	0.9933941	2.9801822	0.0166667	ns
beta_tpoint_Glut_SATB2_18_X_Tns/Blk2	1.1441842	-0.7963236	3.0799380	0.0141127	3.2600340	0.0002165	ns
beta_tpoint_Glut_SATB2_right18_X_Tns/Blk2	0.2954460	-0.5480026	2.6453880	0.2048844	27.8642779	0.0003676	ns
wfactor_point_1_X_BCL11B	0.1957735	-0.0762296	0.3751050	NA	NA	NA	
wfactor_rate_1_X_BCL11B	0.4096582	0.4075888	0.4517024	NA	NA	NA	
wfactor_slope_1_X_BCL11B	1.3684508	-0.0003540	4.0489939	NA	NA	NA	
wfactor_point_2_X_BCL11B	0.4419379	0.2476976	0.4965498	NA	NA	NA	
wfactor_rate_2_X_BCL11B	0.1909389	0.1952612	0.2616401	NA	NA	NA	
wfactor_slope_2_X_BCL11B	0.1478695	-0.6991307	1.1083283	NA	NA	NA	
wfactor_point_3_X_BCL11B	-0.1866719	-0.3805763	0.2259148	NA	NA	NA	
wfactor_rate_3_X_BCL11B	-0.3171182	-0.2883025	-0.2019878	NA	NA	NA	
wfactor_slope_3_X_BCL11B	3.0372763	0.0016876	3.6627798	NA	NA	NA	
wfactor_point_4_X_BCL11B	-0.2402060	-0.3597254	-0.1167183	NA	NA	NA	
wfactor_rate_4_X_BCL11B	0.1362498	0.1403620	0.2265600	NA	NA	NA	
wfactor_slope_4_X_BCL11B	0.3895501	-0.5258025	1.9642040	NA	NA	NA	
wfactor_point_1_X_CUX2	0.2106245	-0.0633195	0.2666532	NA	NA	NA	
wfactor_rate_1_X_CUX2	0.4533587	0.4449342	0.4891432	NA	NA	NA	
wfactor_slope_1_X_CUX2	0.5055620	-0.0394214	3.3145098	NA	NA	NA	
wfactor_point_2_X_CUX2	0.3027104	0.1390669	0.3417193	NA	NA	NA	
wfactor_rate_2_X_CUX2	0.2273501	0.2330629	0.3045524	NA	NA	NA	
wfactor_slope_2_X_CUX2	0.3451819	-0.0957590	0.9368429	NA	NA	NA	
wfactor_point_3_X_CUX2	-0.1126906	-0.3528165	0.2338833	NA	NA	NA	
wfactor_rate_3_X_CUX2	-0.3102639	-0.2897402	-0.1953891	NA	NA	NA	
wfactor_slope_3_X_CUX2	0.4509449	-0.5898241	1.3933659	NA	NA	NA	
wfactor_point_4_X_CUX2	-0.1237797	-0.2798428	0.1091537	NA	NA	NA	
wfactor_rate_4_X_CUX2	0.2025354	0.2102965	0.2851056	NA	NA	NA	
wfactor_slope_4_X_CUX2	0.5481008	-0.0215732	1.0149538	NA	NA	NA	
wfactor_point_1_X_FEZF2	0.4151236	0.2386164	0.4741565	NA	NA	NA	
wfactor_rate_1_X_FEZF2	0.3711262	0.3781923	0.4326327	NA	NA	NA	
wfactor_slope_1_X_FEZF2	1.4060261	0.4757549	4.0534687	NA	NA	NA	
wfactor_point_2_X_FEZF2	0.3060060	0.1860189	0.3940533	NA	NA	NA	
wfactor_rate_2_X_FEZF2	0.3262648	0.3270821	0.3809042	NA	NA	NA	
wfactor_slope_2_X_FEZF2	0.0569510	-0.4321151	0.4965687	NA	NA	NA	
wfactor_point_3_X_FEZF2	-0.2973078	-0.4176180	0.1444885	NA	NA	NA	
wfactor_rate_3_X_FEZF2	-0.3326089	-0.3501099	-0.2779321	NA	NA	NA	
wfactor_slope_3_X_FEZF2	2.8091109	0.4583059	4.8714460	NA	NA	NA	

Continued on next page

parameter	estimate	CI.low	CI.high	p.value	p.value.adj	alpha.adj	sig
wfactor_point_4_X_FEZF2	-0.3035278	-0.4025834	-0.1085008	NA	NA	NA	
wfactor_rate_4_X_FEZF2	-0.2487752	-0.2542191	-0.1700491	NA	NA	NA	
wfactor_slope_4_X_FEZF2	0.2310237	-0.2100231	1.3621898	NA	NA	NA	
wfactor_point_1_X_NXPH3	0.3661731	-0.2180914	0.4123379	NA	NA	NA	
wfactor_rate_1_X_NXPH3	0.2634589	0.2742192	0.3499175	NA	NA	NA	
wfactor_slope_1_X_NXPH3	1.3483721	-0.0416174	3.8044299	NA	NA	NA	
wfactor_point_2_X_NXPH3	0.4729384	0.2950833	0.5594846	NA	NA	NA	
wfactor_rate_2_X_NXPH3	0.2641053	0.2567905	0.3223690	NA	NA	NA	
wfactor_slope_2_X_NXPH3	-0.6499028	-0.8290316	0.2921522	NA	NA	NA	
wfactor_point_3_X_NXPH3	0.0414940	-0.3765449	0.1427241	NA	NA	NA	
wfactor_rate_3_X_NXPH3	-0.1817005	-0.1727661	0.1178251	NA	NA	NA	
wfactor_slope_3_X_NXPH3	2.5921999	0.4783415	4.2742298	NA	NA	NA	
wfactor_point_4_X_NXPH3	-0.1713077	-0.3426126	0.2975190	NA	NA	NA	
wfactor_rate_4_X_NXPH3	-0.2335585	-0.2405467	-0.1546202	NA	NA	NA	
wfactor_slope_4_X_NXPH3	1.2924777	0.6333236	3.9238424	NA	NA	NA	
wfactor_point_1_X_RORB	0.1970506	0.0828213	0.3184446	NA	NA	NA	
wfactor_rate_1_X_RORB	0.3621718	0.3626341	0.4092101	NA	NA	NA	
wfactor_slope_1_X_RORB	1.0763760	0.6202989	1.5017511	NA	NA	NA	
wfactor_point_2_X_RORB	0.3404998	0.2583812	0.4508471	NA	NA	NA	
wfactor_rate_2_X_RORB	0.3130246	0.3041817	0.3564764	NA	NA	NA	
wfactor_slope_2_X_RORB	-0.0012385	-0.4963269	0.0423975	NA	NA	NA	
wfactor_point_3_X_RORB	-0.2879841	-0.3563992	-0.0654765	NA	NA	NA	
wfactor_rate_3_X_RORB	-0.2754207	-0.2863402	-0.2082055	NA	NA	NA	
wfactor_slope_3_X_RORB	0.4520232	-0.0110077	1.4474647	NA	NA	NA	
wfactor_point_4_X_RORB	-0.1804568	-0.3237333	0.1148557	NA	NA	NA	
wfactor_rate_4_X_RORB	-0.1008746	-0.1473102	0.0977031	NA	NA	NA	
wfactor_slope_4_X_RORB	0.4302122	0.2459419	0.7723996	NA	NA	NA	
wfactor_point_1_X_SATB2	0.0612697	-0.1803762	0.3195841	NA	NA	NA	
wfactor_rate_1_X_SATB2	0.4017666	0.3934254	0.4121785	NA	NA	NA	
wfactor_slope_1_X_SATB2	2.9998158	1.1600824	3.4254482	NA	NA	NA	
wfactor_point_2_X_SATB2	-0.2069643	-0.4668012	0.3110745	NA	NA	NA	
wfactor_rate_2_X_SATB2	0.3297969	0.3211107	0.3471511	NA	NA	NA	
wfactor_slope_2_X_SATB2	1.1033578	0.2445203	2.4961001	NA	NA	NA	
wfactor_point_3_X_SATB2	-0.3620098	-0.4225665	0.2205927	NA	NA	NA	
wfactor_rate_3_X_SATB2	-0.3490620	-0.3532959	-0.3321783	NA	NA	NA	
wfactor_slope_3_X_SATB2	3.3290203	1.5533349	3.3912018	NA	NA	NA	
wfactor_point_4_X_SATB2	0.1354875	-0.3454724	0.4899694	NA	NA	NA	
wfactor_rate_4_X_SATB2	-0.2790832	-0.2705705	-0.2310511	NA	NA	NA	
wfactor_slope_4_X_SATB2	2.3774543	2.3151875	4.2633268	NA	NA	NA	

Table S2: Parameter estimates for radial liver model.

parameter	estimate	CI.low	CI.high	p.value	p.value.adj	alpha.adj	sig
baseline_hepatocytes_Rt_ARNTL_Tns/Blk1	2.5154809	2.1378509	2.8407857	NA	NA	NA	
beta_Rt_hepatocytes_ARNTL_6_X_Tns/Blk1	-1.5419031	-2.5314522	2.5216302	0.0025997	0.1273873	0.0010204	ns
beta_Rt_hepatocytes_ARNTL_12_X_Tns/Blk1	-0.2014482	-1.5775323	1.0494103	0.5133487	4.6201380	0.0055556	ns
beta_Rt_hepatocytes_ARNTL_18_X_Tns/Blk1	0.4589272	-1.7723064	1.7674749	0.1882812	4.1421858	0.0022727	ns
baseline_hepatocytes_Rt_ARNTL_Tns/Blk2	1.9612137	1.5865064	2.2501951	NA	NA	NA	
beta_Rt_hepatocytes_ARNTL_6_X_Tns/Blk2	-0.9570678	-1.6727981	0.1448553	0.0007999	0.0447955	0.0008929	*
beta_Rt_hepatocytes_ARNTL_12_X_Tns/Blk2	-0.2330649	-1.0278183	0.4190566	0.3134687	4.7020298	0.0033333	ns
beta_Rt_hepatocytes_ARNTL_18_X_Tns/Blk2	1.0302878	0.0743575	1.6507168	0.0000000	0.0000000	0.0005556	***
baseline_hepatocytes_Rt_ASS1_Tns/Blk1	4.4383673	4.2395413	4.6115714	NA	NA	NA	
beta_Rt_hepatocytes_ASS1_6_X_Tns/Blk1	0.1165141	-0.3697519	0.5133654	0.4576542	4.5765423	0.0050000	ns
beta_Rt_hepatocytes_ASS1_12_X_Tns/Blk1	-0.8803818	-1.5657711	-0.2705535	0.0000000	0.0000000	0.0005618	***
beta_Rt_hepatocytes_ASS1_18_X_Tns/Blk1	-0.1274763	-0.7803929	0.4570407	0.5517448	4.4139586	0.0062500	ns
baseline_hepatocytes_Rt_ASS1_Tns/Blk2	5.6159978	5.5416187	5.6924091	NA	NA	NA	
beta_Rt_hepatocytes_ASS1_6_X_Tns/Blk2	-0.1080933	-0.3165550	0.0988700	0.1248875	3.3719628	0.0018519	ns

Continued on next page

parameter	estimate	CI.low	CI.high	p.value	p.value.adj	alpha.adj	sig
beta_Rt_hepatocytes_ASS1_12_X_Tns/Blk2	-0.7375412	-1.0120023	-0.4007524	0.0000000	0.0000000	0.0005682	***
beta_Rt_hepatocytes_ASS1_18_X_Tns/Blk2	0.1488039	-0.3067997	0.4085657	0.1455854	3.4940506	0.0020833	ns
baseline_hepatocytes_Rt_ASS1_Tns/Blk3	5.8215735	5.7697381	5.8834851	NA	NA	NA	
beta_Rt_hepatocytes_ASS1_6_X_Tns/Blk3	-0.3069583	-0.5154171	-0.1320086	0.0000000	0.0000000	0.0005747	***
beta_Rt_hepatocytes_ASS1_12_X_Tns/Blk3	-0.3338955	-0.5483124	-0.1136495	0.0000000	0.0000000	0.0005814	***
beta_Rt_hepatocytes_ASS1_18_X_Tns/Blk3	0.2145101	0.0252618	0.4376682	0.0007999	0.0439956	0.0009091	*
baseline_hepatocytes_Rt_DBP_Tns/Blk1	0.9934356	0.0683945	2.7835837	NA	NA	NA	
beta_Rt_hepatocytes_DBP_6_X_Tns/Blk1	2.7277489	-1.7500567	3.9628536	0.0113989	0.5243476	0.0010870	ns
beta_Rt_hepatocytes_DBP_12_X_Tns/Blk1	-0.4924287	-1.6294130	0.5114801	0.1368863	3.5590441	0.0019231	ns
beta_Rt_hepatocytes_DBP_18_X_Tns/Blk1	-2.1294364	-3.6366720	-0.3410873	0.0001000	0.0059994	0.0008333	**
baseline_hepatocytes_Rt_DBP_Tns/Blk2	0.7417403	0.6172125	1.0725330	NA	NA	NA	
beta_Rt_hepatocytes_DBP_6_X_Tns/Blk2	3.0168703	2.1328275	3.3504608	0.0000000	0.0000000	0.0005882	***
beta_Rt_hepatocytes_DBP_12_X_Tns/Blk2	-0.1977914	-0.6350780	0.1430240	0.1075892	3.0124988	0.0017857	ns
beta_Rt_hepatocytes_DBP_18_X_Tns/Blk2	-1.9303535	-2.6695556	-1.1418855	0.0000000	0.0000000	0.0005952	***
baseline_hepatocytes_Rt_ELOVL3_Tns/Blk1	5.4642766	5.3861750	5.6279720	NA	NA	NA	
beta_Rt_hepatocytes_ELOVL3_6_X_Tns/Blk1	-1.4173029	-1.8512055	-0.7019891	0.0000000	0.0000000	0.0006024	***
beta_Rt_hepatocytes_ELOVL3_12_X_Tns/Blk1	-1.1998421	-2.2740401	-0.7028458	0.0000000	0.0000000	0.0006098	***
beta_Rt_hepatocytes_ELOVL3_18_X_Tns/Blk1	1.2701694	0.7816253	2.0933791	0.0000000	0.0000000	0.0006173	***
baseline_hepatocytes_Rt_ELOVL3_Tns/Blk2	4.6897536	4.4646333	4.7905597	NA	NA	NA	
beta_Rt_hepatocytes_ELOVL3_6_X_Tns/Blk2	-1.5801278	-2.6961897	-1.0736146	0.0000000	0.0000000	0.0006250	***
beta_Rt_hepatocytes_ELOVL3_12_X_Tns/Blk2	-1.5633093	-2.1367486	-0.2629568	0.0000000	0.0000000	0.0006329	***
beta_Rt_hepatocytes_ELOVL3_18_X_Tns/Blk2	2.2424833	1.5408468	2.8742597	0.0000000	0.0000000	0.0006410	***
baseline_hepatocytes_Rt_GLUL_Tns/Blk1	6.6888575	6.1490227	6.9675951	NA	NA	NA	
beta_Rt_hepatocytes_GLUL_6_X_Tns/Blk1	-0.7985458	-1.9775633	0.4314883	0.0287971	1.0942906	0.0013158	ns
beta_Rt_hepatocytes_GLUL_12_X_Tns/Blk1	0.8275158	-0.2795182	2.1031680	0.0188981	0.7748225	0.0012195	ns
beta_Rt_hepatocytes_GLUL_18_X_Tns/Blk1	-1.6182940	-2.8100718	-0.4914945	0.0000000	0.0000000	0.0006494	***
baseline_hepatocytes_Rt_GLUL_Tns/Blk2	3.0815333	2.5381604	3.4250672	NA	NA	NA	
beta_Rt_hepatocytes_GLUL_6_X_Tns/Blk2	-1.0490501	-2.1114680	0.1306592	0.0024998	0.1249875	0.0010000	ns
beta_Rt_hepatocytes_GLUL_12_X_Tns/Blk2	1.4320070	0.4027434	2.2659974	0.0000000	0.0000000	0.0006579	***
beta_Rt_hepatocytes_GLUL_18_X_Tns/Blk2	0.2170366	-0.4895279	0.8533154	0.2456754	4.6678332	0.0026316	ns
baseline_hepatocytes_Rt_PCK1_Tns/Blk1	4.9425614	4.7257184	5.0675735	NA	NA	NA	
beta_Rt_hepatocytes_PCK1_6_X_Tns/Blk1	0.5868436	0.1998327	1.0404040	0.0000000	0.0000000	0.0006667	***
beta_Rt_hepatocytes_PCK1_12_X_Tns/Blk1	0.3047315	-0.0887041	0.8076634	0.0280972	1.0957904	0.0012821	ns
beta_Rt_hepatocytes_PCK1_18_X_Tns/Blk1	-1.5581314	-2.3051952	-1.0889329	0.0000000	0.0000000	0.0006757	***
baseline_hepatocytes_Rt_PCK1_Tns/Blk2	5.8288414	5.7356108	5.8807488	NA	NA	NA	
beta_Rt_hepatocytes_PCK1_6_X_Tns/Blk2	0.3584047	0.2045067	0.5759059	0.0000000	0.0000000	0.0006849	***
beta_Rt_hepatocytes_PCK1_12_X_Tns/Blk2	0.2528947	0.0561065	0.4187415	0.0000000	0.0000000	0.0006944	***
beta_Rt_hepatocytes_PCK1_18_X_Tns/Blk2	-1.2432488	-1.5474223	-0.9544442	0.0000000	0.0000000	0.0007042	***
baseline_hepatocytes_Rt_PCK1_Tns/Blk3	6.0777154	5.9953700	6.1089191	NA	NA	NA	
beta_Rt_hepatocytes_PCK1_6_X_Tns/Blk3	0.2572533	0.1147781	0.4665592	0.0000000	0.0000000	0.0007143	***
beta_Rt_hepatocytes_PCK1_12_X_Tns/Blk3	0.3221156	0.1443146	0.4802646	0.0000000	0.0000000	0.0007246	***
beta_Rt_hepatocytes_PCK1_18_X_Tns/Blk3	-0.7187321	-0.8845862	-0.5848292	0.0000000	0.0000000	0.0007353	***
baseline_hepatocytes_tslope_ARNTL_Tns/Blk1	8.4081554	1.5598422	15.1484517	NA	NA	NA	
beta_tslope_hepatocytes_ARNTL_6_X_Tns/Blk1	4.1845781	-4.0165301	13.3190428	0.0280972	1.1238876	0.0012500	ns
beta_tslope_hepatocytes_ARNTL_12_X_Tns/Blk1	2.1294133	-4.0094231	8.8354952	0.0905909	2.7177282	0.0016667	ns
beta_tslope_hepatocytes_ARNTL_18_X_Tns/Blk1	2.3800506	-6.6137405	6.1196821	0.0548945	2.0310969	0.0013514	ns
baseline_hepatocytes_tslope_ASS1_Tns/Blk1	10.8796313	7.9624702	14.8777677	NA	NA	NA	
beta_tslope_hepatocytes_ASS1_6_X_Tns/Blk1	4.7041836	0.7898985	8.5561760	0.0004000	0.0231977	0.0008621	*
beta_tslope_hepatocytes_ASS1_12_X_Tns/Blk1	3.1523780	-1.5951849	6.1941653	0.0019998	0.1039896	0.0009615	ns
beta_tslope_hepatocytes_ASS1_18_X_Tns/Blk1	1.9275966	-0.0292144	4.1240714	0.0031997	0.1535846	0.0010417	ns
baseline_hepatocytes_tslope_ASS1_Tns/Blk2	7.7737812	5.6924040	9.8376759	NA	NA	NA	
beta_tslope_hepatocytes_ASS1_6_X_Tns/Blk2	2.9823327	0.3854623	5.6934171	0.0001000	0.0058994	0.0008475	**
beta_tslope_hepatocytes_ASS1_12_X_Tns/Blk2	0.7281924	-0.9567818	3.3544711	0.2785721	5.0142986	0.0027778	ns
beta_tslope_hepatocytes_ASS1_18_X_Tns/Blk2	0.7517899	-0.7685353	1.8140524	0.0819918	2.5417458	0.0016129	ns
baseline_hepatocytes_tslope_DBP_Tns/Blk1	9.0966011	1.4438649	13.0538906	NA	NA	NA	
beta_tslope_hepatocytes_DBP_6_X_Tns/Blk1	4.5980220	-1.3738506	11.1085362	0.0136986	0.6027397	0.0011364	ns
beta_tslope_hepatocytes_DBP_12_X_Tns/Blk1	2.6696840	-3.6586261	7.6553455	0.1440856	3.6021398	0.0020000	ns
beta_tslope_hepatocytes_DBP_18_X_Tns/Blk1	0.5150447	-9.5813015	3.2734695	0.6916308	2.7665233	0.0125000	ns
baseline_hepatocytes_tslope_ELOVL3_Tns/Blk1	3.2186245	1.1357329	8.6735418	NA	NA	NA	

Continued on next page

parameter	estimate	CI.low	CI.high	p.value	p.value.adj	alpha.adj	sig
beta_tslope_hepatocytes_ELOVL3_6_X_Tns/Blk1	1.3616800	-2.1653311	9.5110577	0.7078292	2.1234877	0.0166667	ns
beta_tslope_hepatocytes_ELOVL3_12_X_Tns/Blk1	-3.1906116	-8.6698825	9.2503467	0.2844716	4.5515448	0.0031250	ns
beta_tslope_hepatocytes_ELOVL3_18_X_Tns/Blk1	2.4676085	-1.7564842	8.3913424	0.1066893	3.0939906	0.0017241	ns
baseline_hepatocytes_tslope_GLUL_Tns/Blk1	0.5925004	0.5024827	0.8769942	NA	NA	NA	
beta_tslope_hepatocytes_GLUL_6_X_Tns/Blk1	0.2297126	-0.2774245	5.0456307	0.3627637	4.3531647	0.0041667	ns
beta_tslope_hepatocytes_GLUL_12_X_Tns/Blk1	0.3956640	-5.1551382	2.6688711	0.1524848	3.5071493	0.0021739	ns
beta_tslope_hepatocytes_GLUL_18_X_Tns/Blk1	-0.1077728	-0.7742099	6.6438864	0.9827017	0.9827017	0.0500000	ns
baseline_hepatocytes_tslope_PCK1_Tns/Blk1	9.0950321	6.7115810	11.7653995	NA	NA	NA	
beta_tslope_hepatocytes_PCK1_6_X_Tns/Blk1	4.3585017	2.1736337	7.3846600	0.0000000	0.0000000	0.0007463	***
beta_tslope_hepatocytes_PCK1_12_X_Tns/Blk1	3.0294817	1.4132318	5.6151063	0.0000000	0.0000000	0.0007576	***
beta_tslope_hepatocytes_PCK1_18_X_Tns/Blk1	1.9603607	0.7439186	4.0666166	0.0010999	0.0593941	0.0009259	ns
baseline_hepatocytes_tslope_PCK1_Tns/Blk2	7.9875763	5.7314549	10.0725955	NA	NA	NA	
beta_tslope_hepatocytes_PCK1_6_X_Tns/Blk2	2.5619977	1.1956797	5.3495153	0.0006999	0.0398960	0.0008772	*
beta_tslope_hepatocytes_PCK1_12_X_Tns/Blk2	2.7287767	0.6297040	3.8978940	0.0000000	0.0000000	0.0007692	***
beta_tslope_hepatocytes_PCK1_18_X_Tns/Blk2	0.8978254	-0.1349144	2.0948198	0.0058994	0.2772723	0.0010638	ns
baseline_hepatocytes_tpoint_ARNTL_Tns/Blk1	4.3101213	3.1070702	5.2002672	NA	NA	NA	
beta_tpoint_hepatocytes_ARNTL_6_X_Tns/Blk1	-0.9888071	-2.3029341	1.3307071	0.0654935	2.1612839	0.0015152	ns
beta_tpoint_hepatocytes_ARNTL_12_X_Tns/Blk1	0.4307623	-1.2639245	1.4891289	0.2205779	4.4115588	0.0025000	ns
beta_tpoint_hepatocytes_ARNTL_18_X_Tns/Blk1	-1.0684983	-2.2410187	0.6411015	0.0186981	0.7853215	0.0011905	ns
baseline_hepatocytes_tpoint_ASS1_Tns/Blk1	1.8674828	1.8286588	1.9115375	NA	NA	NA	
beta_tpoint_hepatocytes_ASS1_6_X_Tns/Blk1	0.0715473	-0.0027166	0.2171832	0.0167983	0.7223278	0.0011628	ns
beta_tpoint_hepatocytes_ASS1_12_X_Tns/Blk1	-0.0113085	-0.0987905	0.0545347	0.7256274	1.4512549	0.0250000	ns
beta_tpoint_hepatocytes_ASS1_18_X_Tns/Blk1	0.0169608	-0.0933869	0.0949753	0.6047395	3.0236976	0.0100000	ns
baseline_hepatocytes_tpoint_ASS1_Tns/Blk2	4.3216551	4.2672241	4.4268716	NA	NA	NA	
beta_tpoint_hepatocytes_ASS1_6_X_Tns/Blk2	0.6084031	0.3805430	0.9412878	0.0000000	0.0000000	0.0007813	***
beta_tpoint_hepatocytes_ASS1_12_X_Tns/Blk2	-0.0752031	-0.2749624	0.2427179	0.3937606	4.3313669	0.0045455	ns
beta_tpoint_hepatocytes_ASS1_18_X_Tns/Blk2	-0.3655548	-1.0785895	0.1465881	0.0789921	2.5277472	0.0015625	ns
baseline_hepatocytes_tpoint_DBP_Tns/Blk1	3.0104110	1.8918754	3.4104240	NA	NA	NA	
beta_tpoint_hepatocytes_DBP_6_X_Tns/Blk1	0.1626413	-1.3467894	1.0944384	0.5611439	3.3668633	0.0083333	ns
beta_tpoint_hepatocytes_DBP_12_X_Tns/Blk1	-0.3125639	-1.2857399	0.8968228	0.3588641	4.6652335	0.0038462	ns
beta_tpoint_hepatocytes_DBP_18_X_Tns/Blk1	1.2366651	-0.7644024	2.4271083	0.0592941	2.1345865	0.0013889	ns
baseline_hepatocytes_tpoint_ELOVL3_Tns/Blk1	5.0648458	4.6215795	5.3788768	NA	NA	NA	
beta_tpoint_hepatocytes_ELOVL3_6_X_Tns/Blk1	-0.8510328	-1.3058929	0.1724514	0.0015998	0.0847915	0.0009434	ns
beta_tpoint_hepatocytes_ELOVL3_12_X_Tns/Blk1	-0.2614321	-1.5032201	0.7045481	0.5595440	3.9168083	0.0071429	ns
beta_tpoint_hepatocytes_ELOVL3_18_X_Tns/Blk1	0.5059601	-0.3738233	2.1189253	0.2144786	4.5040496	0.0023810	ns
baseline_hepatocytes_tpoint_GLUL_Tns/Blk1	3.3255502	3.1381229	4.0502323	NA	NA	NA	
beta_tpoint_hepatocytes_GLUL_6_X_Tns/Blk1	0.6257468	-0.5300116	1.5182233	0.0641936	2.1825817	0.0014706	ns
beta_tpoint_hepatocytes_GLUL_12_X_Tns/Blk1	-0.7600252	-1.6449293	0.2201387	0.0129987	0.5849415	0.0011111	ns
beta_tpoint_hepatocytes_GLUL_18_X_Tns/Blk1	-0.6677659	-1.2198757	0.2443394	0.0022998	0.1172883	0.0009804	ns
baseline_hepatocytes_tpoint_PCK1_Tns/Blk1	1.8982930	1.8245427	1.9321560	NA	NA	NA	
beta_tpoint_hepatocytes_PCK1_6_X_Tns/Blk1	0.0820904	-0.0213114	0.2802225	0.0638936	2.2362764	0.0014286	ns
beta_tpoint_hepatocytes_PCK1_12_X_Tns/Blk1	-0.0418103	-0.2401329	0.1772968	0.3384662	4.7385261	0.0035714	ns
beta_tpoint_hepatocytes_PCK1_18_X_Tns/Blk1	-0.0361851	-0.2485961	0.0758779	0.2799720	4.7595240	0.0029412	ns
baseline_hepatocytes_tpoint_PCK1_Tns/Blk2	4.5303607	4.4369476	4.5932380	NA	NA	NA	
beta_tpoint_hepatocytes_PCK1_6_X_Tns/Blk2	0.6417429	0.3725273	0.7631565	0.0000000	0.0000000	0.0007937	***
beta_tpoint_hepatocytes_PCK1_12_X_Tns/Blk2	-0.7709979	-0.9197212	-0.3935672	0.0000000	0.0000000	0.0008065	***
beta_tpoint_hepatocytes_PCK1_18_X_Tns/Blk2	-0.4094512	-0.6576463	-0.2061227	0.0000000	0.0000000	0.0008197	***
wfactor_point_1_X_ARNTL	0.6469026	-0.6240158	0.8434142	NA	NA	NA	
wfactor_rate_1_X_ARNTL	-0.0830733	-0.3185747	0.4299959	NA	NA	NA	
wfactor_slope_1_X_ARNTL	0.0485501	-0.6320436	5.9600055	NA	NA	NA	
wfactor_point_10_X_ARNTL	-0.7114774	-0.8765728	0.3894421	NA	NA	NA	
wfactor_rate_10_X_ARNTL	-0.0841030	-0.2474691	0.6958559	NA	NA	NA	
wfactor_slope_10_X_ARNTL	1.1934161	-0.1232053	3.9898079	NA	NA	NA	
wfactor_point_2_X_ARNTL	0.7147404	-0.5904358	0.9836398	NA	NA	NA	
wfactor_rate_2_X_ARNTL	0.1957111	-0.2562844	0.4487307	NA	NA	NA	
wfactor_slope_2_X_ARNTL	0.0129097	-1.7301537	3.2568829	NA	NA	NA	
wfactor_point_3_X_ARNTL	-0.2934669	-0.8551082	0.7022624	NA	NA	NA	
wfactor_rate_3_X_ARNTL	-0.3052308	-0.4241732	0.3205884	NA	NA	NA	
wfactor_slope_3_X_ARNTL	2.1103433	-0.1467471	1.5849417	NA	NA	NA	

Continued on next page

parameter	estimate	CI.low	CI.high	p.value	p.value.adj	alpha.adj	sig
wfactor_point_4_X_ARNTL	-0.1084521	-0.8302644	0.6917587	NA	NA	NA	
wfactor_rate_4_X_ARNTL	0.5590269	0.4209250	0.8295214	NA	NA	NA	
wfactor_slope_4_X_ARNTL	0.0194391	-0.1870650	0.4830498	NA	NA	NA	
wfactor_point_5_X_ARNTL	-0.3347479	-0.7647754	0.6943710	NA	NA	NA	
wfactor_rate_5_X_ARNTL	0.4245800	-0.2840159	2.0064681	NA	NA	NA	
wfactor_slope_5_X_ARNTL	0.1802757	-0.0402139	0.1579100	NA	NA	NA	
wfactor_point_6_X_ARNTL	-0.2977180	-0.8216865	0.6142628	NA	NA	NA	
wfactor_rate_6_X_ARNTL	0.6017844	0.2726160	3.7655785	NA	NA	NA	
wfactor_slope_6_X_ARNTL	0.3277990	-0.2611688	0.2156895	NA	NA	NA	
wfactor_point_7_X_ARNTL	-0.0414086	-0.7657510	0.6765417	NA	NA	NA	
wfactor_rate_7_X_ARNTL	0.8587393	0.6420033	1.2175339	NA	NA	NA	
wfactor_slope_7_X_ARNTL	0.0027176	-0.0380469	1.3473611	NA	NA	NA	
wfactor_point_8_X_ARNTL	-0.0038809	-0.7761458	0.6819511	NA	NA	NA	
wfactor_rate_8_X_ARNTL	0.6215661	0.3523346	5.0062753	NA	NA	NA	
wfactor_slope_8_X_ARNTL	0.4276858	0.0004017	0.0310615	NA	NA	NA	
wfactor_point_9_X_ARNTL	0.4728230	-0.7818561	0.9066829	NA	NA	NA	
wfactor_rate_9_X_ARNTL	0.2582050	0.0636539	0.7395443	NA	NA	NA	
wfactor_slope_9_X_ARNTL	0.1340724	-0.1871159	5.0082468	NA	NA	NA	
wfactor_point_1_X_ASS1	-0.0065768	-0.1488647	0.2412787	NA	NA	NA	
wfactor_rate_1_X_ASS1	-0.1842158	-0.2242090	-0.1553572	NA	NA	NA	
wfactor_slope_1_X_ASS1	0.1623526	0.0345966	1.6272521	NA	NA	NA	
wfactor_point_10_X_ASS1	0.1570335	-0.0195080	0.2369043	NA	NA	NA	
wfactor_rate_10_X_ASS1	-0.0958226	-0.1476372	0.0953487	NA	NA	NA	
wfactor_slope_10_X_ASS1	2.2419770	1.1623523	4.8301466	NA	NA	NA	
wfactor_point_2_X_ASS1	0.2775528	0.0378342	0.2509525	NA	NA	NA	
wfactor_rate_2_X_ASS1	0.2105745	0.1795366	0.2339312	NA	NA	NA	
wfactor_slope_2_X_ASS1	2.1618079	0.0606772	1.0091264	NA	NA	NA	
wfactor_point_3_X_ASS1	0.2127536	-0.0269208	0.2802734	NA	NA	NA	
wfactor_rate_3_X_ASS1	0.0946741	-0.0099474	0.1509032	NA	NA	NA	
wfactor_slope_3_X_ASS1	0.7682775	0.0317239	0.8147541	NA	NA	NA	
wfactor_point_4_X_ASS1	0.1724362	-0.1602571	0.1868410	NA	NA	NA	
wfactor_rate_4_X_ASS1	0.2316952	0.2095644	0.2594754	NA	NA	NA	
wfactor_slope_4_X_ASS1	1.5885486	0.0664074	0.5797198	NA	NA	NA	
wfactor_point_5_X_ASS1	0.2002061	0.0677866	0.3170773	NA	NA	NA	
wfactor_rate_5_X_ASS1	-0.2223684	-0.2556315	-0.1956500	NA	NA	NA	
wfactor_slope_5_X_ASS1	0.4209945	0.2362527	2.0668960	NA	NA	NA	
wfactor_point_6_X_ASS1	-0.0680543	-0.1821587	0.2395774	NA	NA	NA	
wfactor_rate_6_X_ASS1	-0.3651208	-0.4070823	-0.3336678	NA	NA	NA	
wfactor_slope_6_X_ASS1	0.0253501	-0.0627752	3.5809343	NA	NA	NA	
wfactor_point_7_X_ASS1	0.1043368	-0.1564539	0.2304968	NA	NA	NA	
wfactor_rate_7_X_ASS1	0.3563259	0.3341365	0.3863424	NA	NA	NA	
wfactor_slope_7_X_ASS1	0.1230829	0.0382693	0.7548888	NA	NA	NA	
wfactor_point_8_X_ASS1	0.2171615	0.0083509	0.3102482	NA	NA	NA	
wfactor_rate_8_X_ASS1	0.1579296	0.0168301	0.2144385	NA	NA	NA	
wfactor_slope_8_X_ASS1	0.3640597	0.0654802	1.2654769	NA	NA	NA	
wfactor_point_9_X_ASS1	0.1580183	-0.0549792	0.2514429	NA	NA	NA	
wfactor_rate_9_X_ASS1	0.0949864	-0.0850475	0.1587435	NA	NA	NA	
wfactor_slope_9_X_ASS1	0.6820062	-0.0035636	2.0200194	NA	NA	NA	
wfactor_point_1_X_DBP	-0.6364480	-0.6978369	0.6801195	NA	NA	NA	
wfactor_rate_1_X_DBP	0.7118659	0.4043266	4.5109124	NA	NA	NA	
wfactor_slope_1_X_DBP	0.6643627	0.1511915	1.5633088	NA	NA	NA	
wfactor_point_10_X_DBP	-0.2466268	-0.6644576	0.7881980	NA	NA	NA	
wfactor_rate_10_X_DBP	0.5224217	0.2939128	0.9306069	NA	NA	NA	
wfactor_slope_10_X_DBP	0.8940873	-0.7775591	3.4035492	NA	NA	NA	
wfactor_point_2_X_DBP	0.0033323	-0.7306180	0.9089192	NA	NA	NA	
wfactor_rate_2_X_DBP	0.7761884	0.5075558	1.1174150	NA	NA	NA	
wfactor_slope_2_X_DBP	0.1857190	-0.3058407	4.2053005	NA	NA	NA	
wfactor_point_3_X_DBP	-0.3770025	-0.6768795	0.8412812	NA	NA	NA	
wfactor_rate_3_X_DBP	0.9576202	0.7503425	1.3649182	NA	NA	NA	

Continued on next page

parameter	estimate	CI.low	CI.high	p.value	p.value.adj	alpha.adj	sig
wfactor_slope_3_X_DBP	0.0620796	-0.1169019	1.2762007	NA	NA	NA	
wfactor_point_4_X_DBP	-0.7094410	-0.6755976	0.0629019	NA	NA	NA	
wfactor_rate_4_X_DBP	0.2058288	0.0949059	0.2816797	NA	NA	NA	
wfactor_slope_4_X_DBP	0.0894735	0.0507854	1.6038961	NA	NA	NA	
wfactor_point_5_X_DBP	-0.6975884	-0.7119148	0.7254622	NA	NA	NA	
wfactor_rate_5_X_DBP	-0.1984930	-0.2963614	-0.1165637	NA	NA	NA	
wfactor_slope_5_X_DBP	0.0157318	0.0003052	0.0225950	NA	NA	NA	
wfactor_point_6_X_DBP	-0.8350855	-0.8094782	0.5086505	NA	NA	NA	
wfactor_rate_6_X_DBP	-0.5601630	-0.6563738	-0.4400547	NA	NA	NA	
wfactor_slope_6_X_DBP	0.2815071	0.1222942	4.2112187	NA	NA	NA	
wfactor_point_7_X_DBP	-0.7773821	-0.7375287	0.3727047	NA	NA	NA	
wfactor_rate_7_X_DBP	0.5021539	0.4785988	0.5742861	NA	NA	NA	
wfactor_slope_7_X_DBP	0.0105570	-0.2418225	0.4318874	NA	NA	NA	
wfactor_point_8_X_DBP	-0.8033897	-0.7684124	0.3573166	NA	NA	NA	
wfactor_rate_8_X_DBP	-0.1206780	-0.2330646	0.2266186	NA	NA	NA	
wfactor_slope_8_X_DBP	0.0549782	0.0171652	2.3285733	NA	NA	NA	
wfactor_point_9_X_DBP	-0.6381488	-0.6288993	0.7469575	NA	NA	NA	
wfactor_rate_9_X_DBP	0.2321426	-0.2723057	0.7205961	NA	NA	NA	
wfactor_slope_9_X_DBP	0.0975822	-1.0268047	0.2011458	NA	NA	NA	
wfactor_point_1_X_ELOVL3	0.2958157	-0.4427930	0.5818924	NA	NA	NA	
wfactor_rate_1_X_ELOVL3	-0.1698132	-0.2195239	-0.1043559	NA	NA	NA	
wfactor_slope_1_X_ELOVL3	-0.2870310	-0.8572868	0.6123569	NA	NA	NA	
wfactor_point_10_X_ELOVL3	-0.0385393	-0.7297841	0.5637651	NA	NA	NA	
wfactor_rate_10_X_ELOVL3	-0.2692271	-0.3349728	-0.2068624	NA	NA	NA	
wfactor_slope_10_X_ELOVL3	-0.0042495	-0.0944857	2.5374149	NA	NA	NA	
wfactor_point_2_X_ELOVL3	0.0096759	-0.6182440	0.4725309	NA	NA	NA	
wfactor_rate_2_X_ELOVL3	-0.1304923	-0.1907413	0.0568912	NA	NA	NA	
wfactor_slope_2_X_ELOVL3	1.0678088	-0.0612256	2.4577982	NA	NA	NA	
wfactor_point_3_X_ELOVL3	-0.1926442	-0.6890186	0.3582352	NA	NA	NA	
wfactor_rate_3_X_ELOVL3	0.2266738	0.1933058	0.2773228	NA	NA	NA	
wfactor_slope_3_X_ELOVL3	-0.3864524	-1.3692734	0.0805488	NA	NA	NA	
wfactor_point_4_X_ELOVL3	-0.0618432	-0.6766849	0.6442410	NA	NA	NA	
wfactor_rate_4_X_ELOVL3	0.1962341	-0.0704002	0.3201371	NA	NA	NA	
wfactor_slope_4_X_ELOVL3	-0.0540740	-1.0486203	3.7548314	NA	NA	NA	
wfactor_point_5_X_ELOVL3	-0.0094552	-0.5260758	0.5515224	NA	NA	NA	
wfactor_rate_5_X_ELOVL3	-0.2190955	-0.3222877	-0.0074018	NA	NA	NA	
wfactor_slope_5_X_ELOVL3	3.2769307	-0.1870923	6.3512359	NA	NA	NA	
wfactor_point_6_X_ELOVL3	-0.0795846	-0.7766938	0.2608764	NA	NA	NA	
wfactor_rate_6_X_ELOVL3	-0.3208590	-0.4751889	0.4213573	NA	NA	NA	
wfactor_slope_6_X_ELOVL3	3.0615048	-0.2581349	6.3564488	NA	NA	NA	
wfactor_point_7_X_ELOVL3	-0.6053736	-0.9506572	0.3126665	NA	NA	NA	
wfactor_rate_7_X_ELOVL3	0.3465165	0.0069107	0.5769806	NA	NA	NA	
wfactor_slope_7_X_ELOVL3	0.4081984	-1.0419303	4.5039444	NA	NA	NA	
wfactor_point_8_X_ELOVL3	-0.0451892	-0.8143974	0.4945328	NA	NA	NA	
wfactor_rate_8_X_ELOVL3	0.5858038	0.5094390	0.7513595	NA	NA	NA	
wfactor_slope_8_X_ELOVL3	-1.0719645	-1.5454384	3.3063153	NA	NA	NA	
wfactor_point_9_X_ELOVL3	-0.0563390	-0.6463357	0.3748666	NA	NA	NA	
wfactor_rate_9_X_ELOVL3	0.2735998	0.2190243	0.3326194	NA	NA	NA	
wfactor_slope_9_X_ELOVL3	1.2044564	-0.0010739	0.0585967	NA	NA	NA	
wfactor_point_1_X_GLUL	-0.0264045	-0.7123079	0.4165358	NA	NA	NA	
wfactor_rate_1_X_GLUL	-0.2296741	-0.3000025	0.1690378	NA	NA	NA	
wfactor_slope_1_X_GLUL	-0.5602785	-0.7535130	-0.1694922	NA	NA	NA	
wfactor_point_10_X_GLUL	-0.6040119	-0.7944425	0.0942955	NA	NA	NA	
wfactor_rate_10_X_GLUL	-0.1434753	-0.2867287	0.1033190	NA	NA	NA	
wfactor_slope_10_X_GLUL	0.6096741	-0.6951344	3.6819238	NA	NA	NA	
wfactor_point_2_X_GLUL	-0.4177153	-0.7950860	0.1801044	NA	NA	NA	
wfactor_rate_2_X_GLUL	0.1176258	-0.2145472	0.3000557	NA	NA	NA	
wfactor_slope_2_X_GLUL	-0.3974895	-0.6570468	0.5494805	NA	NA	NA	
wfactor_point_3_X_GLUL	0.0159012	-0.6729531	0.4672290	NA	NA	NA	

Continued on next page

parameter	estimate	CI.low	CI.high	p.value	p.value.adj	alpha.adj	sig
wfactor_rate_3_X_GLUL	0.2670023	0.0457308	0.4039659	NA	NA	NA	
wfactor_slope_3_X_GLUL	0.9639788	0.6990725	1.2946183	NA	NA	NA	
wfactor_point_4_X_GLUL	0.3346426	-0.7857640	0.7651047	NA	NA	NA	
wfactor_rate_4_X_GLUL	-0.0624301	-0.3338106	0.5063278	NA	NA	NA	
wfactor_slope_4_X_GLUL	0.6834172	-0.6987371	5.3789910	NA	NA	NA	
wfactor_point_5_X_GLUL	0.5424306	-0.7994472	0.7394209	NA	NA	NA	
wfactor_rate_5_X_GLUL	-0.2988603	-0.3826637	0.3978549	NA	NA	NA	
wfactor_slope_5_X_GLUL	-0.2533437	-0.8257310	1.4583524	NA	NA	NA	
wfactor_point_6_X_GLUL	-0.3055158	-0.7128864	0.4831647	NA	NA	NA	
wfactor_rate_6_X_GLUL	-0.3130196	-0.4355221	-0.1084834	NA	NA	NA	
wfactor_slope_6_X_GLUL	0.6056216	0.0431389	3.5288955	NA	NA	NA	
wfactor_point_7_X_GLUL	-0.6322173	-0.8894154	-0.4470122	NA	NA	NA	
wfactor_rate_7_X_GLUL	0.2381297	0.1205119	0.3847783	NA	NA	NA	
wfactor_slope_7_X_GLUL	0.0662331	-0.5584464	0.6253842	NA	NA	NA	
wfactor_point_8_X_GLUL	0.6867386	0.4323814	0.8250188	NA	NA	NA	
wfactor_rate_8_X_GLUL	0.1267014	-0.1526381	0.2971079	NA	NA	NA	
wfactor_slope_8_X_GLUL	7.2177844	0.6040240	9.2540008	NA	NA	NA	
wfactor_point_9_X_GLUL	0.5025911	-0.3357715	0.7529115	NA	NA	NA	
wfactor_rate_9_X_GLUL	0.1984204	-0.0773519	0.2916022	NA	NA	NA	
wfactor_slope_9_X_GLUL	2.2565347	0.0805888	4.7372170	NA	NA	NA	
wfactor_point_1_X_PCK1	0.2330195	0.2176610	0.3597981	NA	NA	NA	
wfactor_rate_1_X_PCK1	0.0515896	-0.0752829	0.1320635	NA	NA	NA	
wfactor_slope_1_X_PCK1	0.1705907	0.5640056	2.4958561	NA	NA	NA	
wfactor_point_10_X_PCK1	-0.0039846	-0.1646005	0.1647999	NA	NA	NA	
wfactor_rate_10_X_PCK1	-0.1826863	-0.2043623	-0.1243826	NA	NA	NA	
wfactor_slope_10_X_PCK1	2.3513793	0.0964185	1.5640916	NA	NA	NA	
wfactor_point_2_X_PCK1	0.2211287	0.1382506	0.3333648	NA	NA	NA	
wfactor_rate_2_X_PCK1	0.1103393	0.0634663	0.1674900	NA	NA	NA	
wfactor_slope_2_X_PCK1	1.7840359	1.3965520	3.8595372	NA	NA	NA	
wfactor_point_3_X_PCK1	0.0005758	0.0246923	0.3017108	NA	NA	NA	
wfactor_rate_3_X_PCK1	-0.1018003	-0.1228496	0.0856158	NA	NA	NA	
wfactor_slope_3_X_PCK1	0.5915321	-0.0763677	1.8903158	NA	NA	NA	
wfactor_point_4_X_PCK1	0.0853231	-0.1656315	0.4059631	NA	NA	NA	
wfactor_rate_4_X_PCK1	0.2748680	0.2536094	0.2877811	NA	NA	NA	
wfactor_slope_4_X_PCK1	0.1430527	0.2307655	1.4118296	NA	NA	NA	
wfactor_point_5_X_PCK1	0.3011909	0.1692790	0.4664308	NA	NA	NA	
wfactor_rate_5_X_PCK1	-0.2545560	-0.2774610	-0.2399148	NA	NA	NA	
wfactor_slope_5_X_PCK1	1.0249975	0.4313978	1.6019923	NA	NA	NA	
wfactor_point_6_X_PCK1	0.1116446	-0.0457983	0.5226237	NA	NA	NA	
wfactor_rate_6_X_PCK1	-0.1891536	-0.2172539	-0.1566733	NA	NA	NA	
wfactor_slope_6_X_PCK1	1.2011468	0.3205093	1.9485025	NA	NA	NA	
wfactor_point_7_X_PCK1	0.1715480	0.1871776	0.4523633	NA	NA	NA	
wfactor_rate_7_X_PCK1	0.3012152	0.2907144	0.3189858	NA	NA	NA	
wfactor_slope_7_X_PCK1	1.0930997	0.3804793	1.0925261	NA	NA	NA	
wfactor_point_8_X_PCK1	0.1840418	0.1055327	0.4344233	NA	NA	NA	
wfactor_rate_8_X_PCK1	-0.2018460	-0.2210964	-0.1688954	NA	NA	NA	
wfactor_slope_8_X_PCK1	1.1772945	0.0693788	0.3148323	NA	NA	NA	
wfactor_point_9_X_PCK1	0.0168746	-0.0468562	0.2669765	NA	NA	NA	
wfactor_rate_9_X_PCK1	0.1579260	0.1209957	0.2099166	NA	NA	NA	
wfactor_slope_9_X_PCK1	1.5781577	0.5635277	3.2133013	NA	NA	NA	
